# Structural and Electronic Studies of Substituted *m*-Terphenyl Group 12 Complexes

**DOI:** 10.1021/acs.organomet.2c00156

**Published:** 2022-05-30

**Authors:** Andrew J. Valentine, Laurence J. Taylor, Ana M. Geer, Cameron D. Huke, Katherine E. Wood, Will Tovey, William Lewis, Stephen P. Argent, Andrew M. Teale, Jonathan McMaster, Deborah L. Kays

**Affiliations:** † School of Chemistry, University of Nottingham, University Park, Nottingham NG7 2RD, U.K.; ‡ Departamento de Química Inorgánica, Instituto de Síntesis Química y Catálisis Homogénea (ISQCH), CSIC Universidad de Zaragoza, Pedro Cerbuna 12, 50009 Zaragoza, Spain; § School of Chemistry, The University of Sydney, F11, Eastern Avenue, Sydney, NSW 2006, Australia

## Abstract

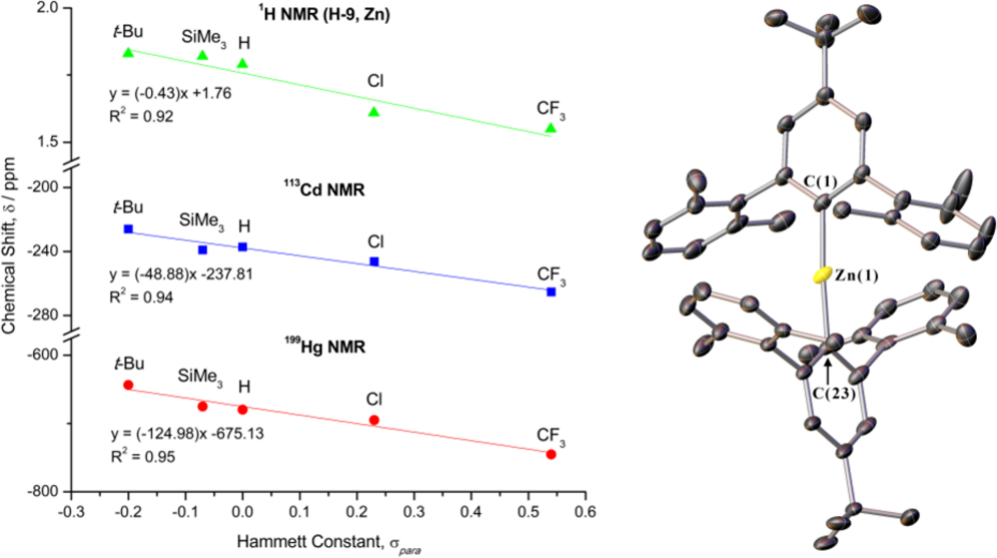

The effects of *para*-substitution on the structural and electronic properties of four series of two-coordinate *m*-terphenyl Group 12 complexes (R-Ar^#^)_2_M (M = Zn, Cd, Hg; R = *t*-Bu **1**–**3**, SiMe_3_**4**–**6**, Cl **7**–**9**, CF_3_**10**–**12**, where R-Ar^#^ = 2,6-{2,6-Xyl}_2_-4-R-C_6_H_2_ and 2,6-Xyl = 2,6-Me_2_C_6_H_3_) have been investigated. X-ray crystallography shows little structural variation across the series, with no significant change in the C–M–C bond distances and angles. However, considerable electronic differences are revealed by heteronuclear nuclear magnetic resonance (NMR) spectroscopy; a linear correlation is observed between the ^113^Cd, ^199^Hg, and ^1^H (2,6-Xyl methyl protons) NMR chemical shifts of the *para*-substituted complexes and the Hammett constants for the R-substituents. Specifically, an upfield shift in the NMR signal is observed with increasingly electron-withdrawing R-substituents. Density functional theory (DFT) calculations are employed to attempt to rationalize these trends.

## 1 Introduction

The stabilization of low-coordinate Group 12 metal complexes exhibiting novel bonding modes and geometries has been explored through the use of sterically demanding ligands.^1−6^ In contrast to the earliest examples of zinc, cadmium, and mercury dialkyl and diaryl complexes,^7−10^ which incorporate necessary secondary stabilizing interactions, the bulky *m*-terphenyl framework^11,12^ has enabled the isolation of strictly two-coordinate Group 12 systems such as (2,6-Mes_2_C_6_H_3_)_2_Zn (Mes = 2,4,6-Me_3_C_6_H_2_).^13,14^ Other reports include the synthesis of a homologous series of Group 12 M–M-bonded species (2,6-Dipp_2_C_6_H_3_)_2_M_2_ (M = Zn, Cd, Hg; Dipp = 2,6-*i*-Pr_2_C_6_H_3_)^15,16^ and the formation of a Zn–Zr–Zn unit in [(2,6-Tripp_2_C_6_H_3_)Zn]_2_Zr(η^5^-C_5_H_5_)_2_ (Tripp = 2,4,6-*i*-Pr_3_C_6_H_2_).^17,18^

The application of Group 12 organometallic complexes in catalysis has rendered them invaluable reagents for synthesis. Organozinc compounds, for example, have proven useful in organic transformations,^19,20^ alkali-metal-mediated zincation reactions,^21,22^ and copolymerization reactions.^23,24^ Organocadmium complexes, on the other hand, play a key role as molecular precursors in the synthesis of photoluminescent quantum dots,^25,26^ while organomercurials feature prominently as ligand transmetallation reagents.^27,28^

Previous work within our group has explored the structural role of the *m*-terphenyl ligand upon three series of two-coordinate Group 12 diaryls (2,6-Ar_2_C_6_H_3_)_2_M (M = Zn, Cd, Hg; Ar = 2,6-Xyl {2,6-Me_2_C_6_H_3_}, 3,5-Xyl {3,5-Me_2_C_6_H_3_}, Pmp {Me_5_C_6_}), where subtle changes in the steric pocket around the metal center were studied.^29^ Thus, the bulkier 2,6-Xyl and Pmp flanking groups led to near-linear C–M–C bond angles [175.78(12)–180.0(0)°], whereas the less sterically hindered 3,5-Xyl group resulted in greater deviations from linearity [171.18(5)–176.4(2)°]. However, the effects of varying the electronic structure of the *m*-terphenyl ligand upon these Group 12 compounds have yet to be investigated.

Multiple studies by Power et al. have analyzed the electronic properties of metal complexes incorporating *para*-substituted *m*-terphenyl ligands.^30−32^ One example is the quintuply-bonded arylchromium dimer, where a set of *para*-functionalized analogues [(2,6-Dipp_2_-4-R-C_6_H_2_)Cr]_2_ (R = H, SiMe_3_, OMe, F) were prepared to probe the nature of the Cr–Cr bond.^33^ Additional reports include the study of a series of *para*-substituted Group 14 complexes (2,6-Mes_2_-4-R-C_6_H_2_)_2_M (M = Ge, Sn, Pb; R = H, SiMe_3_, Cl)^34,35^ and the analysis of the functionalized tin hydrides [(2,6-Dipp_2_-4-R-C_6_H_2_)Sn(μ-H)]_2_ (R = H, SiMe_3_, OMe, *t*-Bu).^36^

We have employed a series of *para*-substituted *m*-terphenyl ligands to study the role of electronic effects on the structures, bonding, and electronic properties of their Group 12 diaryl complexes. The diamagnetic nature of these Group 12 metal(II) species means that their electronic structures may be probed by NMR spectroscopy, which has been used previously to differentiate between *syn*- and *anti*-conformers in a series of naphthyl-substituted complexes (2,6-Naph_2_C_6_H_3_)_2_M (M = Zn, Cd·OEt_2_, Hg·OEt_2_; Naph = 1-C_10_H_7_).^37^ Herein, four series of novel *para*-substituted, two-coordinate, *m*-terphenyl Group 12 diaryls (R-Ar^#^)_2_M (R-Ar^#^ = 2,6-{2,6-Xyl}_2_-4-R-C_6_H_2_; M = Zn, Cd, Hg; R = *t*-Bu, SiMe_3_, Cl, CF_3_) are reported and discussed alongside their unsubstituted analogues (H-Ar^#^)_2_M.^29^ The geometric and electronic properties of these compounds are elucidated through X-ray crystallographic and NMR spectroscopic studies, respectively. We employ ^113^Cd and ^199^Hg NMR spectroscopies to assess the impact of the variation of the electronic structure of the ligand directly at the metal center.

## 2 Results and Discussion

### 2.1 Synthesis

**Scheme 1:**
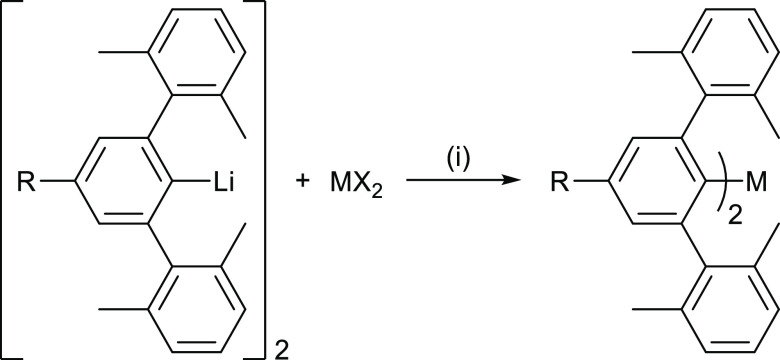
Synthesis of Diaryl Complexes (R-Ar^#^)_2_M (M = Zn, Cd, Hg; R = *t*-Bu **1**–**3**, SiMe_3_**4**–**6**, Cl **7**–**9**, CF_3_**10**–**12**), where MX_2_ Is ZnCl_2_, CdCl_2_, or HgBr_2_ Reaction conditions: (i) toluene/THF (10:1), room temperature, 16 h, −2 LiX.

The reaction between the lithium complexes [R-Ar^#^-Li]_2_ (R-Ar^#^ = 2,6-{2,6-Xyl}_2_-4-R-C_6_H_2_; R = *t*-Bu, SiMe_3_, Cl, CF_3_)^38^ with one equivalent of ZnCl_2_, CdCl_2_, or HgBr_2_ in a toluene/THF (10:1) mixture at room temperature yielded the Group 12 diaryl species (*t*-Bu-Ar^#^)_2_M (M = Zn **1**, Cd **2**, Hg **3**), (Me_3_Si-Ar^#^)_2_M (M = Zn **4**, Cd **5**, Hg **6**), (Cl-Ar^#^)_2_M (M = Zn **7**, Cd **8**, Hg **9**), and (F_3_C-Ar^#^)_2_M (M = Zn **10**, Cd **11**, Hg **12**) according to Scheme 1. Complexes **1**–**12** were recrystallized from a −30 °C *iso*-hexane solution to give colorless crystals in low-to-moderate isolated yields. Characterizations of **1**–**12** have been achieved by single-crystal X-ray diffraction, multinuclear (^1^H, ^13^C{^1^H}, ^19^F{^1^H}, ^29^Si{^1^H}, ^113^Cd and ^199^Hg) NMR spectroscopies, mass spectrometry, cyclic voltammetry (for **3** and **12**), and elemental analyses.

### 2.2 Solid-State Characterization

**Figure 1:**
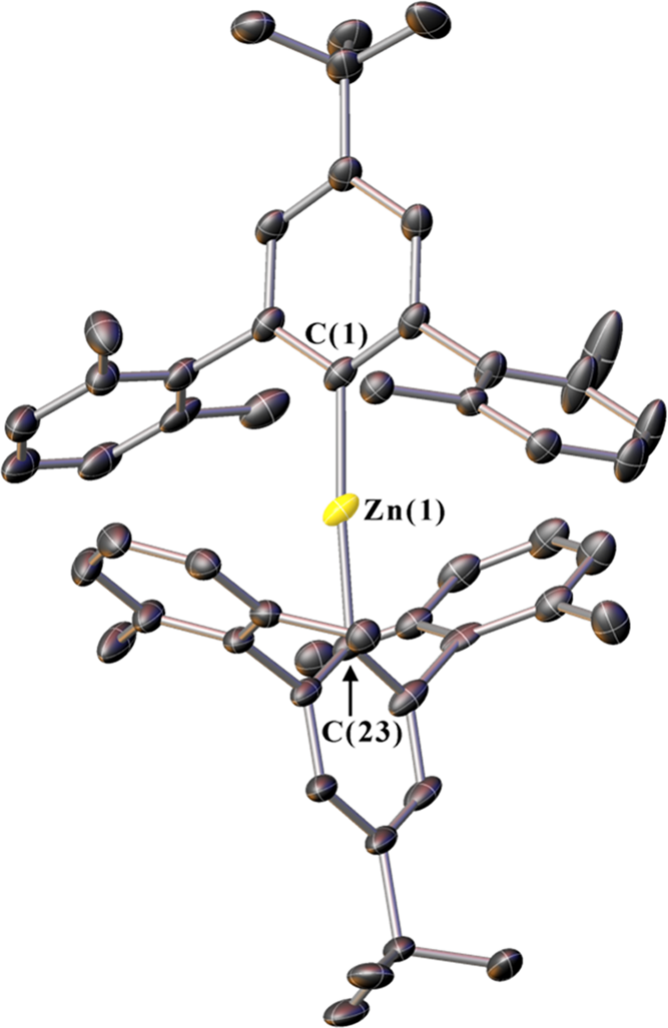
Crystal structure of **1**. Ellipsoids set at 30% probability. Disordered solvent and hydrogen atoms are omitted for clarity.

The crystal structures of **1**–**12** confirm that all complexes are monomeric in the solid state, owing to the steric demands of the *m*-terphenyl ligands, with no intermolecular interactions between the metal centers. In all cases, the complexes are two-coordinate and quasi-linear, featuring a single metal center coordinated by two σ-bonded *m*-terphenyl ligands. Unlike the 3,5-Xyl complexes [2,6-{3,5-Xyl}_2_C_6_H_3_]_2_M (M = Zn, Cd, Hg), no M···H contacts are formed to the flanking aryl rings.^29^ The crystal structure of **1** is presented in Figure 1, with key measurements about the metal center for **1**–**12** provided in Table 1. Full crystallographic data for **1**–**12** are provided in Supporting Information Figures S40–S43 and Table S1 (M = Zn), Table S2 (M = Cd), and Table S3 (M = Hg). It should be noted that the crystal data for **4** are of low quality due to weak diffraction from a small crystal. Despite repeated attempts, it was not possible to grow high-quality crystals of **4**. However, the data are sufficient to demonstrate the connectivity of the molecule and are included here for completeness.

**Table 1:** Selected Bond Lengths (Å) and Angles (deg) for **1**–**12**

**compound**	**M**	**R**	**M(1)–C(1)**	**M(1)–C(23)**	**C(1)–M1–C(23)**
**1**	Zn	*t-*Bu	1.937(2)	1.934(2)	175.87(10)
**2**	Cd	*t-*Bu	2.110(2)	2.110(2)	176.57(7)
**3**^a^	Hg	*t-*Bu	2.070(3)		175.91(13)
**4**^b^	Zn	SiMe_3_	1.953(12)	1.951(13)	176.4(6)
**5**	Cd	SiMe_3_	2.111(14)	2.098(14)	177.5(6)
**6**	Hg	SiMe_3_	2.056(10)	2.063(10)	177.1(4)
**7**^c^	Zn	Cl	1.9418(17) [1.9429(17)]	1.9465(17) [1.9367(17)]	176.10(8) [176.84(9)]
**8**	Cd	Cl	2.120(2)	2.116(2)	177.42(10)
**9**	Hg	Cl	2.086(3)	2.085(3)	177.51(14)
**10**	Zn	CF_3_	1.9449(13)	1.9483(13)	178.87(6)
**11**	Cd	CF_3_	2.1159(16)	2.1215(16)	179.16(6)
**12**	Hg	CF_3_	2.089(3)	2.098(3)	179.28(12)

a For **3**, C(1) = C(23) due to symmetry (*Z*′ = 0.5).

b Crystal data for **4** are of low quality due to weak diffraction from a very small crystal. Data are included here for completeness.

c Measurements for the second molecule in asymmetric unit given in square brackets.

For each Group 12 metal, the corresponding series of *para*-substituted complexes show no significant change in the M–C bond distances as the functional group is varied. The Zn–C bond distances for **1**, **4**, **7**, and **10** fall within a narrow range [1.934(2)–1.953(12) Å] and are comparable to the previously reported unsubstituted analogue (H-Ar^#^)_2_Zn [Zn(1)–C(1) = 1.949(4) Å, Zn(1)–C(23) = 1.944(4) Å].^29^ These values also correlate with other zinc diaryl complexes in the literature, whose Zn–C bond distances range between 1.93 and 1.95 Å.^39−41^

A narrow range of M–C bond distances is also observed for **2**, **5**, **8**, and **11** [Cd–C = 2.098(14)–2.1215(16) Å] and for **3**, **6**, **9**, and **12** [Hg–C = 2.056(10)–2.098(3) Å], which mirror those of the unsubstituted analogues (H-Ar^#^)_2_M (M = Cd, Hg) [Cd(1)–C(1) = 2.115(5) Å, Cd(1)–C(23) = 2.228(5) Å and Hg(1)–C(1) = 2.087(6) Å, Hg(1)–C(23) = 2.101(5) Å].^29^ These values are comparable to other cadmium and mercury diaryl complexes, whose M–C bond distances range between 2.11–2.12 and 2.07–2.15 Å, respectively.^10,42−46^ The reduction in M–C bond distance on moving from Cd to Hg can be attributed to a combination of relativistic effects and lanthanide contraction.^47−49^

The C–M–C angles for **1**–**12** also present a reasonably narrow range of values. Thus, the C–Zn–C angles of **1**, **4**, **7**, and **10** [175.87(10)–178.87(6)°] are comparable to the C–Cd–C angles of **2**, **5**, **8**, and **11** [176.57(7)–179.16(6)°] and to the C–Hg–C angles of **3**, **6**, **9**, and **12** [175.91(13)–179.28(12)°], indicating little variation as the metal is changed. These values correlate with the C–M–C angles reported for the unsubstituted analogues (H-Ar^#^)_2_M (M = Zn, Cd, Hg) [177.1(2)–179.9(3)°] but differ from the angles observed in the (less sterically hindered) 3,5-Xyl complexes (3,5-Xyl_2_C_6_H_3_)_2_M (M = Zn, Cd, Hg) [171.18(5)–176.4(2)°].^29^ The C–M–C angles for **1**–**12** are also similar to those of Mes_2_M (M = Zn, Cd, Hg).^9,10^

In summary, the crystal structures of **1**–**12** show little structural variation as the *para*-substituent of the *m-*terphenyl ligand is varied. This suggests that the geometries of these complexes are dominated by steric and crystal packing effects, rather than the electronic structure of the ligand.

### 2.3 Solution-State Characterization

**Figure 2:**
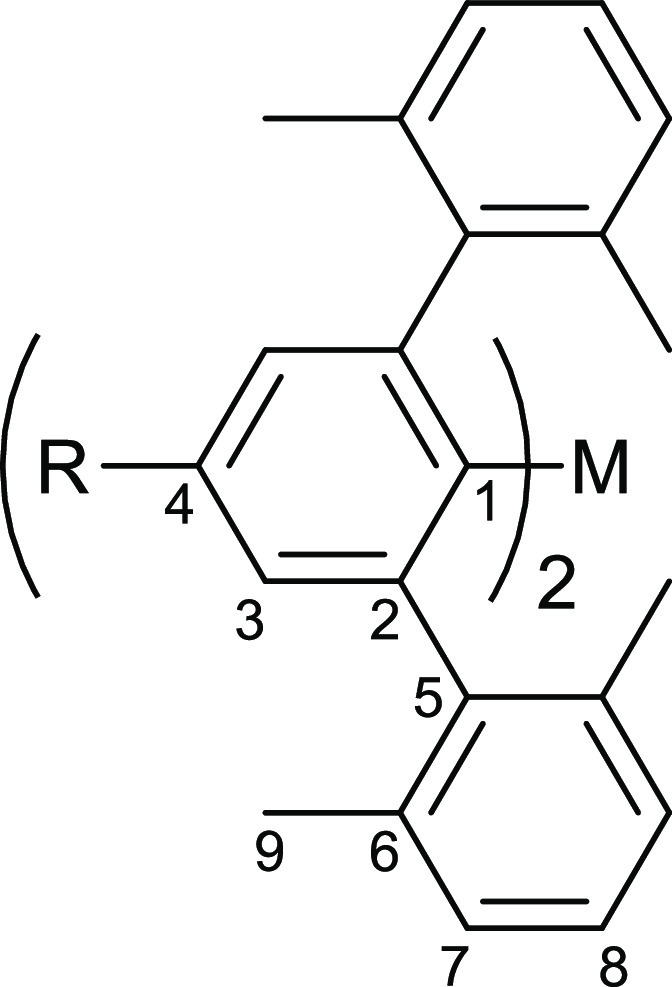
NMR numbering scheme for *m*-terphenyl complexes **1**–**12**.

**Figure 3:**
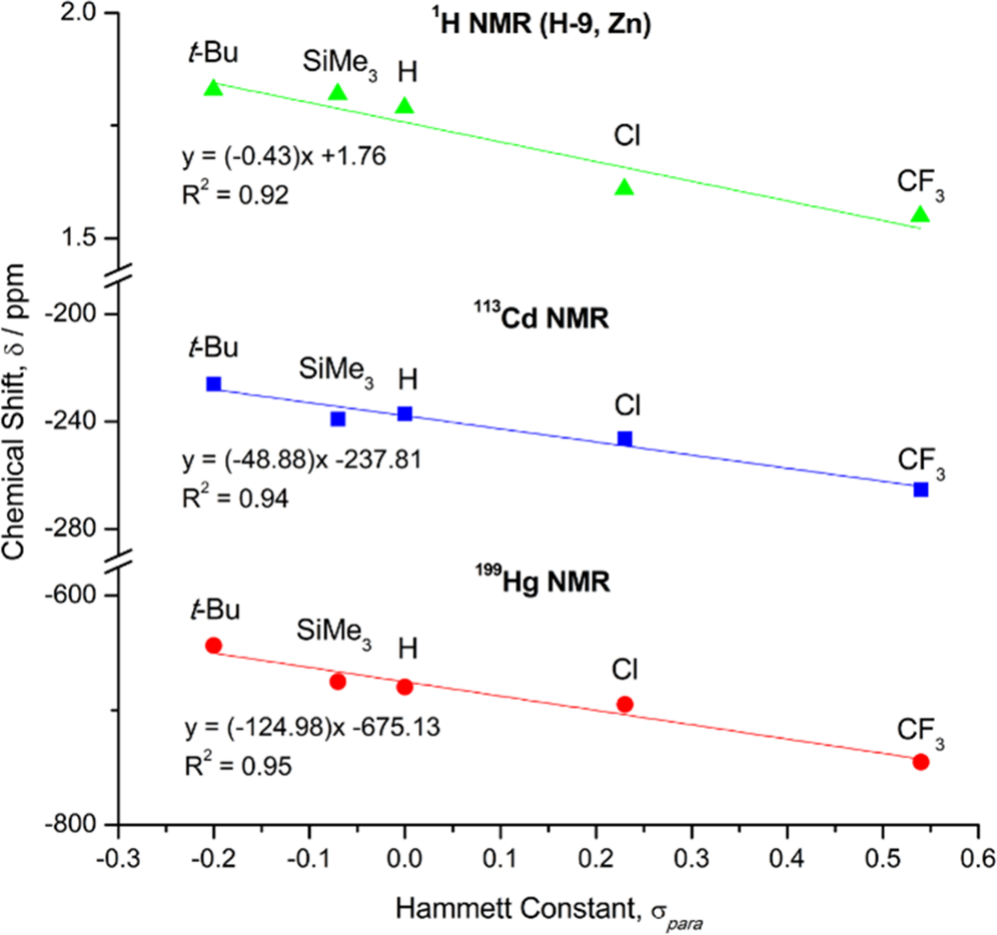
Plot of the ^1^H (for flanking methyl protons, H-9), ^113^Cd, and ^199^Hg NMR chemical shifts, δ, for the metal diaryls (R-Ar^#^)_2_M (**1**–**12**, plus R = H)^29^ vs their Hammett constants, σ_para_.^50^ For clarity, the ^1^H NMR (H-9) trend is given only for the Zn series; plots for the Cd and Hg series are provided in Supporting Information Figure S1.

The electronic structures of **1**–**12** were studied by ^1^H, ^13^C{^1^H}, ^113^Cd, and ^199^Hg NMR spectroscopies in *d*_6_-benzene and compared to those of the unsubstituted analogues (H-Ar^#^)_2_M (M = Zn, Cd, Hg).^29^ Here, a numbering scheme has been assigned to the *m*-terphenyl unit, as shown in Figure 2. The electronic strengths of different *para*-substituents are quantified using Hammett constants, σ_para_.^50^ A comparison of the ^1^H NMR spectra for complexes **1–12** reveals three noteworthy features (Table 2). First, the *meta*-protons (H-3) on the central aryl rings exhibit notable peak shifts as the *para*-substituent is changed, although no overall trend is evident. There is, however, a clear downfield shift in H-3 when varying the metal from Zn (6.76–7.14 ppm) to Cd (6.87–7.22 ppm) to Hg (6.92–7.30 ppm). Second, the 2,6-Xyl aryl protons (H-7 and H-8) for **1**–**12** remain relatively unshifted by changing the *para*-substituent or the metal, suggesting there is minimal electronic communication with the flanking aryl rings. Third, the 2,6-Xyl methyl protons (H-9) shift upfield with increased electron-withdrawing strength of the *para*-substituent. A plot of the chemical shifts, δ, against the Hammett constants, σ_para_, reveals a linear correlation (Figures 3 and S1).^50^ A similar trend was observed in recent studies of the analogous lithium complexes [R-Ar^#^-Li]_2_ (R = *t*-Bu, SiMe_3_, H, Cl, CF_3_).^38^ We note that the chemical shifts for H-9 are largely unaffected by the identity of the metal (Table 2).

**Table 2:** Relevant ^1^H and ^13^C{^1^H} NMR Chemical Shifts, δ, for the Metal Diaryls (R-Ar^#^)_2_M (**1**–**12**, plus R = H)^29^ a

			^1^H and ^13^C{^1^H} NMR chemical shifts, δ (ppm)						
	(R-Ar^#^)_2_M	R group	H-3	H-9	C-1	C-2	C-3	C-4	C-9
M = Zn	**1**	*t*-Bu	6.93	1.83	148.5	149.8	122.4	151.9	21.9
	**4**	SiMe_3_	7.14	1.82	152.8	149.3	130.2	140.7	22.0
	lit.^b^	H	6.76	1.79	152.0	150.0	125.5	128.9	21.8
	**7**	Cl	6.78	1.61	150.2	151.6	125.7	135.4	21.6
	**10**	CF_3_	7.05	1.55	156.8	150.7	122.1	131.5	21.6
M = Cd	**2**	*t*-Bu	7.02	1.84	158.3	149.4	122.0	151.4	21.5
	**5**	SiMe_3_	7.22	1.82	162.9	149.1	129.7	140.0	21.6
	lit.^b^	H	6.87	1.80	161.9	149.6	125.1	128.4	21.5
	**8**	Cl	6.88	1.63	160.1	151.1	125.4	134.8	21.2
	**11**	CF_3_	7.14	1.56	167.0	150.2	121.7	131.1	21.3
M = Hg	**3**	*t*-Bu	7.09	1.83	169.1	148.4	123.7	151.4	21.5
	**6**	SiMe_3_	7.30	1.81	173.0	148.1	131.5	140.2	21.5
	lit.^b^	H	6.92	1.78	172.0	148.7	126.8	128.4	21.4
	**9**	Cl	6.92	1.61	170.2	150.1	127.0	134.7	21.2
	**12**	CF_3_	7.20	1.54	176.0	149.3	123.6	131.1	21.2

a The flanking aryl atoms remain unshifted and thus have been omitted.

b Literature NMR data for the unsubstituted complexes (H-Ar^#^)_2_M (M = Zn, Cd, Hg), original data re-referenced to C_6_D_6_.^29^

The ^13^C{^1^H} NMR spectra of **1**–**12** show nine peaks for the carbons of the ligand framework, as well as additional peaks for the C-atoms of the *t*-Bu, SiMe_3_, and CF_3_ groups. A comparison of the spectra reveals that C-5 (143.2–147.4 ppm), C-6 (135.8–136.4 ppm), C-7 (127.8–128.3 ppm), C-8 (127.1–127.9 ppm), and C-9 (21.2–22.0 ppm) of the 2,6-Xyl groups remain relatively unshifted, irrespective of the nature of the metal or *para*-group (Table 2). This can again be attributed to poor electronic communication between the central and flanking aryl rings. However, the ^13^C{^1^H} NMR signals for the central aryl ring shift considerably with the notable exception of C-2 (Table 2). We note that the largest shifts are for the *ipso*-carbon atoms (C-1) where, in addition to a downfield shift in δ_C_ with increasing σ_para_ of the substituent, large downfield shifts of ca. 10 ppm are observed as the metal varies from Zn (148.5–156.8 ppm) to Cd (158.3–167.0 ppm) to Hg (169.1–176.0 ppm). For similar complexes in the literature, this downfield trend has been ascribed to the increasing Pauling electronegativity as Group 12 is descended (1.65, 1.69, and 2.00 for Zn, Cd, and Hg, respectively).^10,16,29,46,51−53^

The ^113^Cd and ^199^Hg NMR spectra of **2**, **5**, **8**, **11** and **3**, **6**, **9**, **12** were also recorded. Multiple NMR measurements revealed no change in chemical shift with varying analyte concentration, most likely due to the steric bulk of the ligands preventing interaction of the metal with the surrounding solvent.^54−56^ In all cases, the ^113^Cd and ^199^Hg NMR spectra show a single peak indicating one metal environment in solution, in the same region as other literature metal diaryl complexes (see Table 3).^37,57−59^ In previous work on the Group 12 diaryls (2,6-Ar_2_C_6_H_3_)_2_M (M = Cd, Hg; Ar = 2,6-Xyl, 3,5-Xyl, Pmp), increasing the steric bulk of the flanking groups was found to cause an upfield shift in their ^113^Cd and ^199^Hg NMR spectra.^29^ However, since complexes **1**–**12** all feature the same flanking groups (2,6-Xyl) and are crystallographically similar, we suggest that steric effects are unlikely to have a major influence on their ^113^Cd and ^199^Hg NMR shifts.

**Table 3:** ^113^Cd and ^199^Hg NMR Chemical Shifts, δ, for the Metal Diaryls (R-Ar^#^)_2_M (M = Cd, Hg; R = *t*-Bu **2**–**3**, SiMe_3_**5**–**6**, H,^29^ Cl **8**–**9**, CF_3_**11**–**12**)^50^

				NMR chemical shifts, δ (ppm)	
	(R-Ar^#^)_2_M	R group	Hammett constant, σ_para_	^113^Cd	^199^Hg
M = Cd, Hg	**2**, **3**	*t*-Bu	–0.20	–225.89	–642.81
	**5**, **6**	SiMe_3_	–0.07	–239.07	–674.91
	lit.^a^	H	0.00	–239.36	–679.77
	**8**, **9**	Cl	0.23	–246.03	–695.04
	**11**, **12**	CF_3_	0.54	–265.21	–745.00

a Literature NMR data for the unsubstituted complexes (H-Ar)_2_M (M = Cd, Hg).^29,50^

A plot of the ^113^Cd and ^199^Hg NMR chemical shifts (δ) for each of the *para*-substituted complexes, vs their corresponding Hammett constant (σ_para_) is shown in Figure 3.^50^ Linear correlations can be fitted to the ^113^Cd (blue line; *R*^2^ = 0.96) and ^199^Hg (red line; *R*^2^ = 0.95) NMR data, both with a negative gradient, indicating that more electron-withdrawing substituents shift the NMR peak of the Cd and Hg centers further upfield. This trend is somewhat counterintuitive, as electron-withdrawing groups might be expected to deshield the nuclei and cause a downfield shift. However, similar findings were reported for a series of *para*-substituted mercury diaryls (4-R-C_6_H_4_)_2_Hg (R = OMe, Me, H, F, Cl, CF_3_),^60−62^ suggesting that these chemical shifts depend on more than simple σ donor effects. One hypothesis suggests that the bonding in organomercury compounds mainly involves the valence 6s orbital^63,64^ since the 6p orbital is too high in energy to overlap. However, by incorporating electron-donating groups onto the ligand, the ligand orbitals increase in energy and overlap better with the 6p orbitals.^58,65^ This populates the more diffuse 6p orbitals and depopulates the less diffuse 6s. Hence, the electron density around the metal center moves away from the nucleus and becomes more diffuse, resulting in less shielding and a downfield NMR shift.^58^

Cyclic voltammetry studies were also carried out on the mercury complexes **3** and **12** (R = *t-*Bu and CF_3_) in THF solution (Supporting Information, Section S4). However, no redox events were observed upon scanning from −0.5 to −2.5 V (vs Fc^+^/Fc) in either case (Figure S44), suggesting a large HOMO–LUMO gap for these complexes.

### 2.4 Computational Analysis

Density functional theory (DFT) calculations were employed to attempt to rationalize the trends in the NMR spectroscopic parameters. Full geometry optimizations (BP86/TZVP, see Supporting Information Section S5.1 for full details) were performed on **1**–**12**, as well as the unsubstituted analogues. All optimized structures displayed near-linear bond angles in a very narrow range (Table S5), although structures with M = Zn showed slightly greater distortion from linearity (C–Zn–C = 178.18–178.88°; C–Cd–C = 179.08–179.90°; C–Hg–C = 179.57–179.88°). Single-point calculations (PBE0/TZVP, see Supporting Information Section S5.2 for full details) were then performed on the optimized structures to obtain an estimate of the orbital energies. This showed that the HOMO energies, LUMO energies, and HOMO–LUMO gap all show negative correlation with σ_para_ (Figures S45–S47). The predicted HOMO–LUMO gap (5.1–5.6 eV) is large enough to account for the observed lack of redox events over the potential range −0.5 to −2.5 V vs Fc^+^/Fc in the electrochemical experiments (see above).

A Quantum Theory of Atoms in Molecules (QTAIM) analysis was also employed on the optimized structures of (2,6-Xyl_2_C_6_H_3_)_2_M (M = Zn, Cd, Hg; see Supporting Information Section S5.2 for details). This analysis did not locate any bond paths corresponding to C–H···M (M = Zn, Cd, Hg) agostic interactions, which might have accounted for the observed trend in the H-9 chemical shifts. This contrasts with the recently reported dimeric lithium complexes [R-Ar^#^-Li]_2_ (R = *t*-Bu, SiMe_3_, H, Cl, CF_3_), where a trend in the ^1^H NMR chemical shifts of equivalent protons was linked to C–H···Li agostic interactions.^38^ However, for the Group 12 complexes, bond paths corresponding to C–H···C_arene_ interactions were observed between the H-9 protons and aromatic carbons of the flanking aryl rings situated opposite to them (Figure S48). Properties of the electron density at the bond critical points for these interactions are provided in Supporting Information Table S6.

Subsequently, the ^1^H, ^113^Cd, and ^199^Hg NMR chemical shift parameters for **1**–**12** and the unsubstituted analogues were calculated using the ReSpect program.^66−71^ These calculations were carried out on both the fully optimized structures used above, as well as the structures taken directly from the crystallographic data in which only the H atom positions had been optimized (see Supporting Information Section S5.1 for details). NMR shielding constants were calculated using the KT2 density functional approximation,^72^ which was specifically designed for the calculation of NMR shielding constants. The calculations were carried out at two levels of theory: dyall-vdz^73,74^ basis set for Zn/Cd/Hg and pcS-1^75^ for all other atoms (vdz/pcS-1) or dyall-vtz^73^ for Zn/Cd and pcS-2^75^ for all other atoms (vtz/pcS-2). Calculations for the mercury complexes at the vtz/pcS-2 level could not be completed due to technical limitations of the ReSpect program.^66−71^

A summary of the calculated ^1^H, ^113^Cd, and ^119^Hg NMR chemical shifts for the H-9 protons of **1**–**12** (in both the fully optimized and H-atom optimized geometries) are provided in Supporting Information Tables S9 and S10. Plots of the computed vs experimental shifts are shown in Supporting Information Figures S49–S56. In these, a weak positive correlation is observed between calculated and experimental shifts for the H-9 protons of all complexes (Figures S49–S53). This trend is evident in both the fully optimized and H-atom optimized structures and at both the vdz/pcS-1 and vtz/pcS-2 levels. However, the correlation is not particularly strong, and some computed results [particularly (H-Ar^#^)_2_Zn] deviate significantly from the experimental values. The experimental trend in ^1^H NMR shifts for the H-9 protons occurs over such a narrow chemical shift range (ca. 0.3 ppm) that the accuracy of the DFT calculations may not be sufficient to reliably reproduce this behavior. Despite the lack of C–H···M (M = Zn, Cd, Hg) close contacts, the H-9 chemical shifts feature large paramagnetic contributions to the shielding constant (Tables S7 and S8), much like the analogous lithium complexes [R-Ar^#^-Li]_2_ (R = *t*-Bu, SiMe_3_, H, Cl, CF_3_).^38^ It is known that when the paramagnetic components are dominant, density functional methods often fail to achieve high accuracy, as appears to be the case here.

The computed ^113^Cd and ^199^Hg NMR chemical shifts (vdz/pcS-1) show relatively poor agreement with the experimental values. While the ^113^Cd NMR shifts for the H-atom optimized structures appear to roughly correlate with the experimental values (Figure S54), this correlation is lost in the fully geometry optimized structures. No convincing correlation is observed for the ^199^Hg shifts in either geometry (Figure S56). In addition, the computed chemical shifts differ significantly (by >100 ppm) from the experimental shifts in all cases. At the vtz/pcS-2 level, the computed ^113^Cd shifts follow a similar trend relative to the experimental shifts as at the vdz/pcS-1 level (Figure S55), but the absolute values of the computed chemical shifts are closer to the experimental values.

These results suggest that the computed chemical shifts are strongly dependent on geometry, with small changes in the coordination environment of the metal resulting in dramatic changes in the computed shift. We propose that to model the NMR properties of these complexes more accurately, it may be necessary to perform dynamics calculations and account for conformational flexibility.

## 3 Conclusions

Four series of *para*-substituted *m*-terphenyl Group 12 complexes (R-Ar^#^)_2_M (M = Zn, Cd, Hg; R = *t*-Bu **1**–**3**, SiMe_3_**4**–**6**, Cl **7**–**9**, CF_3_**10**–**12**) have been reported. While negligible structural differences are observed by X-ray crystallography, NMR spectroscopic studies reveal considerable electronic differences within the ligand framework and at the metal center. A linear correlation of the ^113^Cd and ^199^Hg NMR chemical shifts is observed with the Hammett constants of the *para*-groups. Moreover, the flanking methyl protons, H-9, exhibit similar shifts in their ^1^H NMR spectra. In all cases, an upfield shift is observed with increasingly electron-withdrawing substituents. DFT modeling suggests that the H-9 ^1^H NMR chemical shifts, as well as the ^113^Cd and ^199^Hg chemical shifts, all feature large paramagnetic contributions to the shielding constants. As a result, the experimental trends could not be reproduced by our computational analysis.

## Supporting Information Available

Full experimental details for the synthesis, characterization, and crystallographic data (PDF)
Coordinates (XYZ)

## Supplementary Material

om2c00156_si_001.pdf

om2c00156_si_002.xyz

## Accession Codes

CCDC 2163371–2163382 contain the supplementary crystallographic data for this paper. These data can be obtained free of charge via www.ccdc.cam.ac.uk/data_request/cif, or by emailing data_request@ccdc.cam.ac.uk, or by contacting The Cambridge Crystallographic Data Centre, 12 Union Road, Cambridge CB2 1EZ, UK; fax: +44 1223 336033.
