# Supplementary material for: Structural and Electronic Studies of Substituted m-Terphenyl Group 12 Complexes
Source: Organometallics. 2022 May 30;41(11):1426–33. doi: 10.1021/acs.organomet.2c00156 (PMC9490839; doi:10.1021/acs.organomet.2c00156)
Supplement: Supplementary file 1 — om2c00156_si_001.pdf [file om2c00156_si_001.pdf]

Supporting Information for:

Structural and Electronic Studies of Substituted *m*-Terphenyl Group 12 Complexes

Andrew J. Valentine,[a] Laurence J. Taylor,[a] Ana M. Geer,[b] Cameron D. Huke,[a] Katherine E. Wood,[a] Will Tovey,[a] William Lewis,[c] Stephen P. Argent,[a] Andrew M. Teale,[a] Jonathan McMaster[a,]* and Deborah L. Kays[a,]*

[a] School of Chemistry, University of Nottingham, University Park, Nottingham, UK, NG7 2RD. E-mail: Deborah.Kays@nottingham.ac.uk; Jonathan.McMaster@nottingham.ac.uk

[b] Departamento de Química Inorgánica, Instituto de Síntesis Química y Catálisis Homogénea (ISQCH), CSIC Universidad de Zaragoza, Pedro Cerbuna 12, 50009 Zaragoza (Spain)

[c] School of Chemistry, The University of Sydney, F11, Eastern Ave, Sydney NSW 2006, Australia.

* Corresponding author

Contents

S1. Experimental 3

S1.1. General 3

S1.2. Syntheses 4

S1.2.1. Synthesis of (t-Bu-Ar#)$_2$M (M = Zn 1, Cd 2, Hg 3) 4

S1.2.2. Synthesis of (Me_3Si-Ar#)$_2$M (M = Zn 4, Cd 5, Hg 6) 5

S1.2.3. Synthesis of (Cl-Ar#)$_2$M (M = Zn 7, Cd 8, Hg 9) 6

S1.2.4. Synthesis of (F_3C-Ar#)$_2$M (M = Zn 10, Cd 11, Hg 12) 7

S2. Supplementary NMR Data 9

S2.1. (*t*-Bu-Ar#)$_2$Zn (1) 10

S2.2. (*t*-Bu-Ar#)$_2$Cd (2) 11

S2.3. (*t*-Bu-Ar#)$_2$Hg (3) 12

S2.4. (Me_3Si-Ar#)$_2$Zn (4) 14

S2.5. (Me_3Si-Ar#)$_2$Cd (5) 15

S2.6. (Me_3Si-Ar#)$_2$Hg (6) 17

S2.7. (Cl-Ar#)$_2$Zn (7) 19

S2.8. (Cl-Ar#)$_2$Cd (8) 20

S2.9. (Cl-Ar#)$_2$Hg (9) 22

S2.10. (F_3C-Ar#)$_2$Zn (10) 23

S2.11. (F_3C-Ar#)$_2$Cd (11) 25

S2.12. (F_3C-Ar#)$_2$Hg (12) 27

S3. Crystallography 30

S3.1. Crystallographic Methodology 30

S3.2. Crystallographic Data 31

S4. Cyclic Voltammetry 40

S5. Computational Work 41

S5.1. Geometry Optimization Methodology 41

S5.2. Orbital Energy and QTAIM Calculations 43

S5.2.1. Methodology 43

S5.2.2. Orbital Energy Plots 43

S5.2.3. QTAIM Analysis 45

S5.3. NMR Spectroscopic Calculations 47

S5.3.1. Methodology 47

S5.3.2. Results 49

S6. References 57

S1. Experimental

S1.1. General

All air-sensitive manipulations were performed using standard Schlenk line and glovebox techniques under an atmosphere of argon or nitrogen respectively. All solvents were pre-dried, either via passage through a drying column of 4 Å molecular sieves (*iso*-hexane), or via distilling over molten potassium (toluene) or sodium/benzophenone (THF). The solvents were stored over a potassium mirror (*iso*-hexane, toluene) or 4 Å molecular sieves (THF), and degassed *in vacuo* prior to use. Benzene-d_6 was dried over potassium and degassed via three freeze-pump-thaw cycles. Anhydrous $ZnCl_2$ and $CdCl_2$ were prepared by minor modifications of literature methods.[1] Organolithium ligand precursors $[R\text{-}Ar^{\#}\text{-}Li]_2$ (R = *t*-Bu, $SiMe_3$, Cl, CF_3; where $R\text{-}Ar^{\#}$ = 2,6-{2,6-Xyl}$_2$-4-R-C_6H_2 and 2,6-Xyl = 2,6-$Me_2C_6H_3$) were prepared according to previously published methods.[2] All other reagents and solvents were acquired commercially and used as received unless otherwise stated. ^{1}H, $^{13}C\{^{1}H\}$, $^{19}F\{^{1}H\}$, $^{29}Si\{^{1}H\}$, ^{113}Cd and ^{199}Hg NMR spectra were recorded using Bruker DPX300, AV400, AV(III)400, and AV(III)500 spectrometers. Chemical shifts are quoted in ppm relative to TMS (for ^{1}H, $^{13}C\{1H\}$ and $^{29}Si\{^{1}H\}$ NMR), to $CFCl_3$ (for $^{19}F\{^{1}H\}$ NMR), to $CdMe_2$ and $HgMe_2$ (for ^{113}Cd and ^{199}Hg NMR, respectively) using 0.1 M $Cd(ClO_4)_2/D_2O$ and 1.0 M $Hg(ClO_4)_2/D_2O$ solutions as external calibrants. Elemental analyses (CHN) were performed by Mr Stephen Boyer of the Microanalysis Service at the London Metropolitan University. (**Note:** CHN analysis was not performed on organomercury compounds due to concerns about mercury contamination of the instruments.) Mass spectrometry was carried out by Dr Mick Cooper at the University of Nottingham. (**Caution**: cadmium and mercury compounds are highly toxic and great care must be taken in their manipulation.)

S1.2. Syntheses

S1.2.1. Synthesis of (t-Bu-Ar#)$_2$M (M = Zn 1, Cd 2, Hg 3)

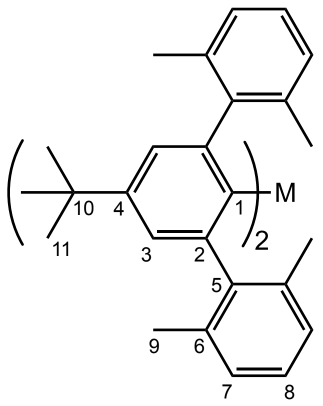

A mixture of [*t*-Bu-Ar#-Li]$_2$ (100 mg, 0.14 mmol) and MX_2 (0.14 mmol, MX_2 = $ZnCl_2$, $CdCl_2$, $HgBr_2$) in toluene (10 mL) and THF (1 mL) was stirred for 16 h at room temperature. After this time, the solvent was removed under vacuum to yield a white solid, which was dried at 60 °C under vacuum for 4 h, then extracted into hexane (3 x 10 mL). Concentration of the resultant colorless solution under vacuum, followed by storage at –30 °C, resulted in clear, colorless crystals of **1**–**3** with isolated yields of 35.0 mg (33%), 17.2 mg (15%) and 39.1 mg (31%) respectively.

Data for **1**: Elemental analysis: (Found: C, 83.3; H, 7.8. Calc. for $C_{52}H_{58}Zn$: C, 83.45; H, 7.8%); ^{1}H NMR δ_H (400 MHz; C_6D_6): 7.05 (12H, m, 8 x H-7 and 4 x H-8), 6.93 (4H, s, 4 x H-3), 1.83 (24H, s, 24 x H-9), 1.14 (18H, s, 18 x H-11); ^{13}C NMR δ_C (101 MHz; C_6D_6): 151.9 (C-4), 149.8 (C-2), 148.5 (C-1), 146.6 (C-5), 136.3 (C-6), 128.1 (C-7), 127.2 (C-8), 122.4 (C-3), 34.6 (C-10), 31.4 (C-11), 21.9 (C-9); HRMS (EI), m/z: (Found: 746.3817. Calc. for $C_{52}H_{58}Zn$: 746.3825.)

Data for **2**: Elemental analysis: (Found: C, 78.4; H, 7.6. Calc. for $C_{52}H_{58}Cd$: C, 78.5; H, 7.35%); ^{1}H NMR δ_H (400 MHz; C_6D_6): 7.08–7.04 (4H, m, 4 x H-8), 7.03–7.01 (8H, m, 8 x H-7), 7.02 (4H, s, 4 x H-3), 1.84 (24H, s, 24 x H-9), 1.18 (18H, s, 18 x H-11); ^{13}C NMR δ_C (101 MHz; C_6D_6): 158.3 (C-1), 151.4 (C-4) , 149.4 (C-2), 147.4 (C-5), 136.1 (C-6), 128.0 (C-7), 127.1 (C-8), 122.0 (C-3), 34.7 (C-10), 31.5 (C-11), 21.5 (C-9); ^{113}Cd NMR δ_{Cd} (88.8 MHz; C_6D_6): –225.89 (m); HRMS (EI), m/z: (Found: 796.3577. Calc. for $C_{52}H_{58}Cd$: 796.3567.)

Data for **3**: Elemental analysis: ^{1}H NMR δ_H (400 MHz; C_6D_6): 7.10–7.06 (4H, m, 4 x H-8), 7.09 (4H, s, 4 x H-3), 7.05–7.03 (8H, m, 8 x H-7), 1.83 (24H, s, 24 x H-9), 1.15 (18H, s, 18 x H-11); ^{13}C NMR δ_C (101 MHz; C_6D_6): 169.1 (C-1), 151.4 (C-4), 148.4 (C-2), 145.5 (C-5), 136.4 (C-6), 127.8 (C-7),

127.2 (C-8), 123.7 (C-3), 34.6 (C-10), 31.5 (C-11), 21.5 (C-9); ^{199}Hg NMR δ_{Hg} (71.7 MHz; C_6D_6): –642.81 (s); HRMS (EI), m/z: (Found: 884.4244. Calc. for $C_{52}H_{58}Hg$: 884.4239.)

S1.2.2. Synthesis of $(Me_3Si\text{-}Ar^{\#})_2M$ (M = Zn 4, Cd 5, Hg 6)

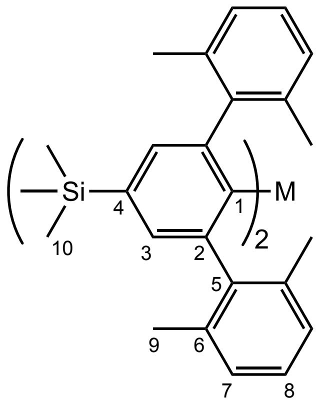

A mixture of $[Me_3Si\text{-}Ar^{\#}\text{-}Li]_2$ (100 mg, 0.14 mmol) and MX_2 (0.14 mmol, MX_2 = $ZnCl_2$, $CdCl_2$, $HgBr_2$) in toluene (10 mL) and THF (1 mL) was stirred for 16 h at room temperature. After this time, the solvent was removed under vacuum to yield a white solid, which was dried at 60 °C under vacuum for 4 h, then extracted into hexane (3 x 10 mL). Concentration of the resultant colorless solution under vacuum, followed by storage at –30 °C, resulted in clear, colorless crystals of **4**–**6** with isolated yields of 43.8 mg (41%), 29.2 mg (26%) and 35.0 mg (29%) respectively.

Data for **4**: Elemental analysis: (Found: C, 72.41; H, 7.32. Calc. for $C_{50}H_{58}Si_2Cd$: C, 72.55; H, 7.32%); ^{1}H NMR δ_H (400 MHz; C_6D_6): 7.14 (4H, s, 4 x H-3), 7.06–7.03 (4H, m, 4 x H-8), 7.03–7.00 (8H, m, 8 x H-7), 1.82 (24H, s, 24 x H-9), 0.13 (18H, s, 18 x H-10); ^{13}C NMR δ_C (101 MHz; C_6D_6): 152.8 (C-1), 149.3 (C-2), 146.3 (C-5), 140.7 (C-4), 136.3 (C-6), 130.2 (C-3), 128.2 (C-7), 127.3 (C-8), 22.0 (C-9), –1.1 (C-10); ^{29}Si NMR δ_{Si} (79.5 MHz; C_6D_6): –4.76 (s); HRMS (EI), m/z: (Found: 778.3341. Calc. for $C_{50}H_{58}Si_2Zn$: 778.3363.)

Data for **5**: Elemental analysis: (Found: C, 72.5; H, 7.2. Calc. for $C_{50}H_{58}Si_2Cd$: C, 72.6; H, 7.1%); ^{1}H NMR δ_H (400 MHz; C_6D_6): 7.22 (4H, s, 4 x H-3), 7.06–7.02 (4H, m, 4 x H-8), 7.02–6.99 (8H, m, 8 x H-7), 1.82 (24H, s, 24 x H-9), 0.17 (18H, s, 18 x H-10); ^{13}C NMR δ_C (101 MHz; C_6D_6): 162.9 (C-1), 149.1 (C-2), 147.2 (C-5), 140.0 (C-4), 136.1 (C-6), 129.7 (C-3), 128.1 (C-7), 127.2 (C-8), 21.6 (C-9), –0.9 (C-10); ^{29}Si NMR δ_{Si} (79.5 MHz; C_6D_6): –4.76 (s); ^{113}Cd NMR δ_{Cd} (88.8 MHz; C_6D_6): –239.07 (m); HRMS (FD), m/z: (Found: 828.3119. Calc. for $C_{50}H_{58}Si_2Cd$: 828.3118.)

Data for **6**: ^{1}H NMR δ_H (400 MHz; C_6D_6): 7.30 (4H, s, 4 x H-3), 7.08–7.05 (4H, m, 4 x H-8), 7.03–7.01 (8H, m, 8 x H-7), 1.81 (24H, s, 24 x H-9), 0.14 (18H, s, 18 x H-10); ^{13}C NMR δ_C (101 MHz; C_6D_6): 173.0 (C-1), 148.1 (C-2), 145.2 (C-5), 140.2 (C-4), 136.4 (C-6), 131.5 (C-3), 127.9 (C-7), 127.3 (C-8), 21.5 (C-9), –1.0 (C-10); ^{29}Si NMR δ_{Si} (79.5 MHz; C_6D_6): –4.69 (s); ^{199}Hg NMR δ_{Hg} (71.7 MHz; C_6D_6): –674.91 (s); HRMS (FD), m/z: (Found: 916.3789. Calc. for $C_{50}H_{58}Si_2Hg$: 916.3778.)

S1.2.3. Synthesis of (Cl-Ar$^{\#}$)$_2$M (M = Zn 7, Cd 8, Hg 9)

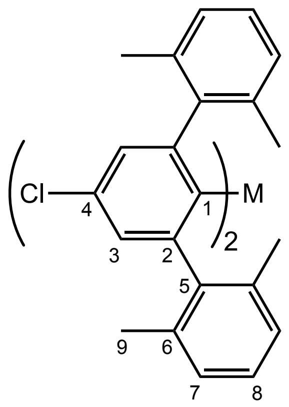

A mixture of [Cl-Ar$^{\#}$-Li]$_2$ (200 mg, 0.31 mmol) and MX_2 (0.31 mmol, MX_2 = $ZnCl_2$, $CdCl_2$, $HgBr_2$) in toluene (10 mL) and THF (1 mL) was stirred for 16 h at room temperature. After this time, the solvent was removed under vacuum to yield a white solid, which was dried at 60 °C under vacuum for 4 h, then extracted into toluene (2 x 15 mL). Concentration of the resultant colorless solution under vacuum, followed by storage at –30 °C, resulted in clear, colorless crystals of **7**–**9** with isolated yields of 59.2 mg (27%), 79.3 mg (34%) and 70.8 mg (28%) respectively.

Data for **7**: Elemental analysis: (Found: C, 74.7; H, 5.9. Calc. for $C_{44}H_{40}Cl_2Zn$: C, 75.0; H, 5.7%); ^{1}H NMR δ_H (400 MHz; C_6D_6): 6.99 (4H, t, *J* 7.6 Hz, 4 x H-8), 6.90 (8H, d, *J* 7.6 Hz, 8 x H-7), 6.78 (4H, s, 4 x H-3), 1.61 (24H, s, 24 x H-9); ^{13}C NMR δ_C (101 MHz; C_6D_6): 151.6 (C-2), 150.2 (C-1), 144.3 (C-5), 136.0 (C-6), 135.4 (C-4), 128.2 (C-7), 127.7 (C-8), 125.7 (C-3), 21.6 (C-9); HRMS (EI), m/z: (Found: 702.1802. Calc. for $C_{44}H_{40}Cl_2Zn$: 702.1793.)

Data for **8**: Elemental analysis: (Found: C, 70.05; H, 5.35. Calc. for $C_{44}H_{40}Cl_2Cd$: C, 70.3; H, 5.4%); ^{1}H NMR δ_H (400 MHz; C_6D_6): 6.98 (4H, t, *J* 7.5 Hz, 4 x H-8), 6.89 (8H, d, *J* 7.5 Hz, 8 x H-7), 6.88 (4H, s, 4 x H-3), 1.63 (24H, s, 24 x H-9); ^{13}C NMR δ_C (101 MHz; C_6D_6): 160.1 (C-1), 151.1 (C-2), 145.3 (C-5), 135.8 (C-6), 134.8 (C-4), 128.1 (C-7), 127.5 (C-8), 125.4 (C-3), 21.2 (C-9); ^{113}Cd NMR

δ_{Cd} (88.8 MHz; C_6D_6): –246.03 (m); HRMS (EI), m/z: (Found: 752.1546. Calc. for $C_{44}H_{40}Cl_2Cd$: 752.1535.)

Data for **9**: ^{1}H NMR δ_H (400 MHz; C_6D_6): 7.00 (4H, t, *J* 7.4 Hz, 4 x H-8), 6.92 (4H, s, 4 x H-3), 6.91 (8H, d, *J* 7.4 Hz, 8 x H-7), 1.61 (24H, s, 24 x H-9); ^{13}C NMR δ_C (101 MHz; C_6D_6): 170.2 (C-1), 150.1 (C-2), 143.4 (C-5), 136.1 (C-6), 134.7 (C-4), 127.9 (C-7), 127.6 (C-8), 127.0 (C-3), 21.2 (C-9); ^{199}Hg NMR δ_{Hg} (71.7 MHz; C_6D_6): –695.04 (s); HRMS (FD), m/z: (Found: 840.2249. Calc. for $C_{44}H_{40}Cl_2Hg$: 840.2208.)

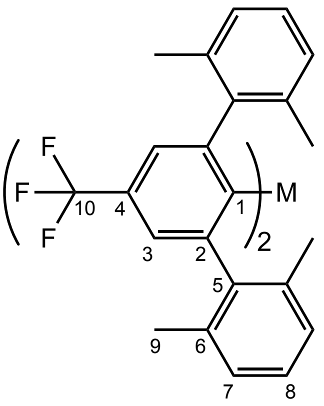

S1.2.4. Synthesis of (F_3C-Ar$^{\#}$)$_2$M (M = Zn 10, Cd 11, Hg 12)

A mixture of [F_3C-Ar$^{\#}$-Li]$_2$ (100 mg, 0.14 mmol) and MX_2 (0.14 mmol, MX_2 = $ZnCl_2$, $CdCl_2$, $HgBr_2$) in toluene (10 mL) and THF (1 mL) was stirred for 16 h at room temperature. After this time, the solvent was removed under vacuum to yield a white solid, which was dried at 60 °C under vacuum for 4 h, then extracted into hexane (3 x 10 mL). Concentration of the resultant colorless solution under vacuum, followed by storage at –30 °C, resulted in clear, colorless crystals of **10**–**12** with isolated yields of 43.7 mg (41%), 48.0 mg (42%) and 56.0 mg (44%) respectively.

Data for **10**: Elemental analysis: (Found: C, 71.3; H, 5.3. Calc. for $C_{46}H_{40}F_6Zn$: C, 71.6; H, 5.2%); ^{1}H NMR δ_H (400 MHz; C_6D_6): 7.05 (4H, s, 4 x H-3), 6.99 (4H, t, *J* 7.5 Hz, 4 x H-8), 6.89 (8H, d, *J* 7.5 Hz, 8 x H-7), 1.55 (24H, s, 24 x H-9); ^{13}C NMR δ_C (101 MHz; C_6D_6): 156.8 (C-1), 150.7 (C-2), 144.2 (C-5), 136.0 (C-6), 131.5 (q, *J* 31.7 Hz, C-4), 128.3 (C-7), 127.9 (C-8), 125.1 (q, *J* 272.4 Hz, C-10), 122.1 (q, *J* 3.5 Hz, C-3), 21.6 (C-9); ^{19}F NMR δ_F (376 MHz; C_6D_6): –62.05 (s); HRMS (FD), m/z: (Found: 770.2294. Calc. for $C_{46}H_{40}F_6Zn$: 770.2320.)

Data for **11**: Elemental analysis: (Found: C, 67.55; H, 5.05. Calc. for $C_{46}H_{40}F_6Cd$: C, 67.4; H, 4.9%); ^{1}H NMR δ_H (400 MHz; C_6D_6): 7.14 (4H, s, 4 x H-3), 6.98 (4H, t, *J* 7.5 Hz, 4 x H-8), 6.88 (8H, d,

J 7.5 Hz, 8 x H-7), 1.56 (24H, s, 24 x H-9); ^{13}C NMR δ_C (101 MHz; C_6D_6): 167.0 (C-1), 150.2 (C-2), 145.3 (C-5), 135.8 (C-6), 131.1 (q, *J* 31.5 Hz, C-4), 128.2 (C-7), 127.7 (C-8), 125.3 (q, *J* 272.3 Hz, C-10), 121.7 (q, *J* 3.4 Hz, C-3), 21.3 (C-9); ^{19}F NMR δ_F (376 MHz; C_6D_6): –61.82 (s); ^{113}Cd NMR δ_{Cd} (88.8 MHz; C_6D_6): –265.21 (m); HRMS (FD), m/z: (Found: 820.2092. Calc. for $C_{46}H_{40}F_6Cd$: 820.2062.)

Data for **12**: ^{1}H NMR δ_H (400 MHz; C_6D_6): 7.20 (4H, s, 4 x H-3), 7.00 (4H, t, *J* 7.4 Hz, 4 x H-8), 6.89 (8H, d, *J* 7.4 Hz, 8 x H-7), 1.54 (24H, s, 24 x H-9); ^{13}C NMR δ_C (101 MHz; C_6D_6): 176.0 (C-1), 149.3 (C-2), 143.2 (C-5), 136.1 (C-6), 131.1 (q, *J* 31.8 Hz, C-4), 128.0 (C-7), 127.9 (C-8), 125.1 (q, *J* 272.5 Hz, C-10), 123.6 (q, *J* 3.5 Hz, C-3), 21.2 (C-9); ^{19}F NMR δ_F (376 MHz; C_6D_6): –61.88 (s); ^{199}Hg NMR δ_{Hg} (71.7 MHz; C_6D_6): –745.00 (s); HRMS (FD), m/z: (Found: 908.2776. Calc. for $C_{46}H_{40}F_6Hg$: 908.2735.)

S2. Supplementary NMR Data

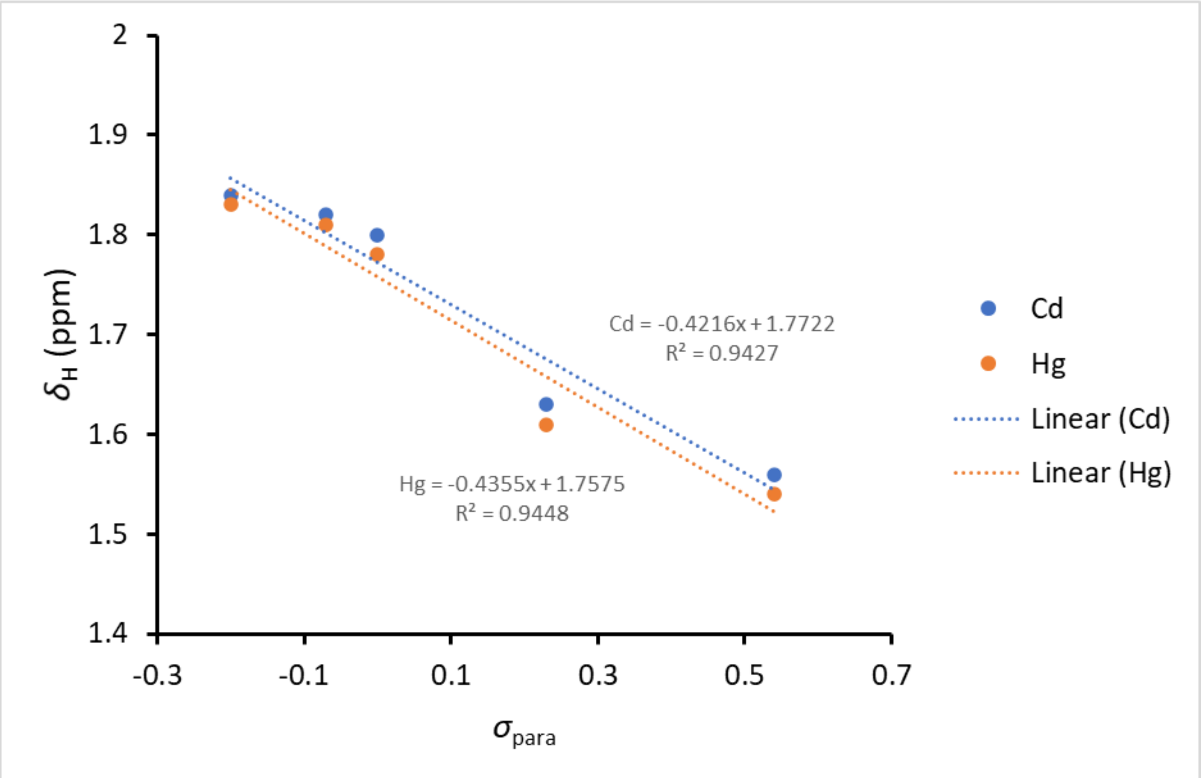

Figure S1: Plot of the ^{1}H NMR chemical shifts for the flanking methyl protons, H-9, of the metal diaryls (R-Ar$^{\#}$)$_2$M (M = Cd, Hg; R = *t*-Bu, $SiMe_3$, H, Cl, CF_3) versus the Hammet constant, σ_{para}, of their corresponding R group.

S2.1. (t-Bu-Ar$^{\#}$)$_2$Zn (1)

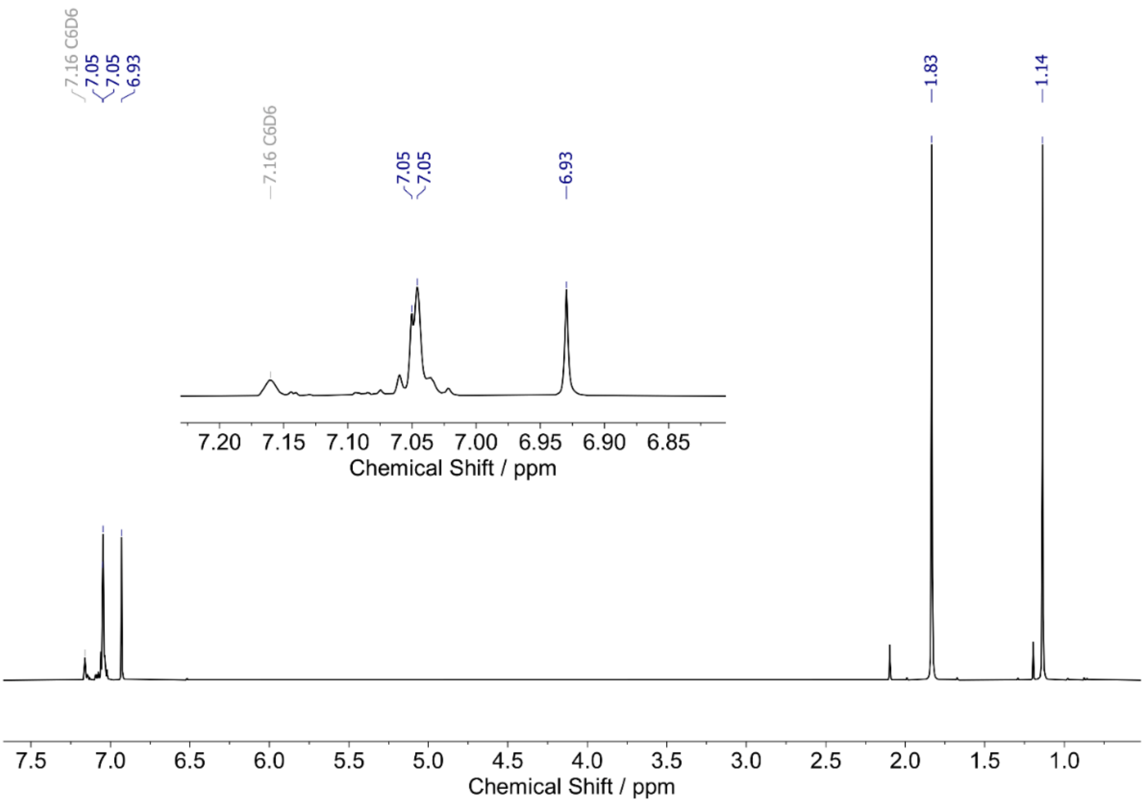

Figure S2: ^{1}H NMR (400 MHz, 25 °C) spectrum for **1** in C_6D_6.

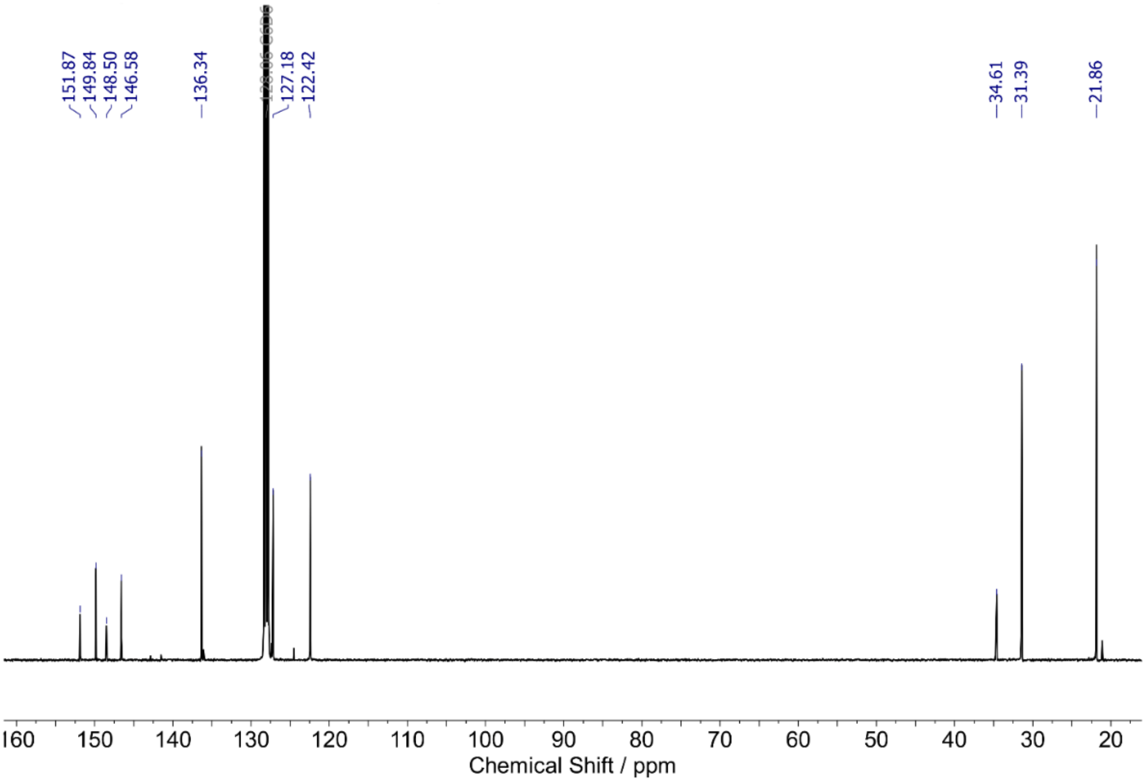

Figure S3: $^{13}C\{^{1}H\}$ NMR (101 MHz, 25 °C) spectrum for **1** in C_6D_6.

S2.2. (*t*-Bu-Ar#)$_2$Cd (2)

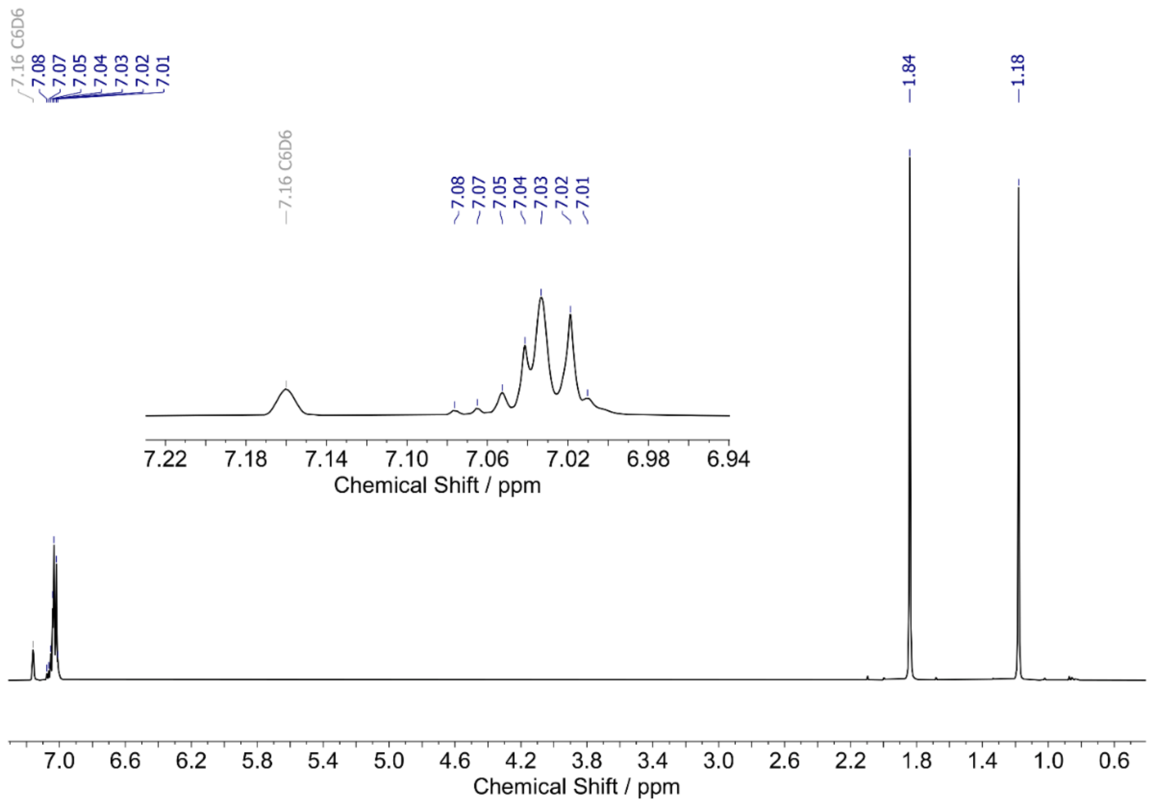

Figure S4: ^{1}H NMR (400 MHz, 25 °C) spectrum for **2** in C_6D_6.

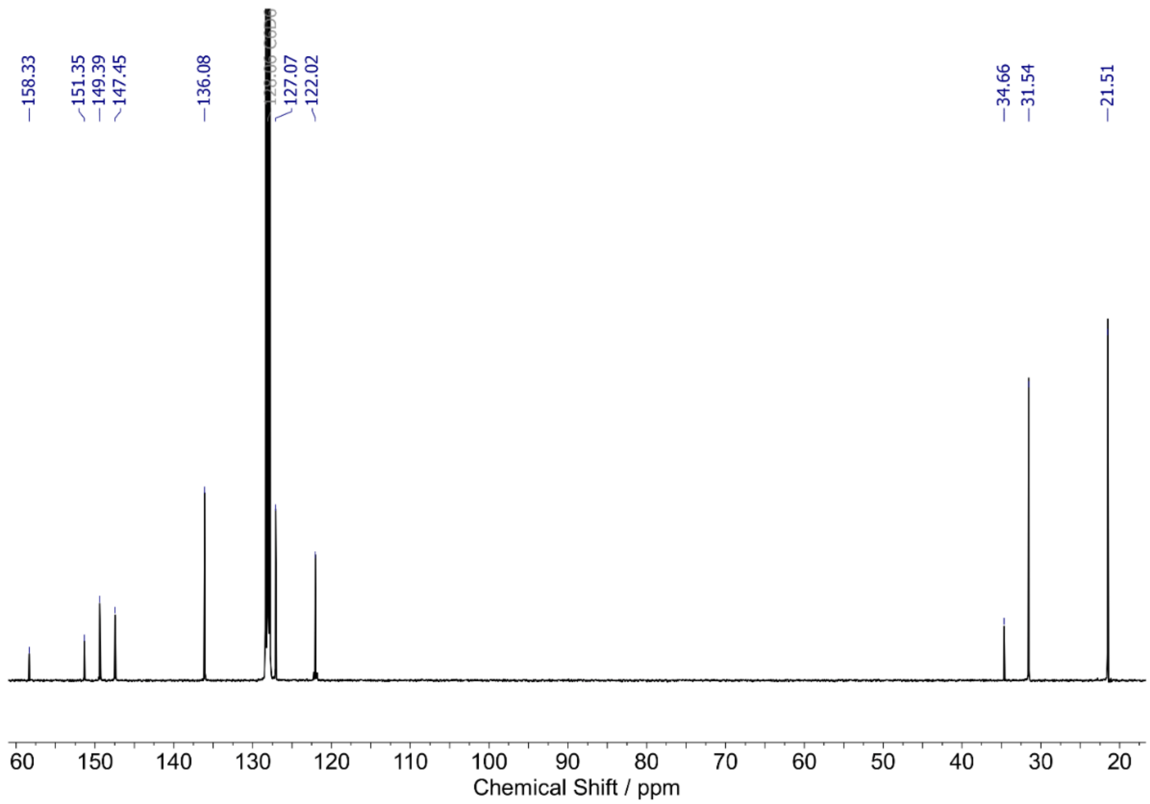

Figure S5: $^{13}C\{^{1}H\}$ NMR (101 MHz, 25 °C) spectrum for **2** in C_6D_6.

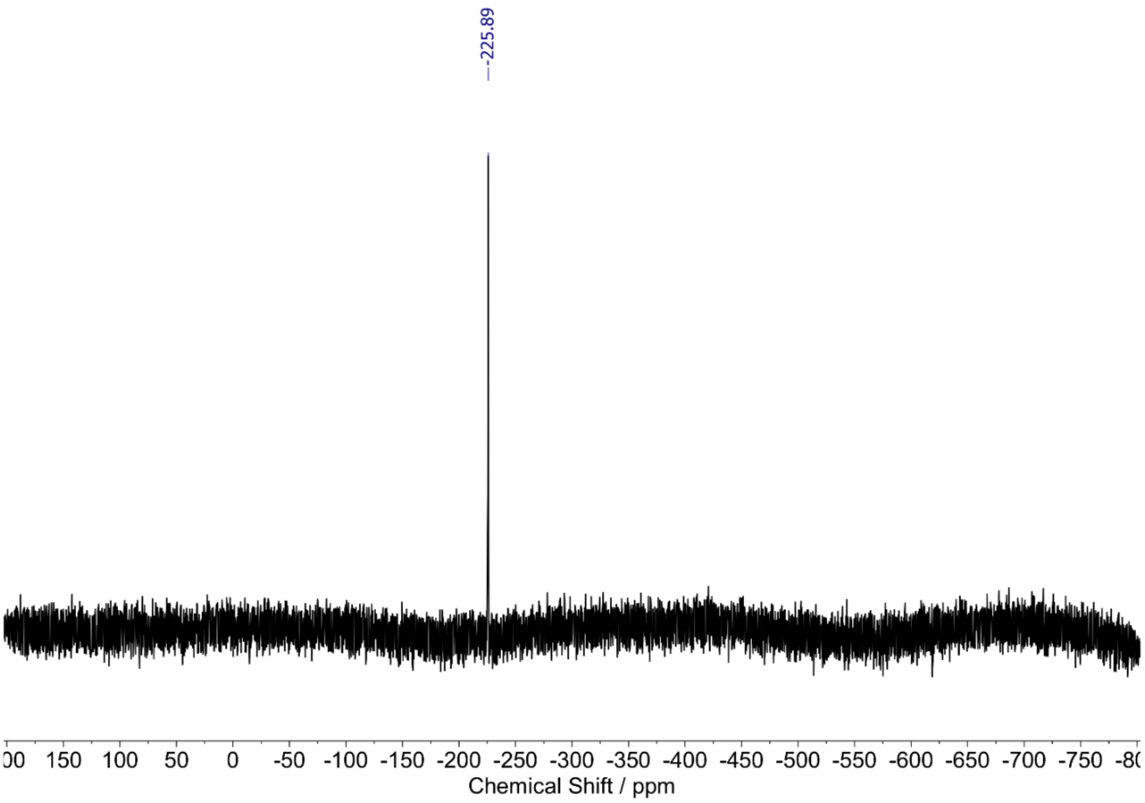

Figure S6: ^{113}Cd NMR (89 MHz, 25 °C) spectrum for **2** in C_6D_6.

S2.3. (*t*-Bu-Ar[#])$_2$Hg (3)

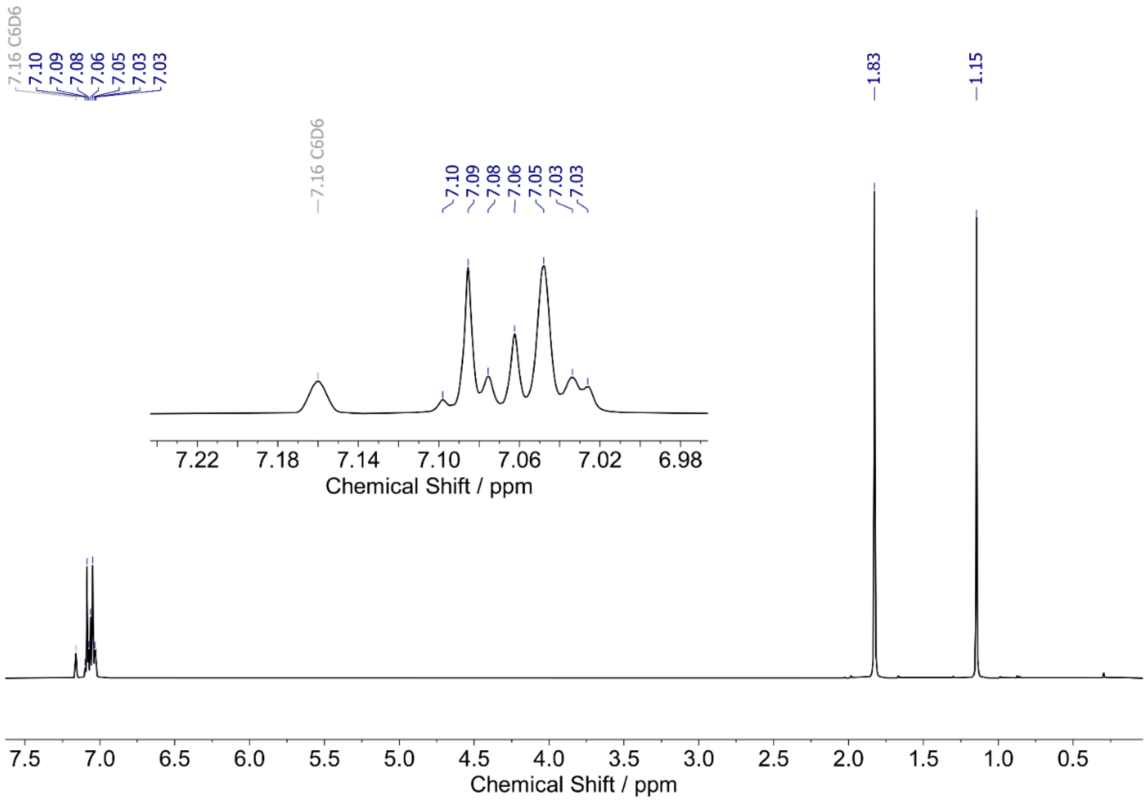

Figure S7: ^{1}H NMR (400 MHz, 25 °C) spectrum for **3** in C_6D_6.

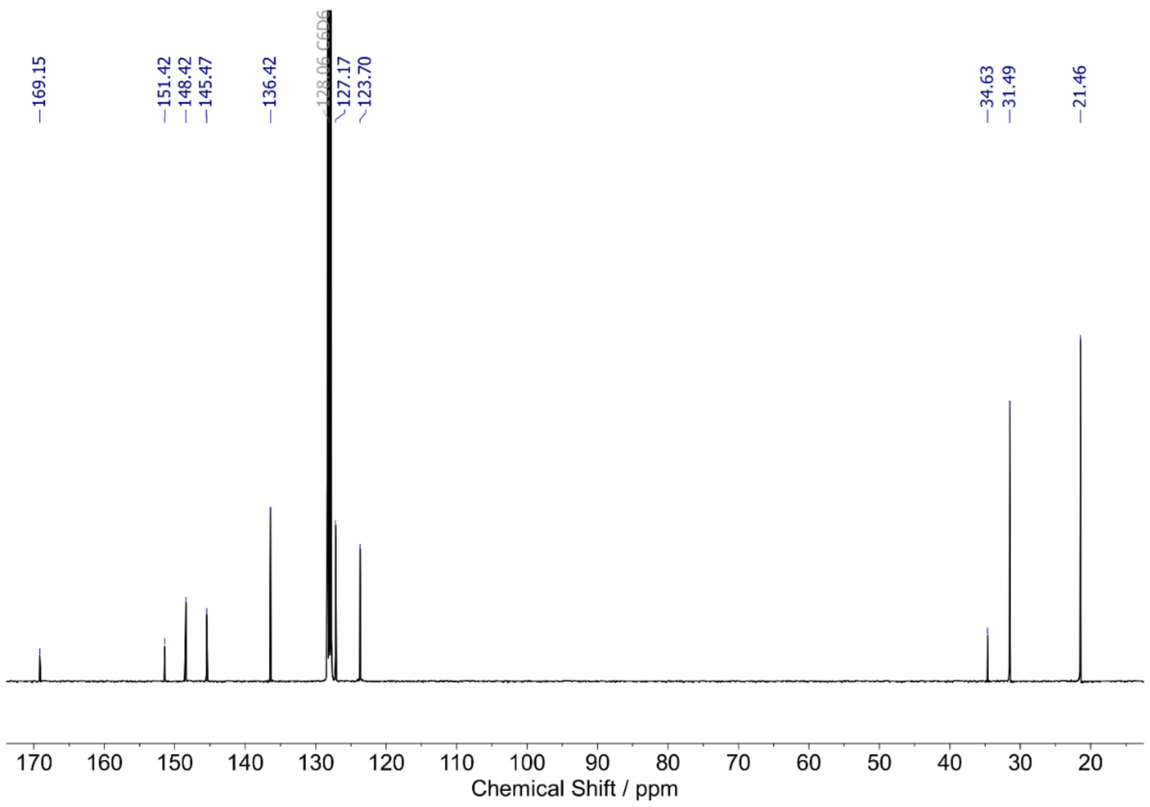

Figure S8: $^{13}C\{^{1}H\}$ NMR (101 MHz, 25 °C) spectrum for **3** in C_6D_6.

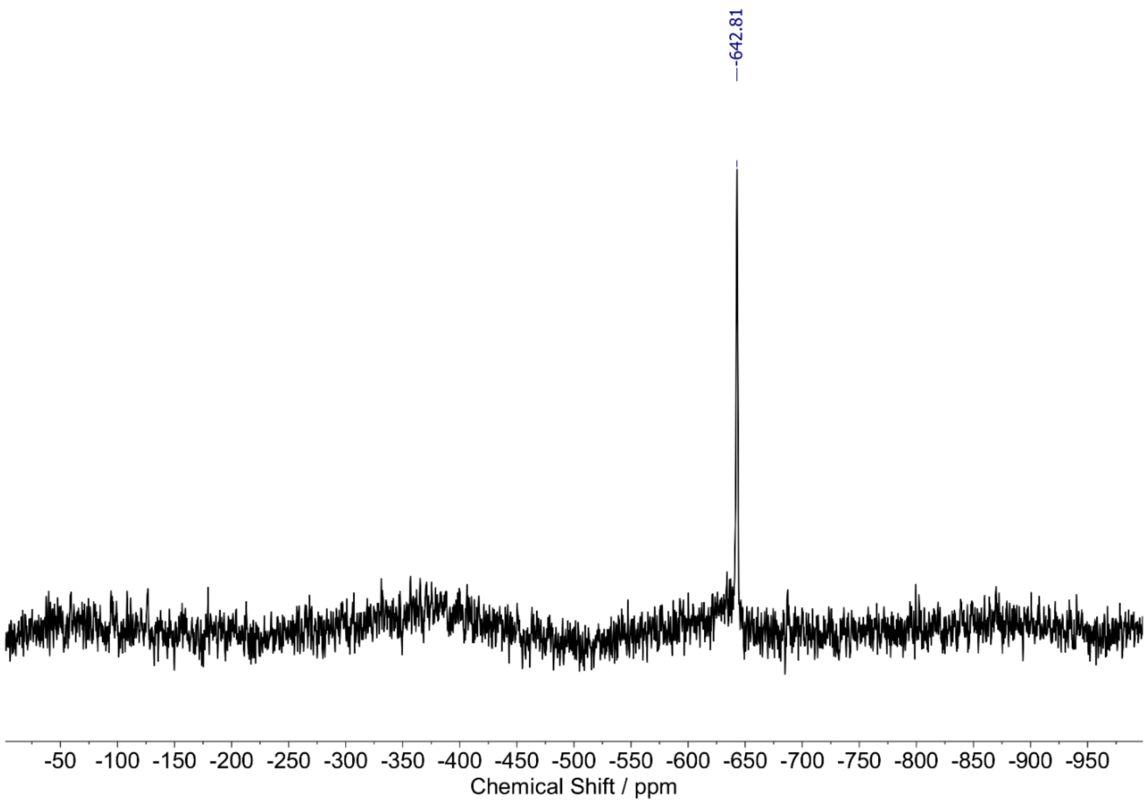

Figure S9: ^{199}Hg NMR (72 MHz, 25 °C) spectrum for **3** in C_6D_6.

S2.4. $(Me_3Si\text{-}Ar^{\#})_2Zn$ (4)

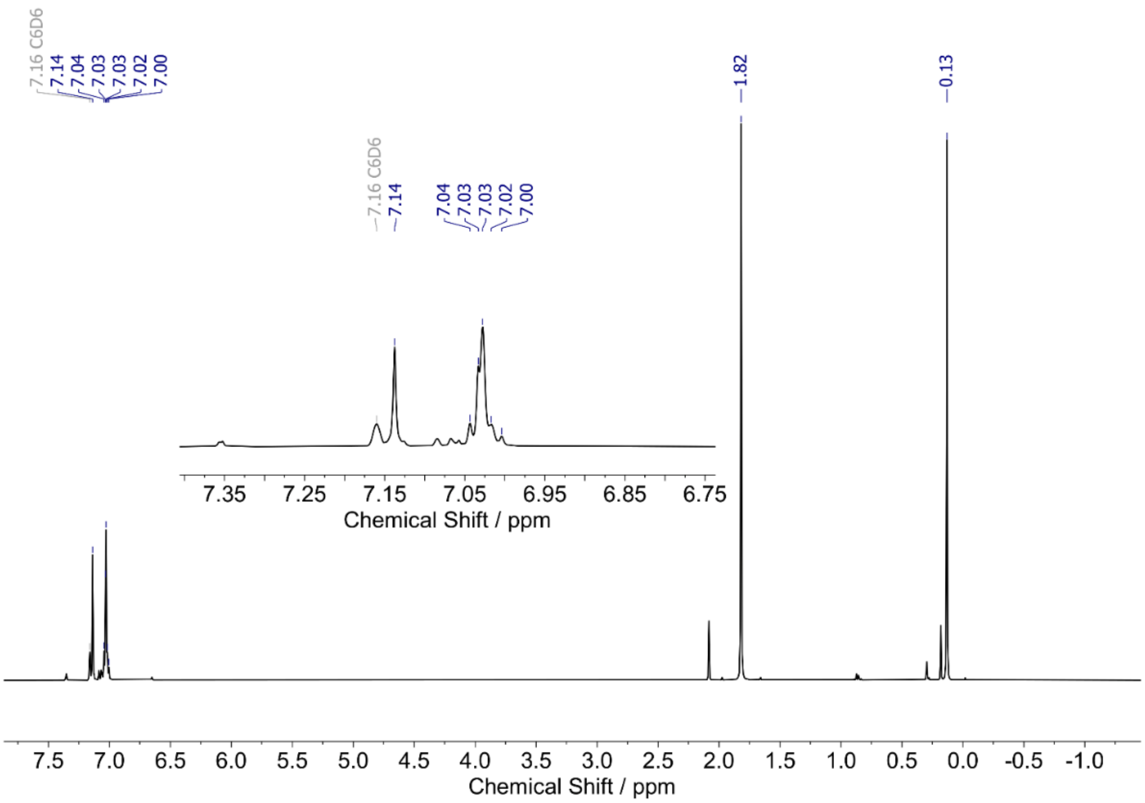

Figure S10: ^{1}H NMR (400 MHz, 25 °C) spectrum for **4** in C_6D_6.

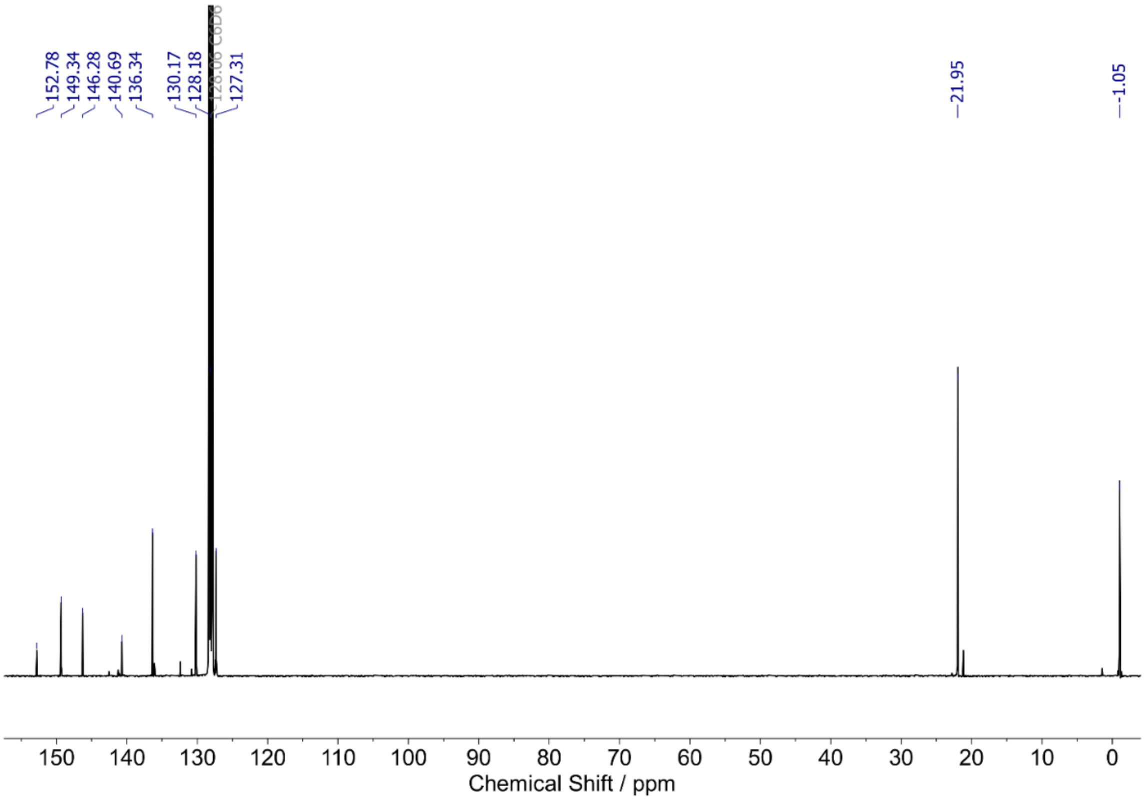

Figure S11: $^{13}C\{^{1}H\}$ NMR (101 MHz, 25 °C) spectrum for **4** in C_6D_6.

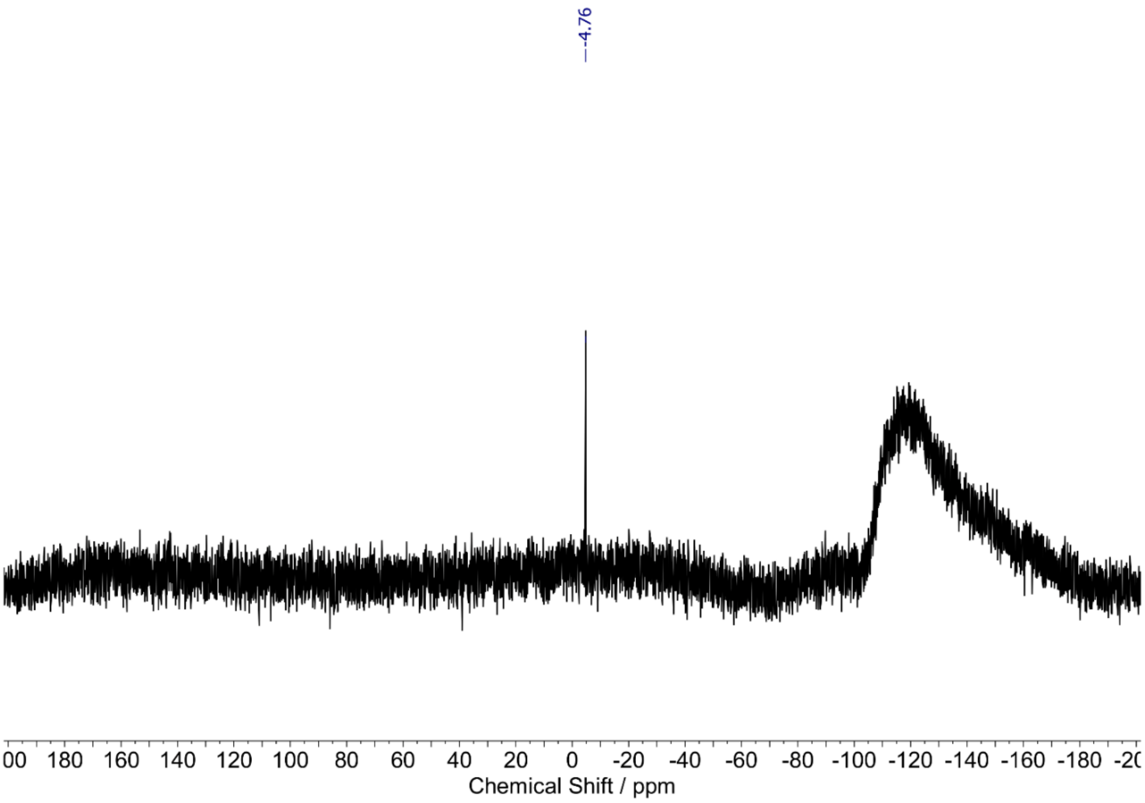

Figure S12: $^{29}Si\{^{1}H\}$ NMR (79 MHz, 25 °C) spectrum for **4** in C_6D_6.

S2.5. $(Me_3Si\text{-}Ar^{\#})_2Cd$ (5)

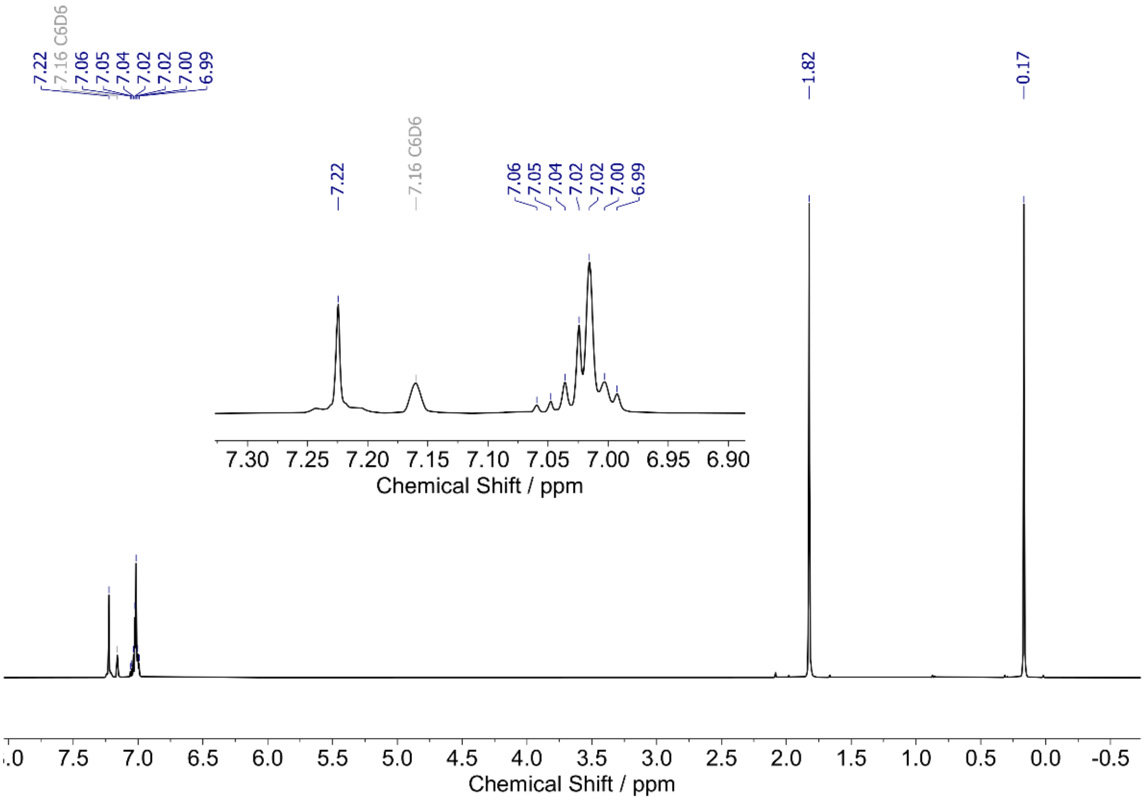

Figure S13: ^{1}H NMR (400 MHz, 25 °C) spectrum for **5** in C_6D_6.

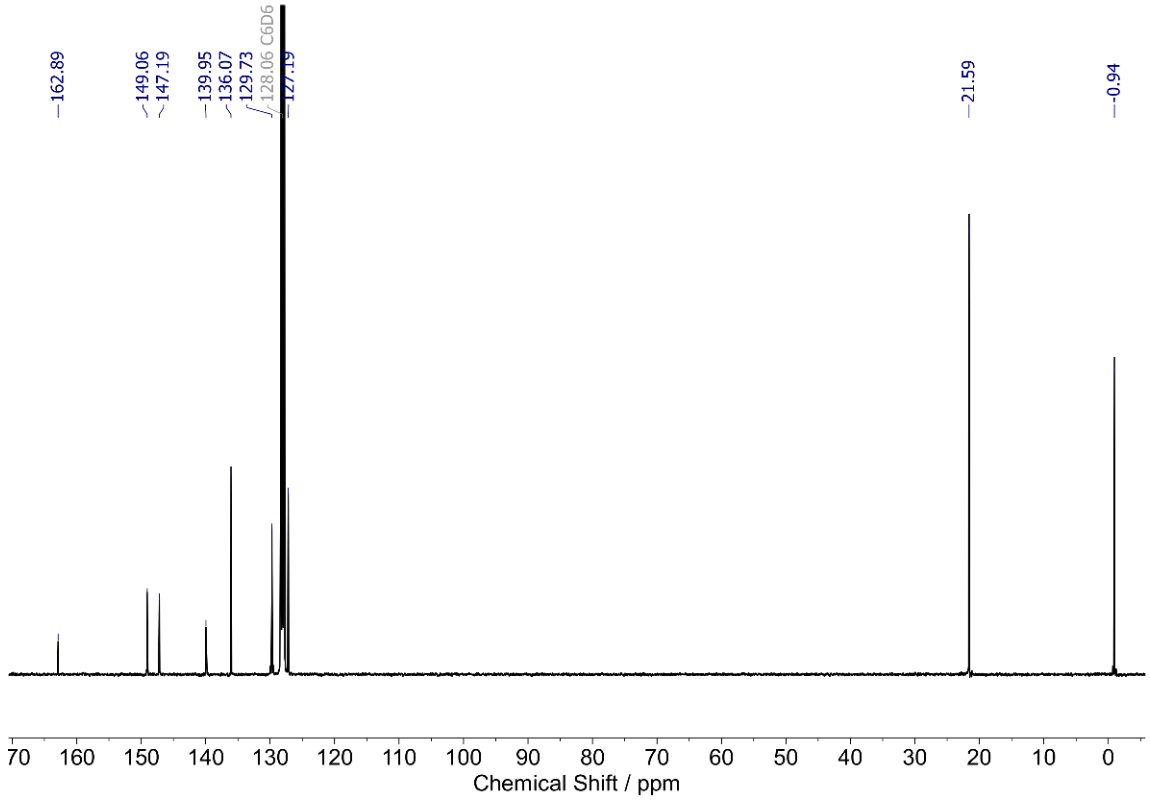

Figure S14: $^{13}C\{^{1}H\}$ NMR (101 MHz, 25 °C) spectrum for **5** in C_6D_6.

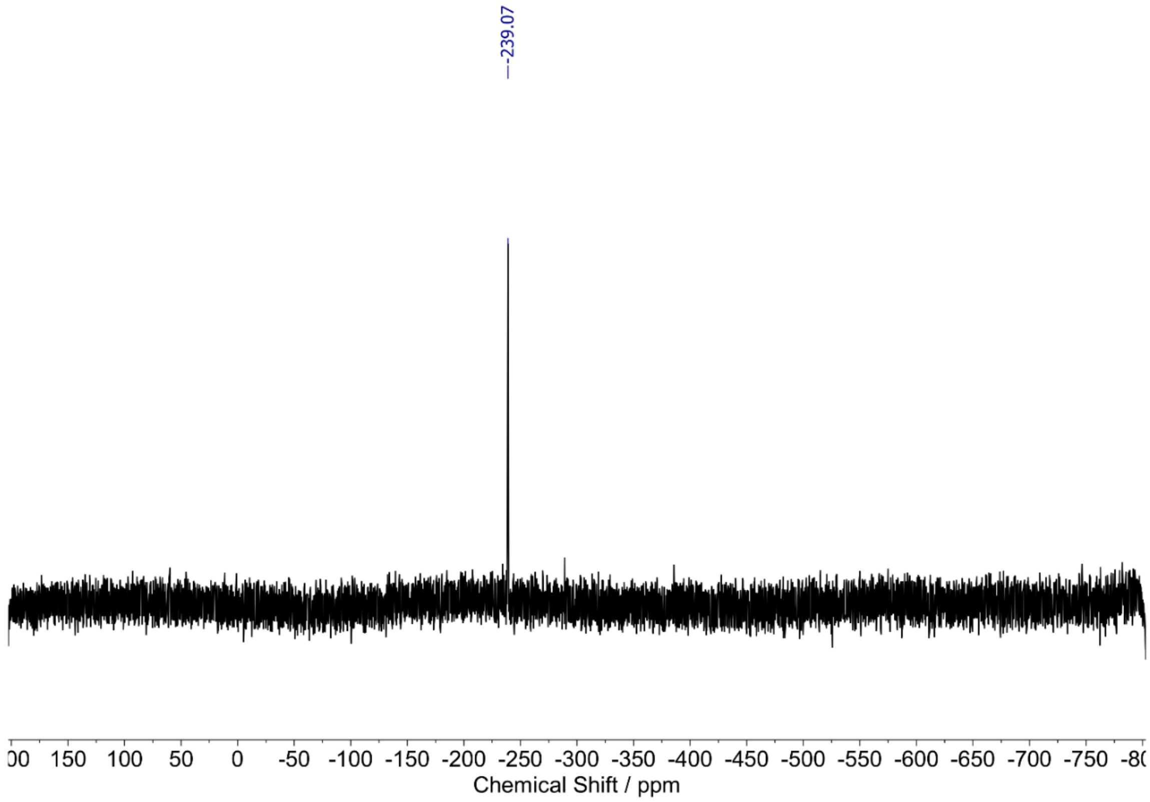

Figure S15: ^{113}Cd NMR (89 MHz, 25 °C) spectrum for **5** in C_6D_6.

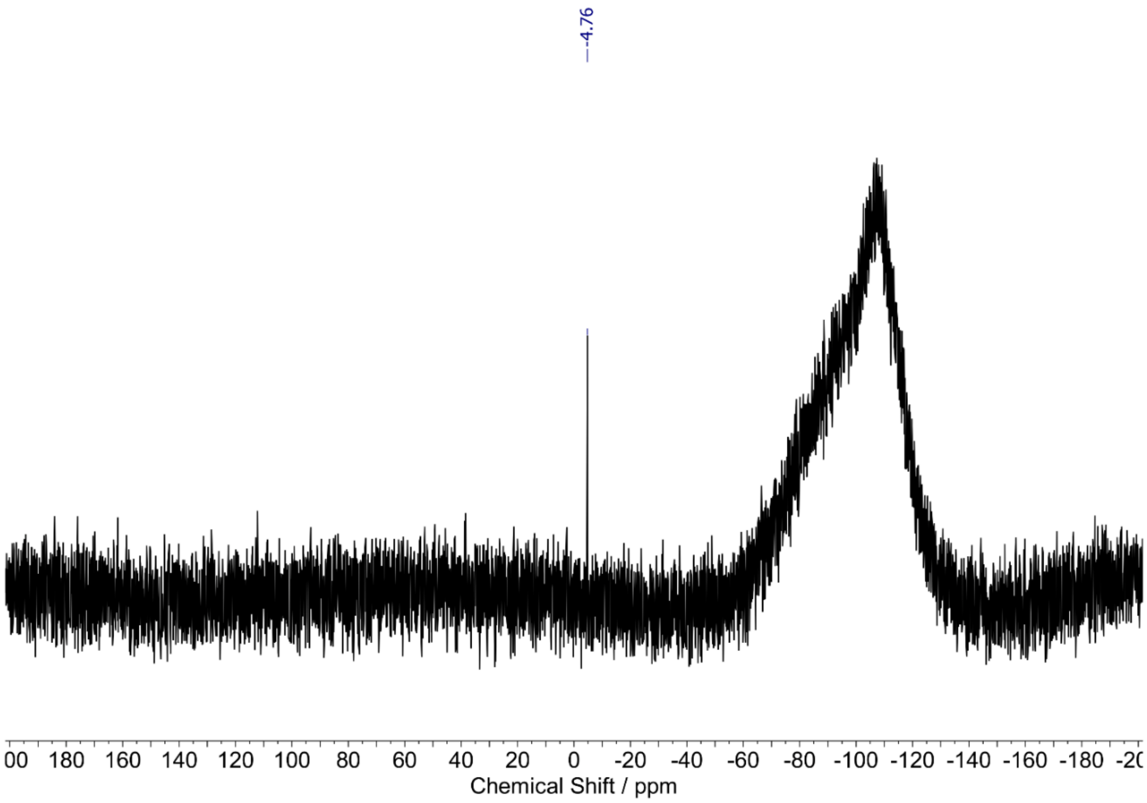

Figure S16: $^{29}Si\{^1H\}$ NMR (79 MHz, 25 °C) spectrum for **5** in C_6D_6.

S2.6. $(Me_3Si\text{-}Ar^{\#})_2Hg$ (6)

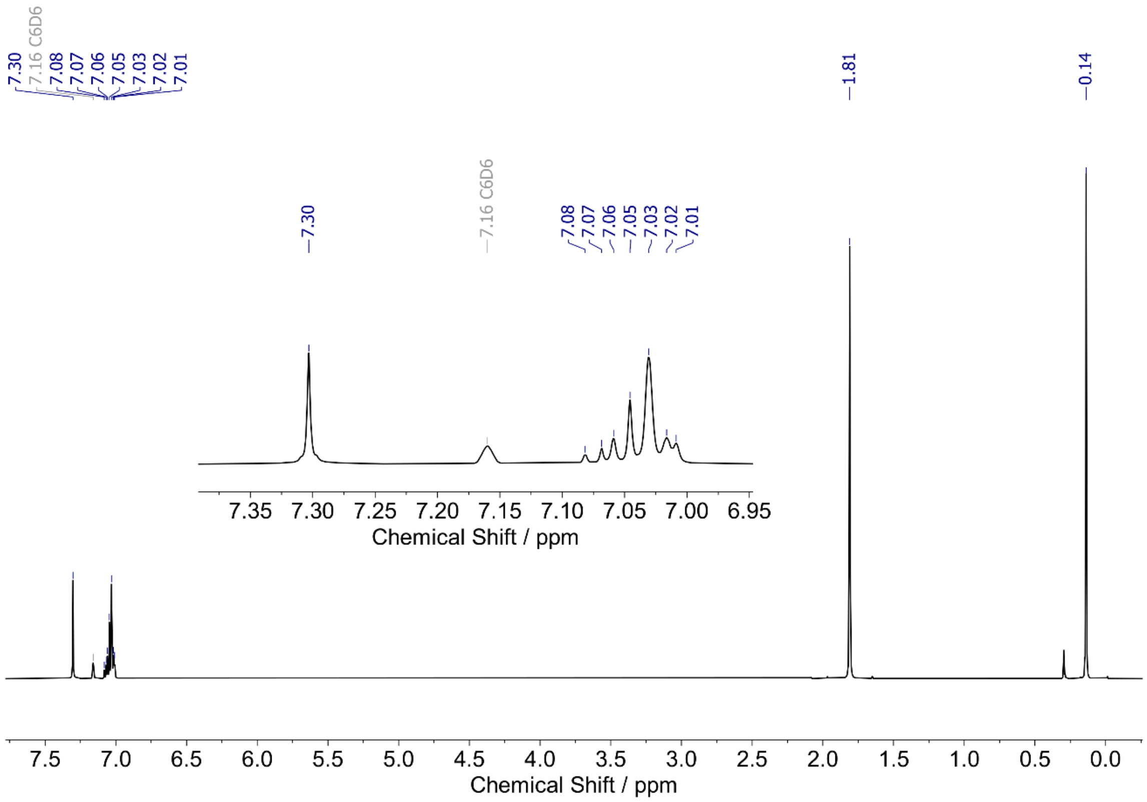

Figure S17: ^1H NMR (400 MHz, 25 °C) spectrum for **6** in C_6D_6.

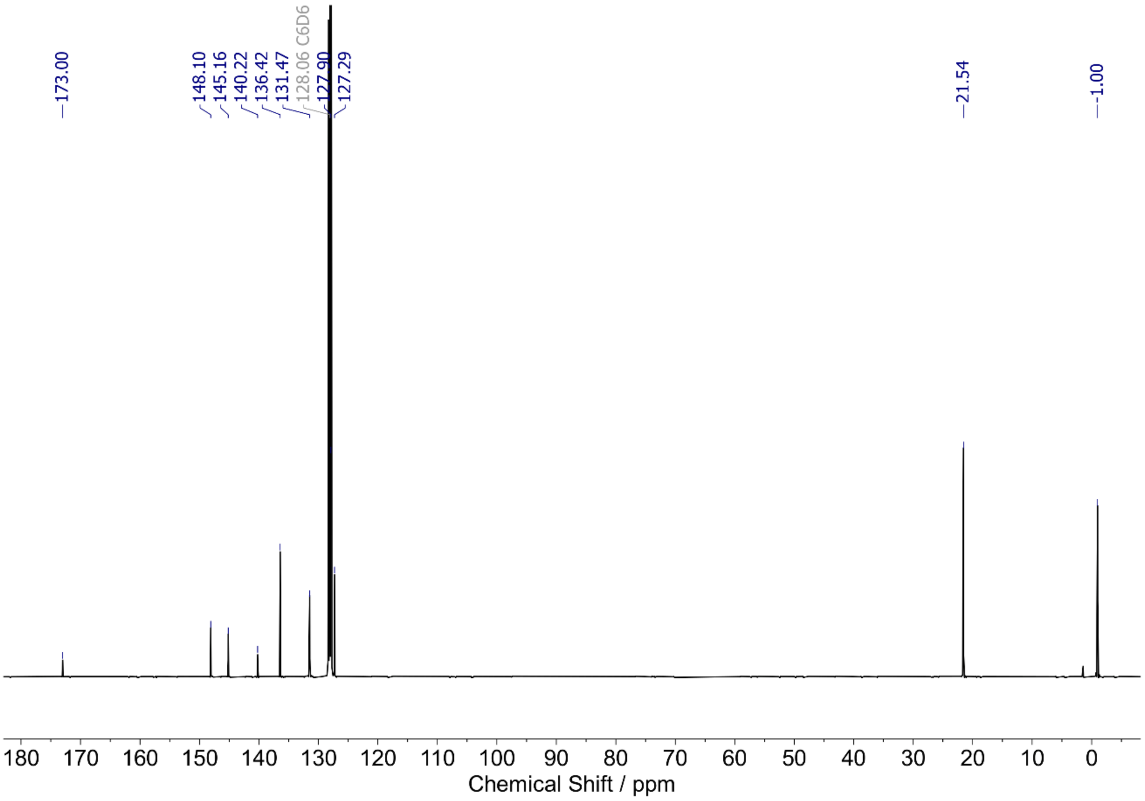

Figure S18: $^{13}C\{^{1}H\}$ NMR (101 MHz, 25 °C) spectrum for **6** in C_6D_6.

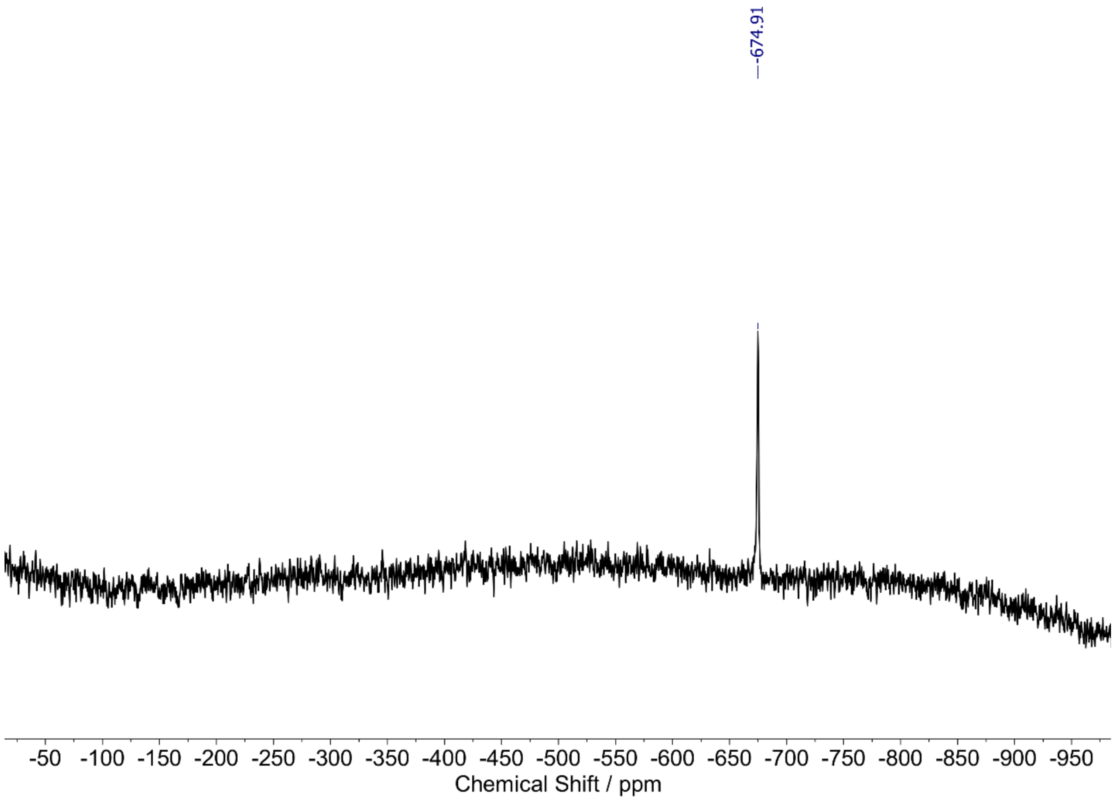

Figure S19: ^{199}Hg NMR (72 MHz, 25 °C) spectrum for **6** in C_6D_6.

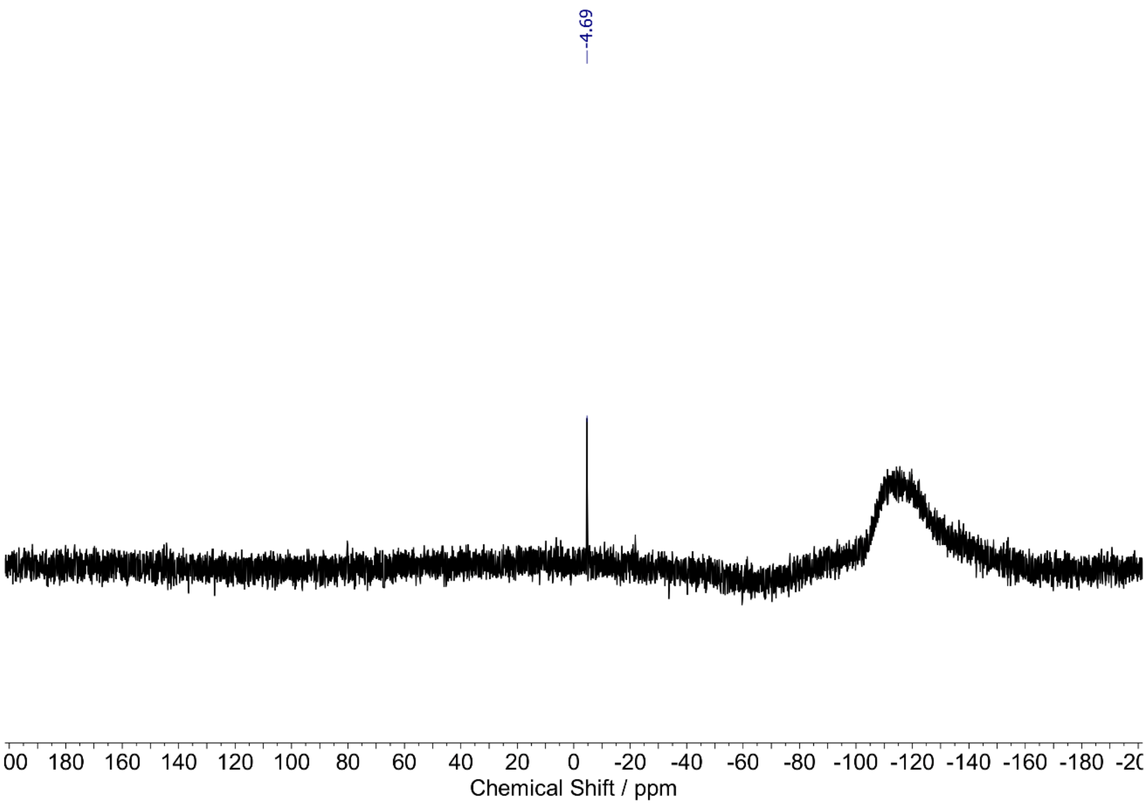

Figure S20: $^{29}Si\{^{1}H\}$ NMR (79 MHz, 25 °C) spectrum for **6** in C_6D_6.

S2.7. (Cl-Ar$^{\#}$)$_2$Zn (7)

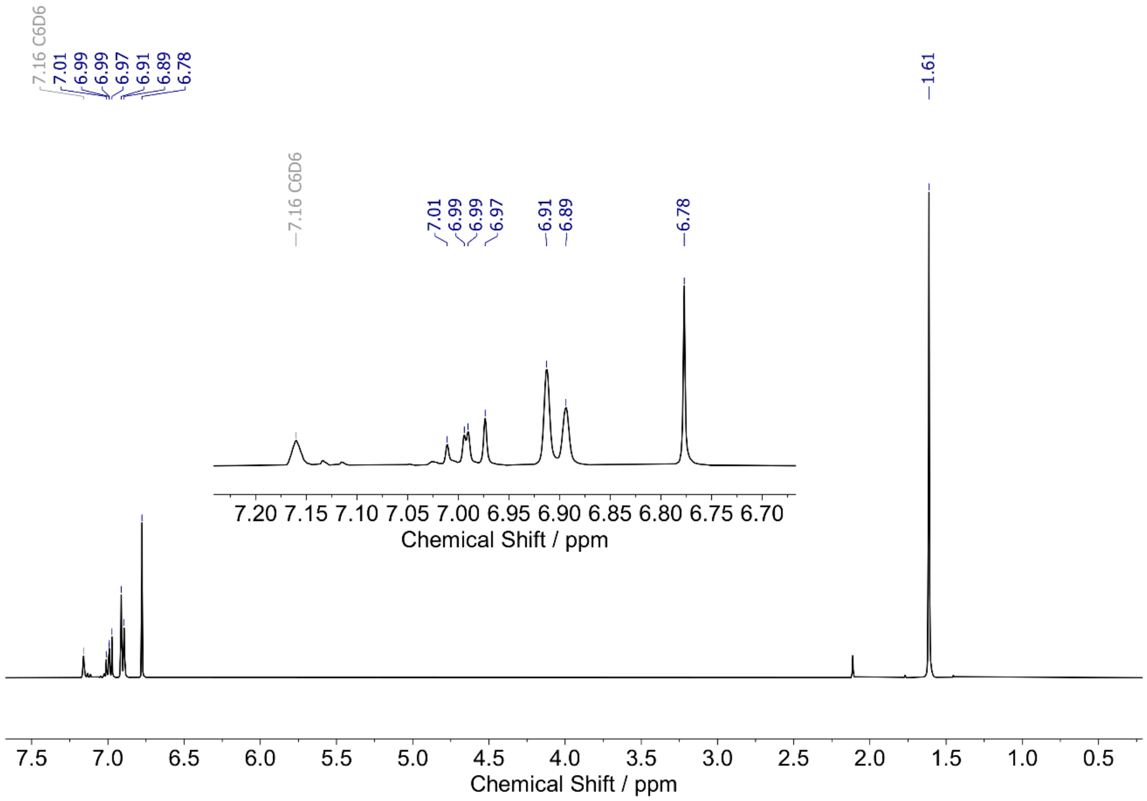

Figure S21: ^{1}H NMR (400 MHz, 25 °C) spectrum for **7** in C_6D_6.

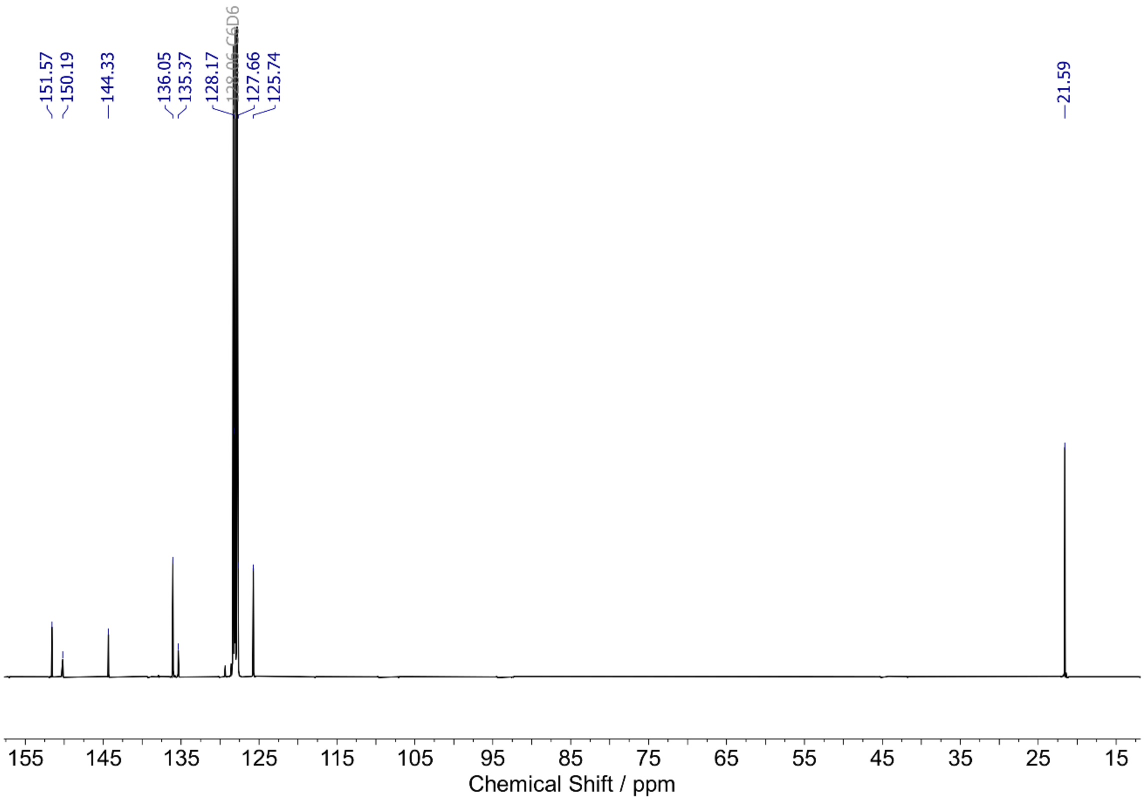

Figure S22: $^{13}C\{^{1}H\}$ NMR (101 MHz, 25 °C) spectrum for **7** in C_6D_6.

S2.8. (Cl-Ar[#])$_2$Cd (8)

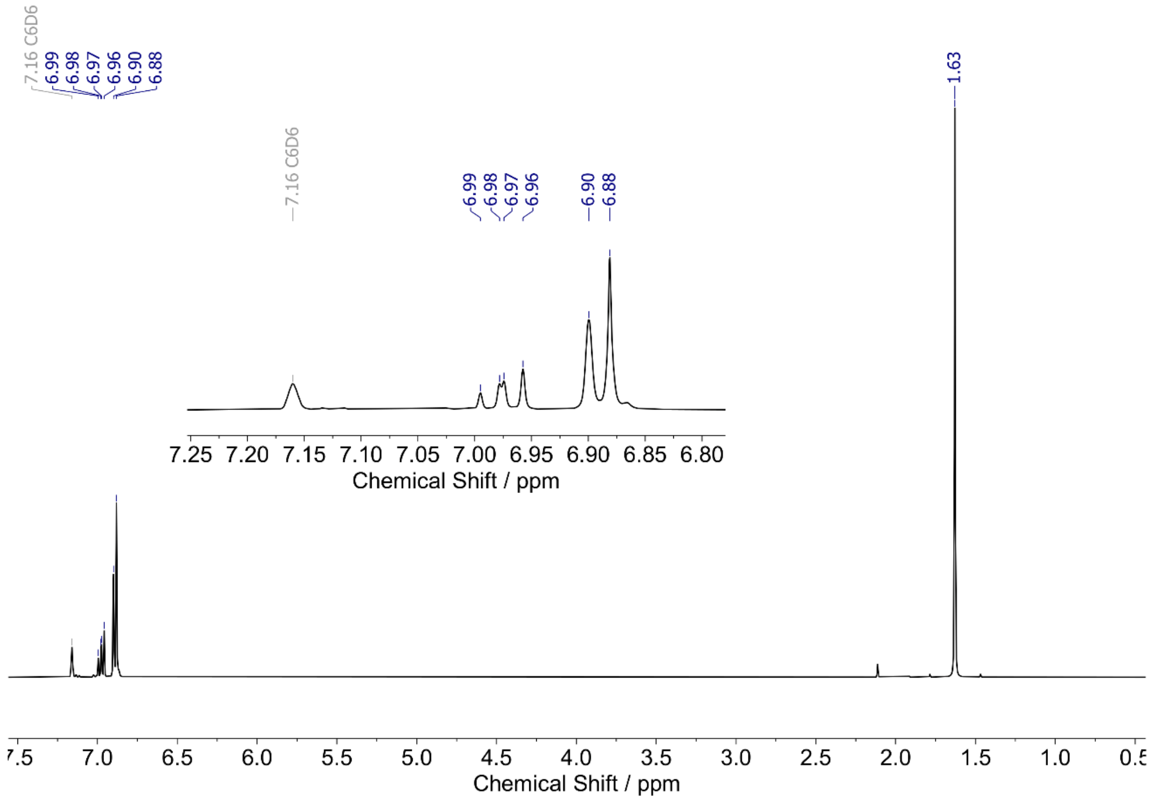

Figure S23: ^{1}H NMR (400 MHz, 25 °C) spectrum for **8** in C_6D_6.

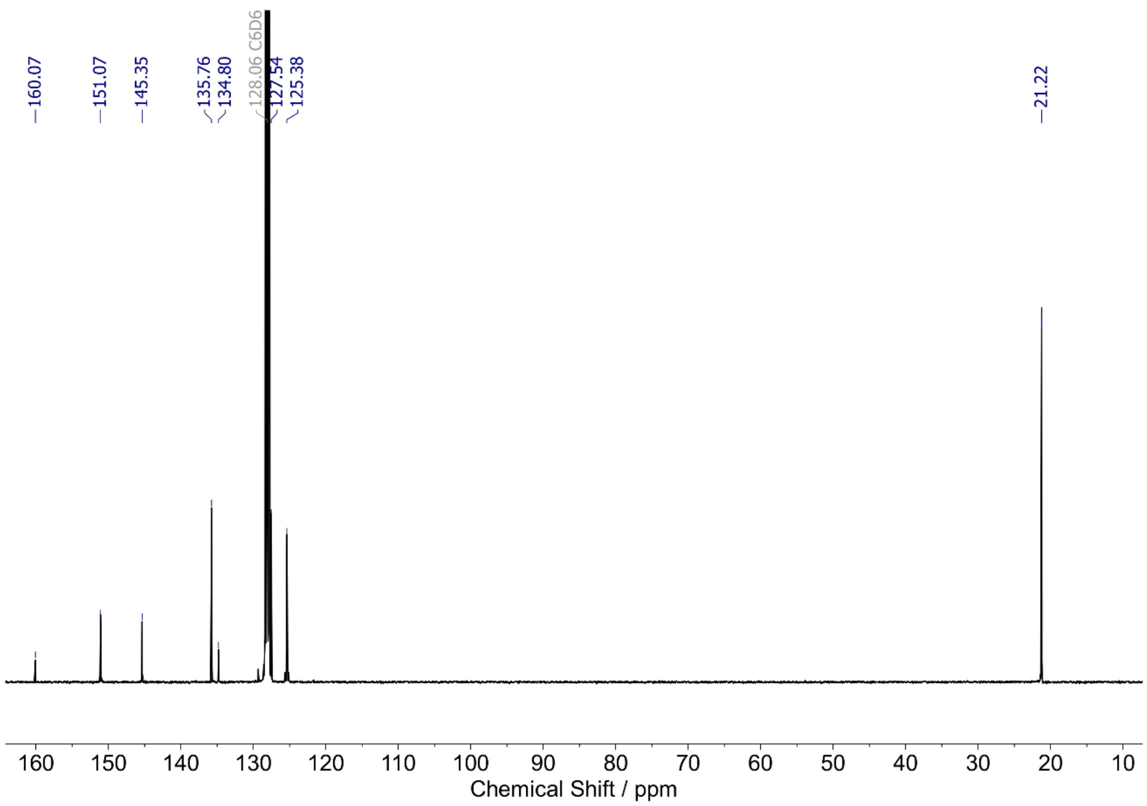

Figure S24: $^{13}C\{^{1}H\}$ NMR (101 MHz, 25 °C) spectrum for **8** in C_6D_6.

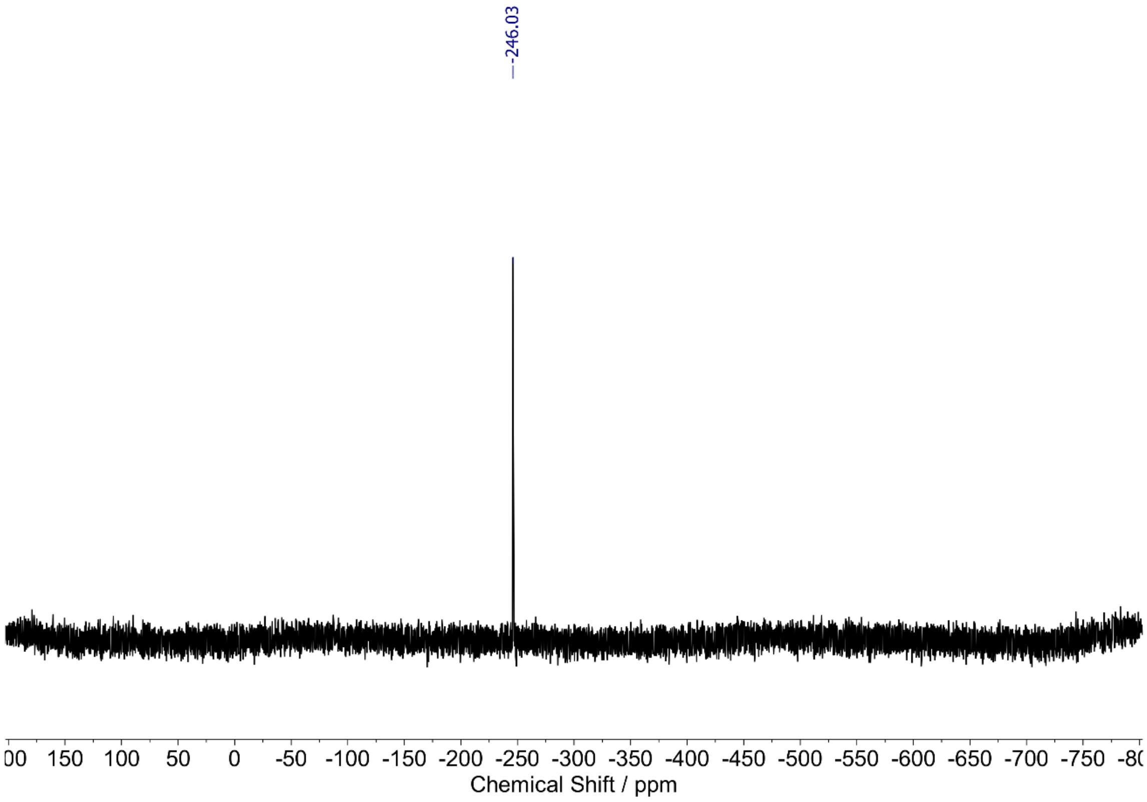

Figure S25: ^{113}Cd NMR (89 MHz, 25 °C) spectrum for **8** in C_6D_6.

S2.9. (Cl-Ar#)$_2$Hg (9)

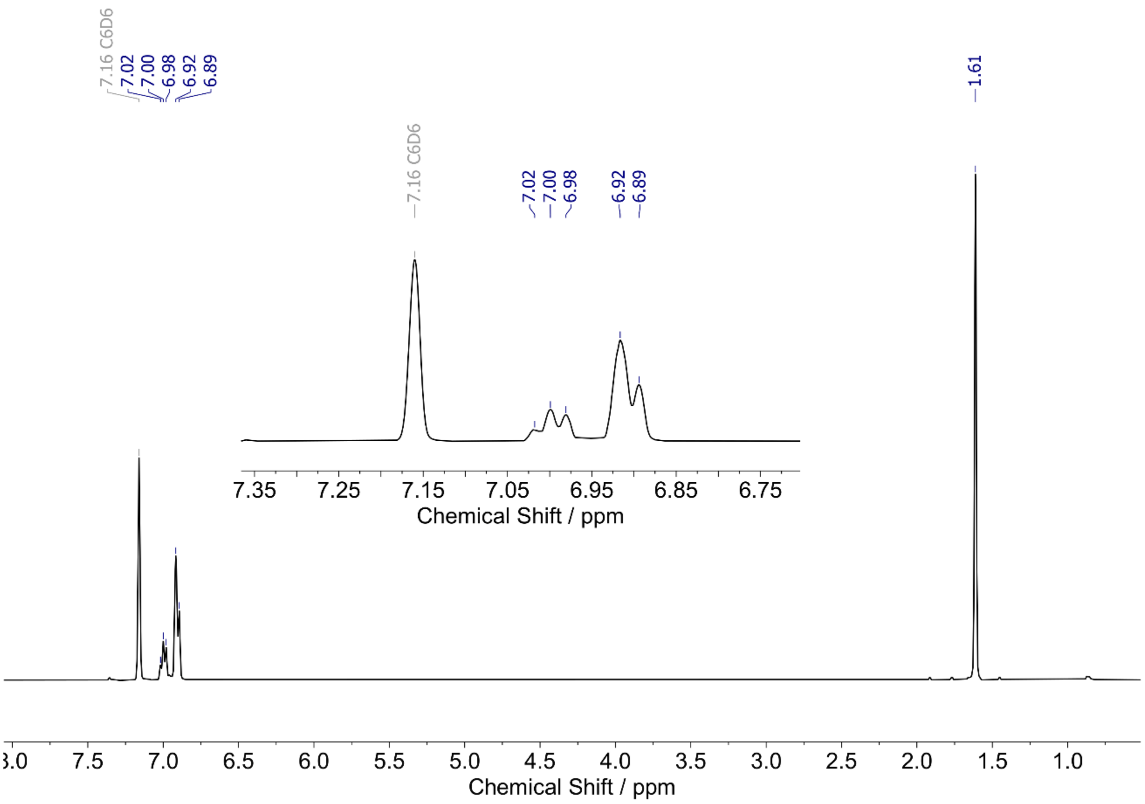

Figure S26: ^{1}H NMR (400 MHz, 25 °C) spectrum for **9** in C_6D_6.

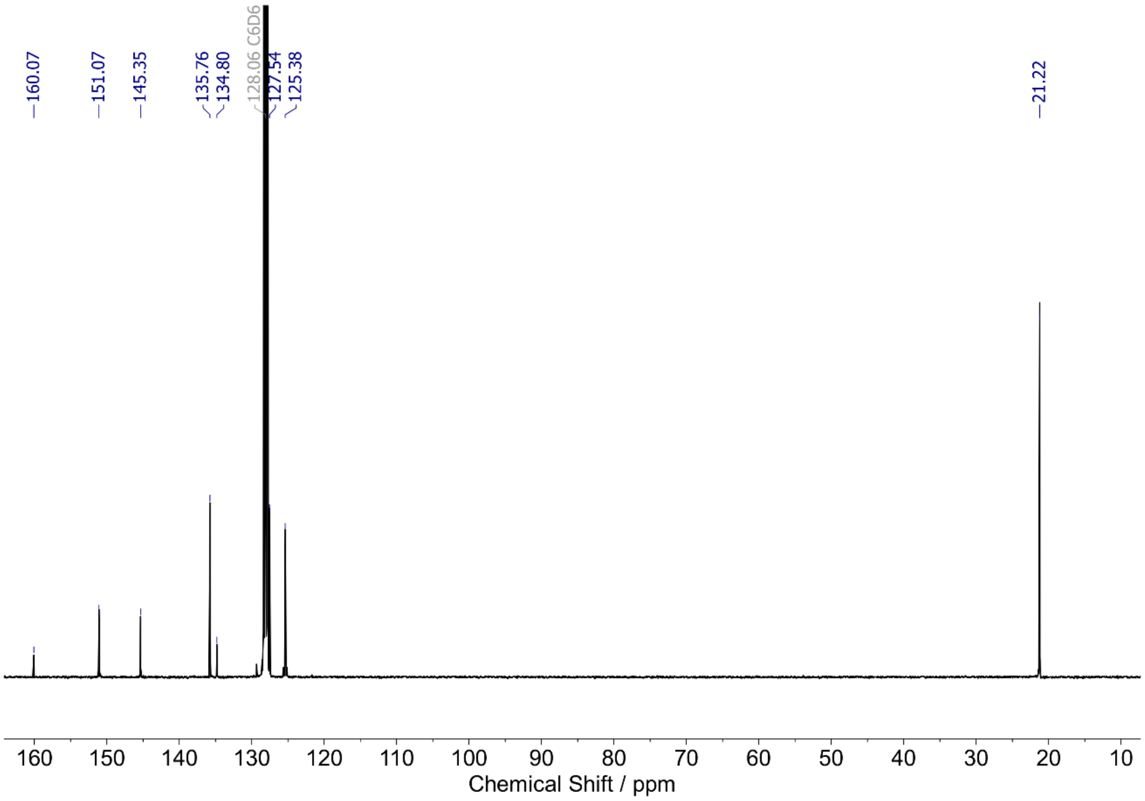

Figure S27: $^{13}C\{^1H\}$ NMR (101 MHz, 25 °C) spectrum for **9** in C_6D_6.

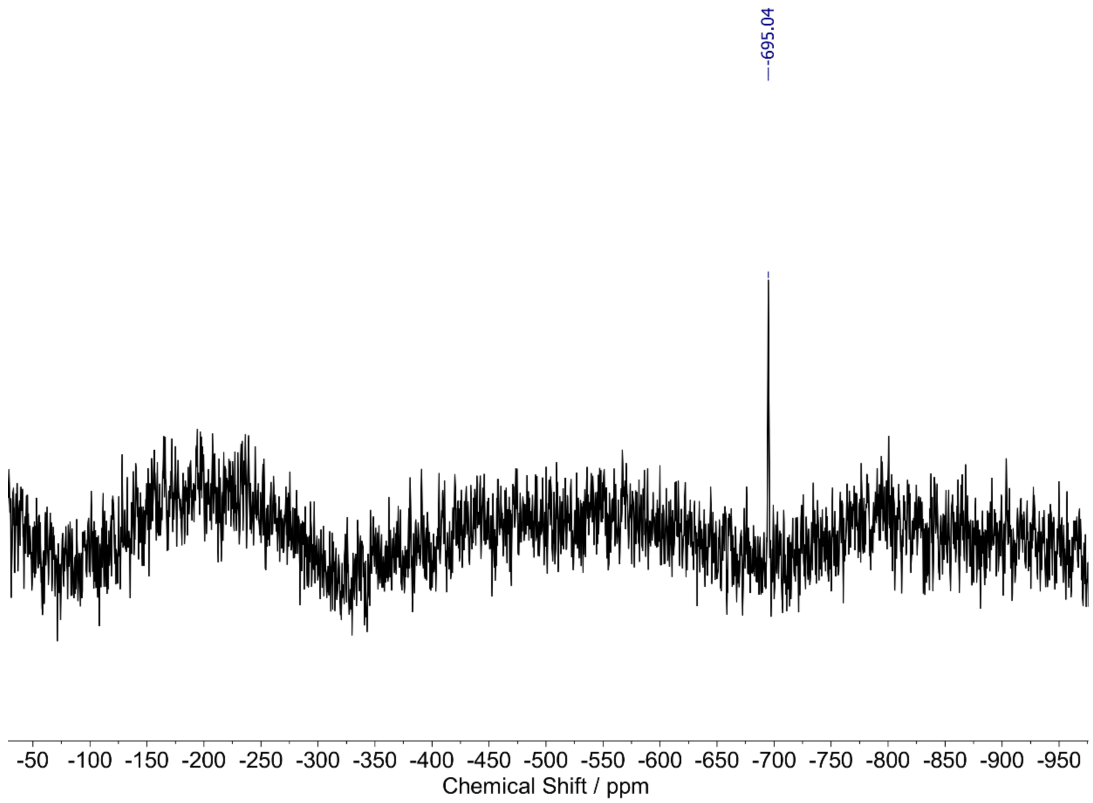

Figure S28: ^{199}Hg NMR (72 MHz, 25 °C) spectrum for **9** in C_6D_6.

S2.10. (F_3C-Ar$^{\#}$)$_2$Zn (10)

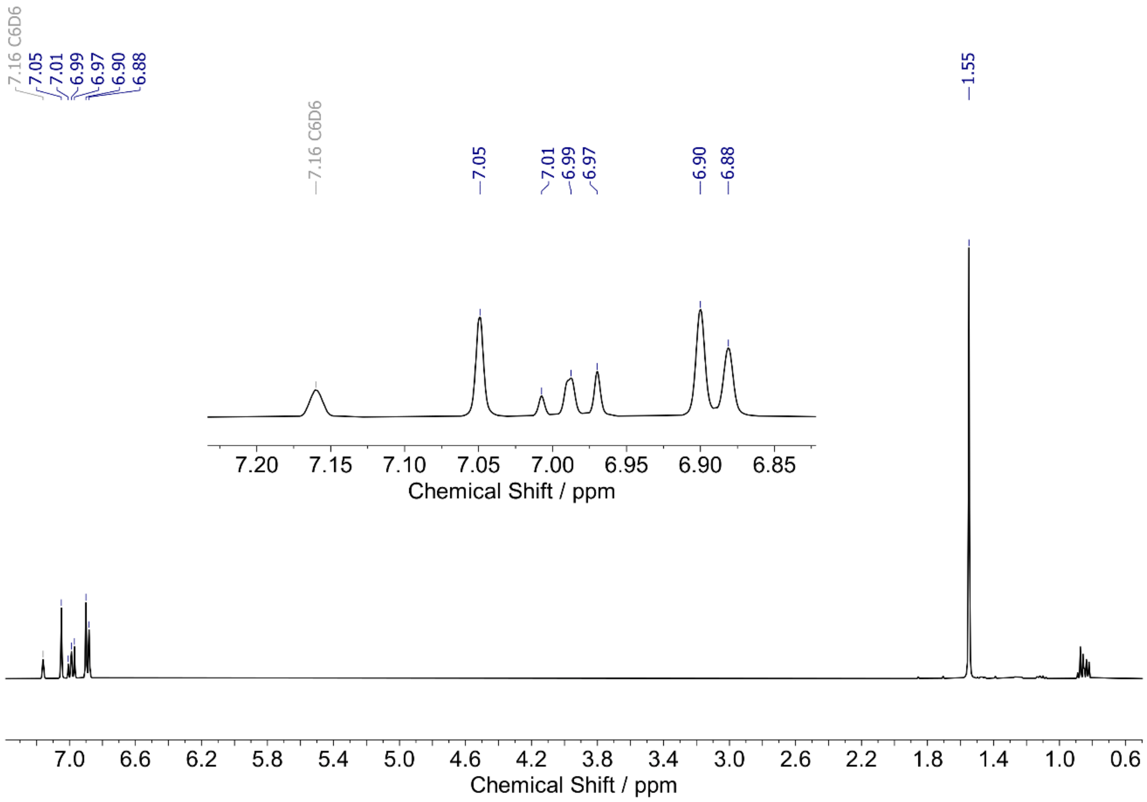

Figure S29: ^{1}H NMR (400 MHz, 25 °C) spectrum for **10** in C_6D_6.

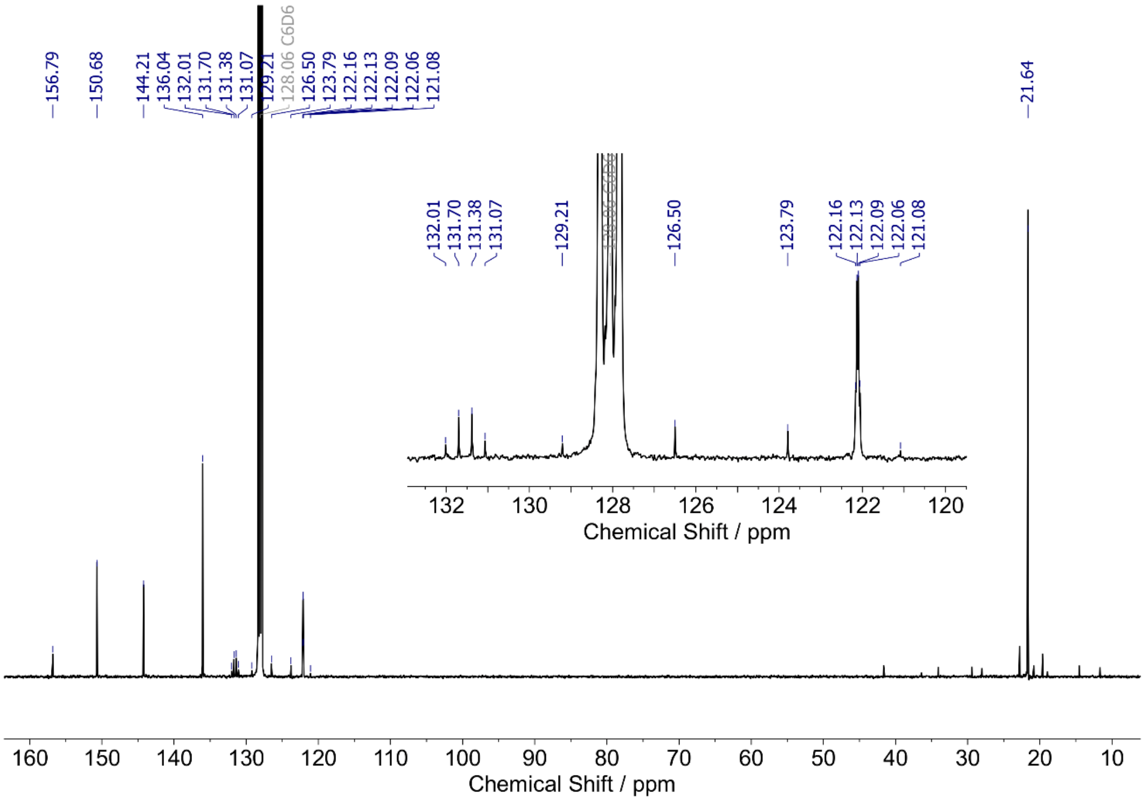

Figure S30: $^{13}C\{^{1}H\}$ NMR (101 MHz, 25 °C) spectrum for **10** in C_6D_6.

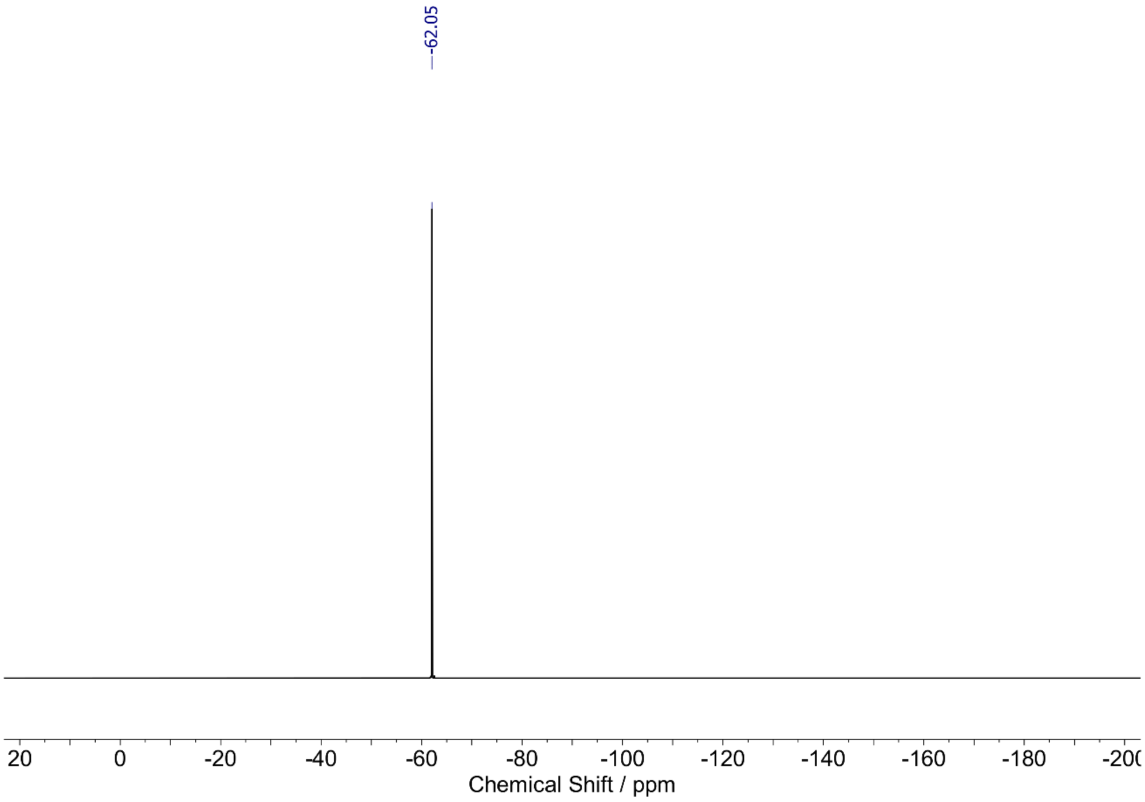

Figure S31: $^{19}F\{^{1}H\}$ NMR (376 MHz, 25 °C) spectrum for **10** in C_6D_6.

S2.11. (F_3C-Ar$^{\#}$)$_2$Cd (11)

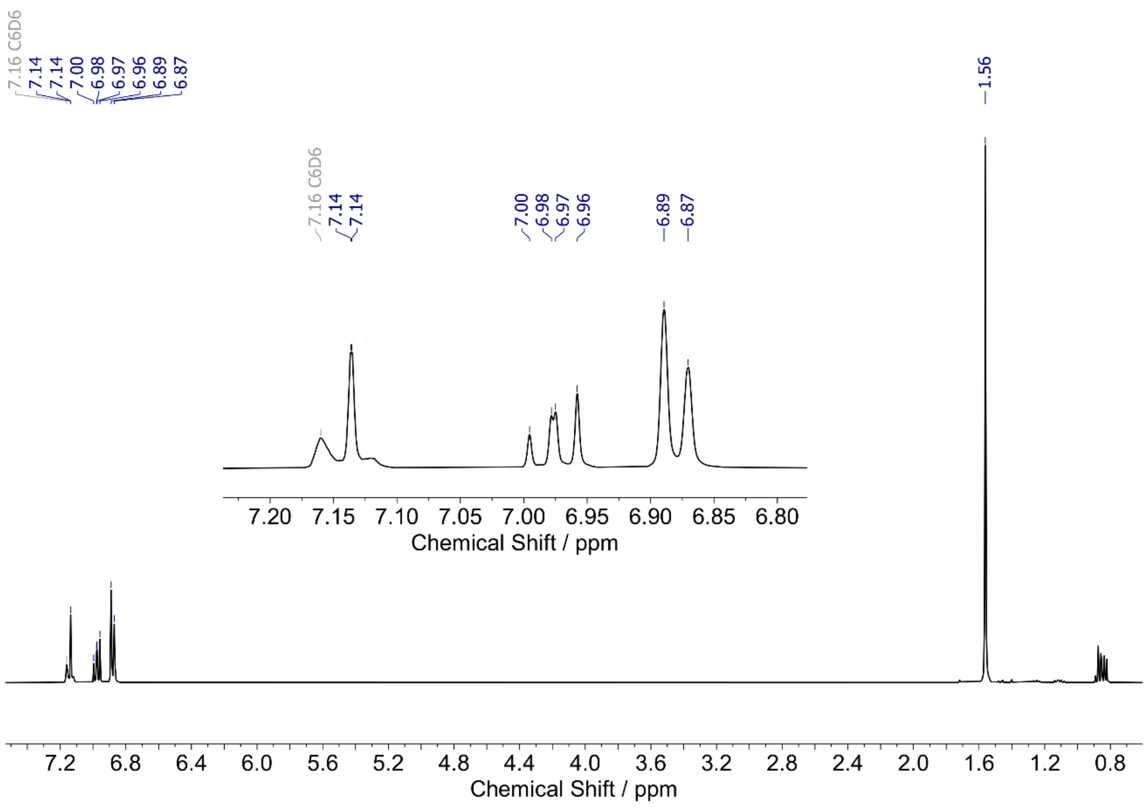

Figure S32: ^{1}H NMR (400 MHz, 25 °C) spectrum for **11** in C_6D_6.

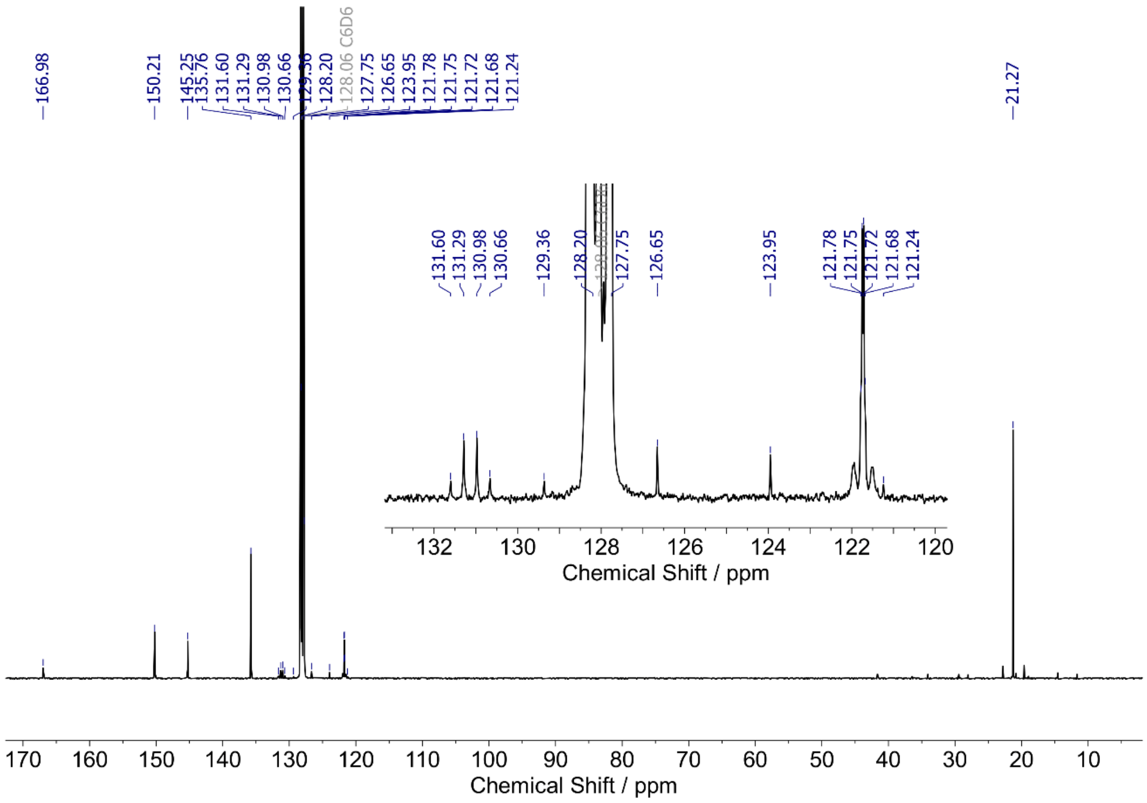

Figure S33: $^{13}C\{^{1}H\}$ NMR (101 MHz, 25 °C) spectrum for **11** in C_6D_6.

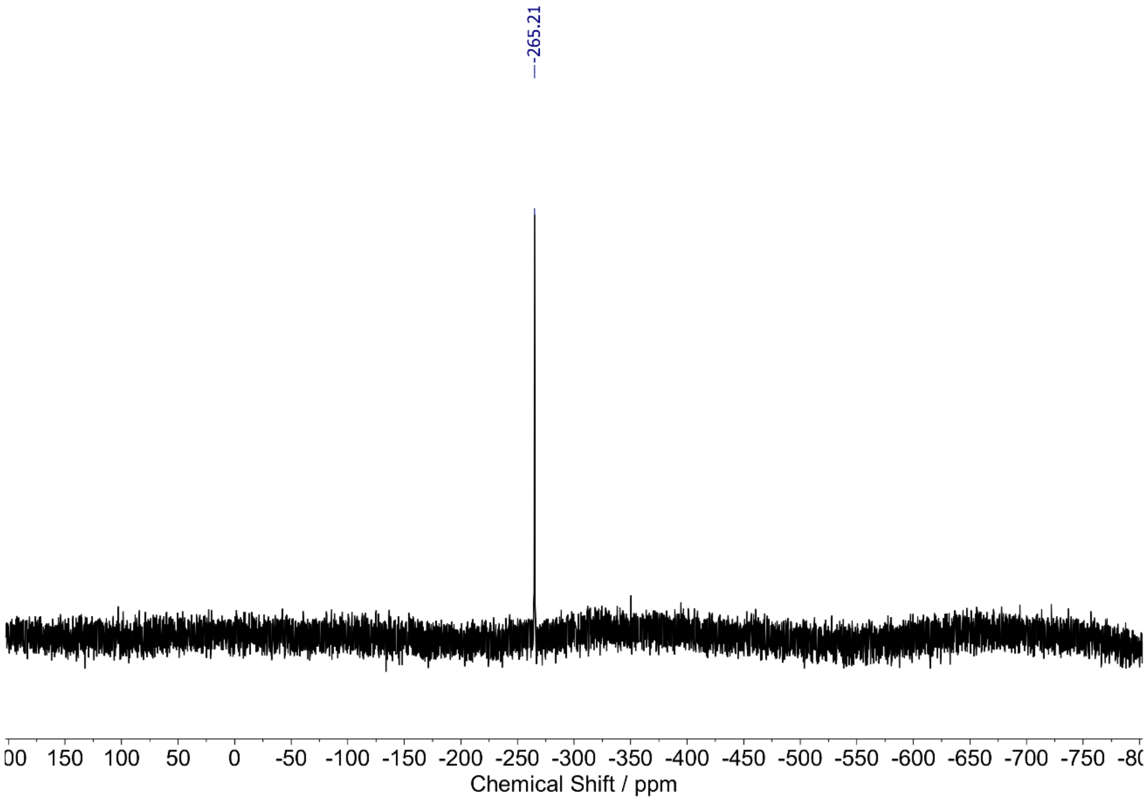

Figure S34: ^{113}Cd NMR (89 MHz, 25 °C) spectrum for **11** in C_6D_6.

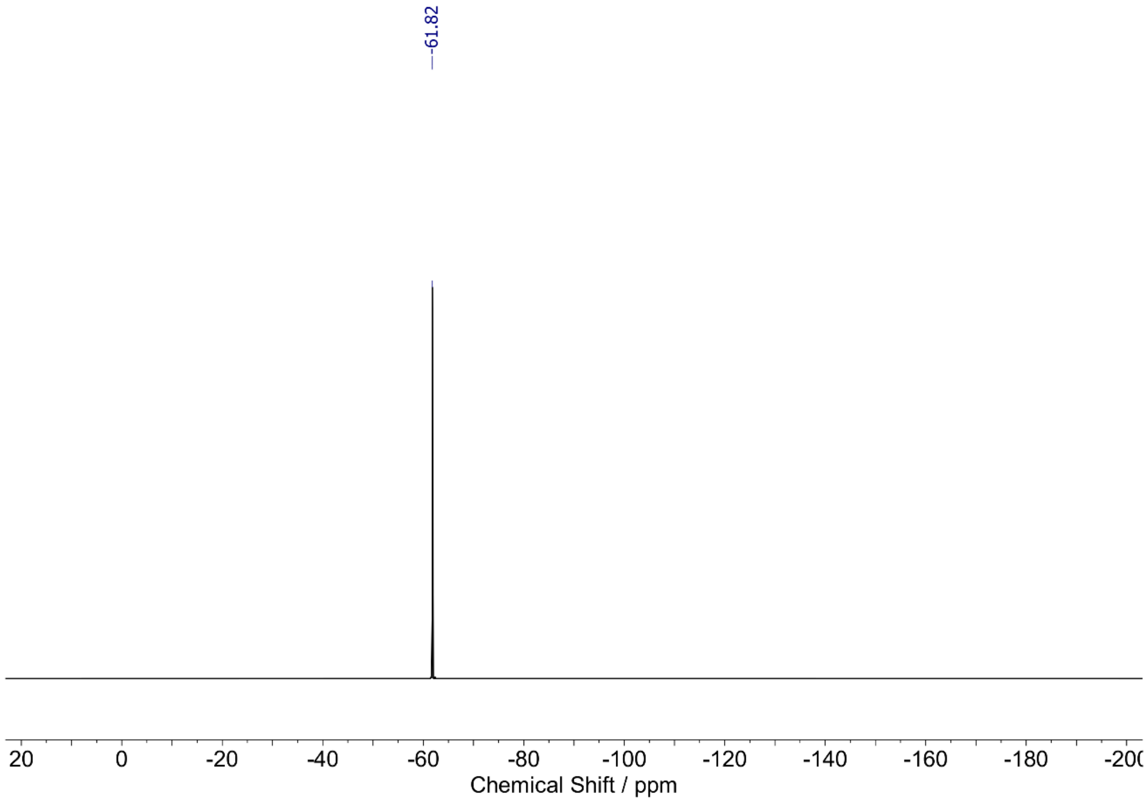

Figure S35: $^{19}F\{^{1}H\}$ NMR (376 MHz, 25 °C) spectrum for **11** in C_6D_6.

S2.12. $(F_3C\text{-}Ar^{\#})_2Hg$ (12)

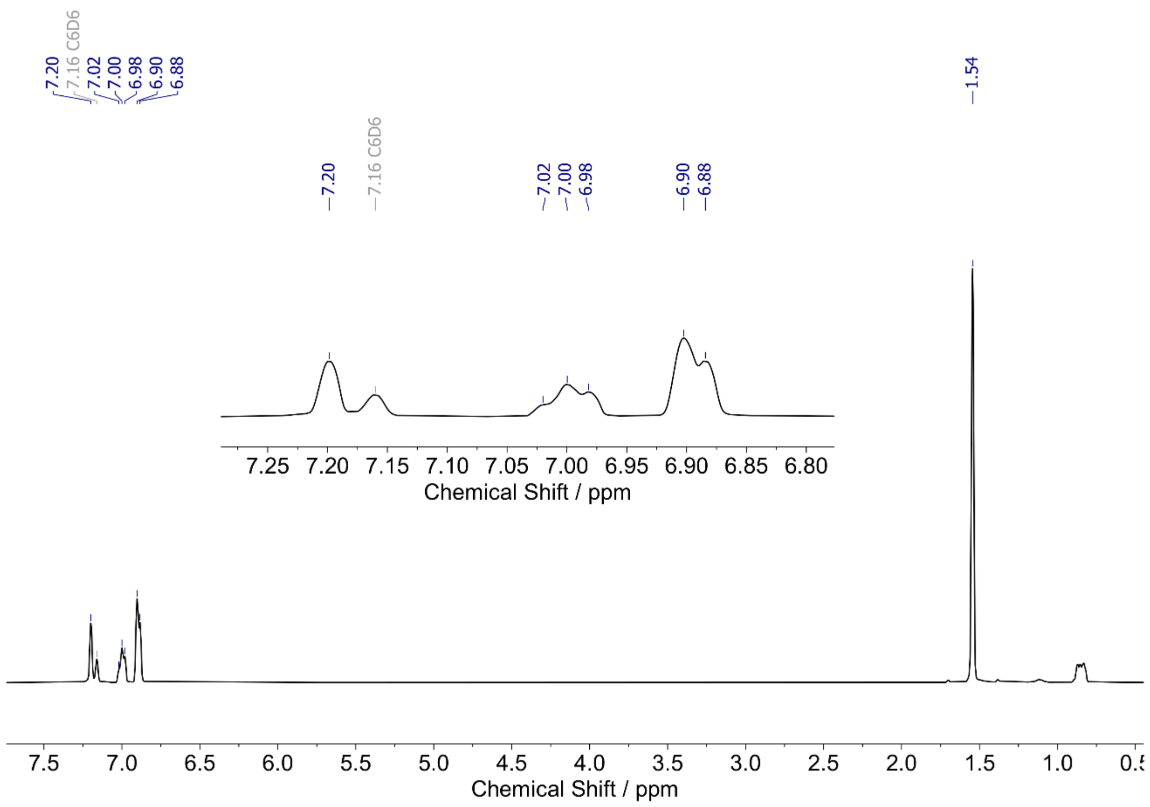

Figure S36: ^{1}H NMR (400 MHz, 25 °C) spectrum for **12** in C_6D_6.

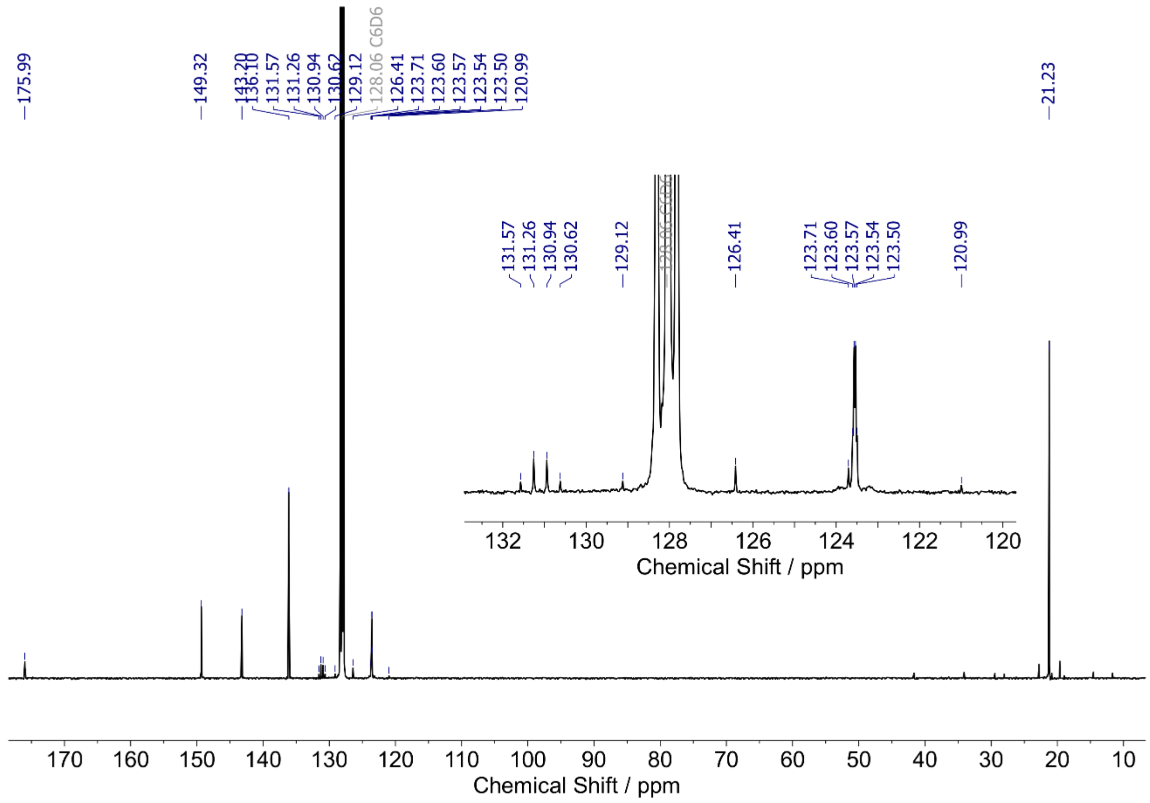

Figure S37: $^{13}C\{^1H\}$ NMR (101 MHz, 25 °C) spectrum for **12** in C_6D_6.

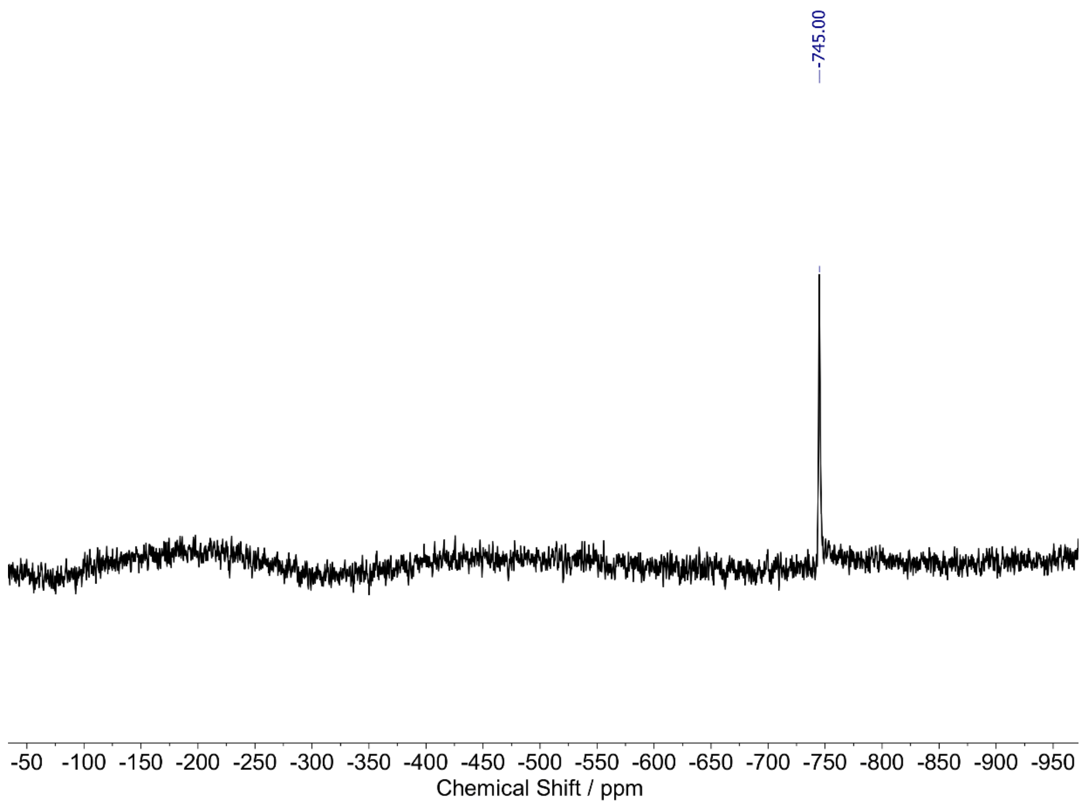

Figure S38: ^{199}Hg NMR (72 MHz, 25 °C) spectrum for **12** in C_6D_6.

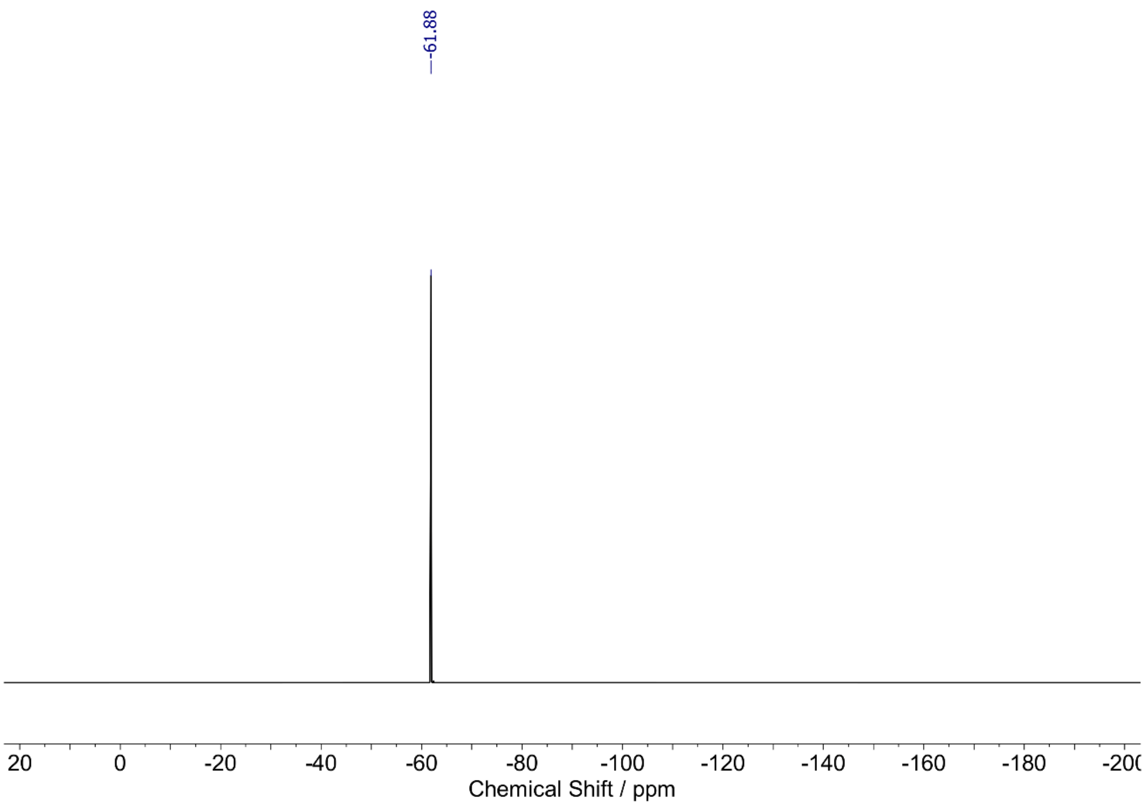

Figure S39: $^{19}F\{^{1}H\}$ NMR (376 MHz, 25 °C) spectrum for **12** in C_6D_6.

S3. Crystallography

S3.1. Crystallographic Methodology

Crystals of **1–12** were transferred into YR-1800 perfluoropolyether oil then mounted onto a MiTeGen MicroMount™ and cooled rapidly in a cold nitrogen stream using an Oxford Cryosystems open flow cryostat.[3] Diffraction data was collected using an Agilent SuperNova diffractometer (either using graphite-monochromated Mo-Kα radiation, λ = 0.71073 Å, ω scans; or using mirror-monochromated Cu-Kα radiation, λ = 1.54184 Å, ω scans) operating with either an Atlas, AtlasS2 or TitanS2 CCD area detector. Cell parameters were refined in each data set from the observed positions of all strong reflections, and Gaussian based absorption corrections with a beam profile correction (CrysAlisPro) were applied.[4] All non-H atoms were solved using direct or iterative methods, and refined using anisotropic displacement parameters, with the exception of some disordered atoms (see enclosed .cif files) which were instead refined isotropically. Hydrogen atoms were constrained in calculated positions and refined using a riding model. The crystal structures for complexes **1**, **2**, **10**, **11**, and **12** contained disordered hexane which could not be modelled satisfactorily and was masked using PLATON SQUEEZE[5] (structure **11**) or the SolventMask function implemented in OLEX2 (structures **1**, **2**, **10**, and **12**).[6] Programs used include CrysAlisPro[4] (control and integration), OLEX2[6] (molecular graphics), SHELXT[7] (structure solution) and SHELXL[8] (structure refinement). CIF files were checked by Dr William Lewis and Dr Stephen Argent at the University of Nottingham Crystal Structure Service. CCDC-2163371-2163382 contains the supplementary data for the complexes. These data can be obtained free of charge from The Cambridge Crystallographic Data Centre via www.ccdc.cam.ac.uk/data_request/cif.

S3.2. Crystallographic Data

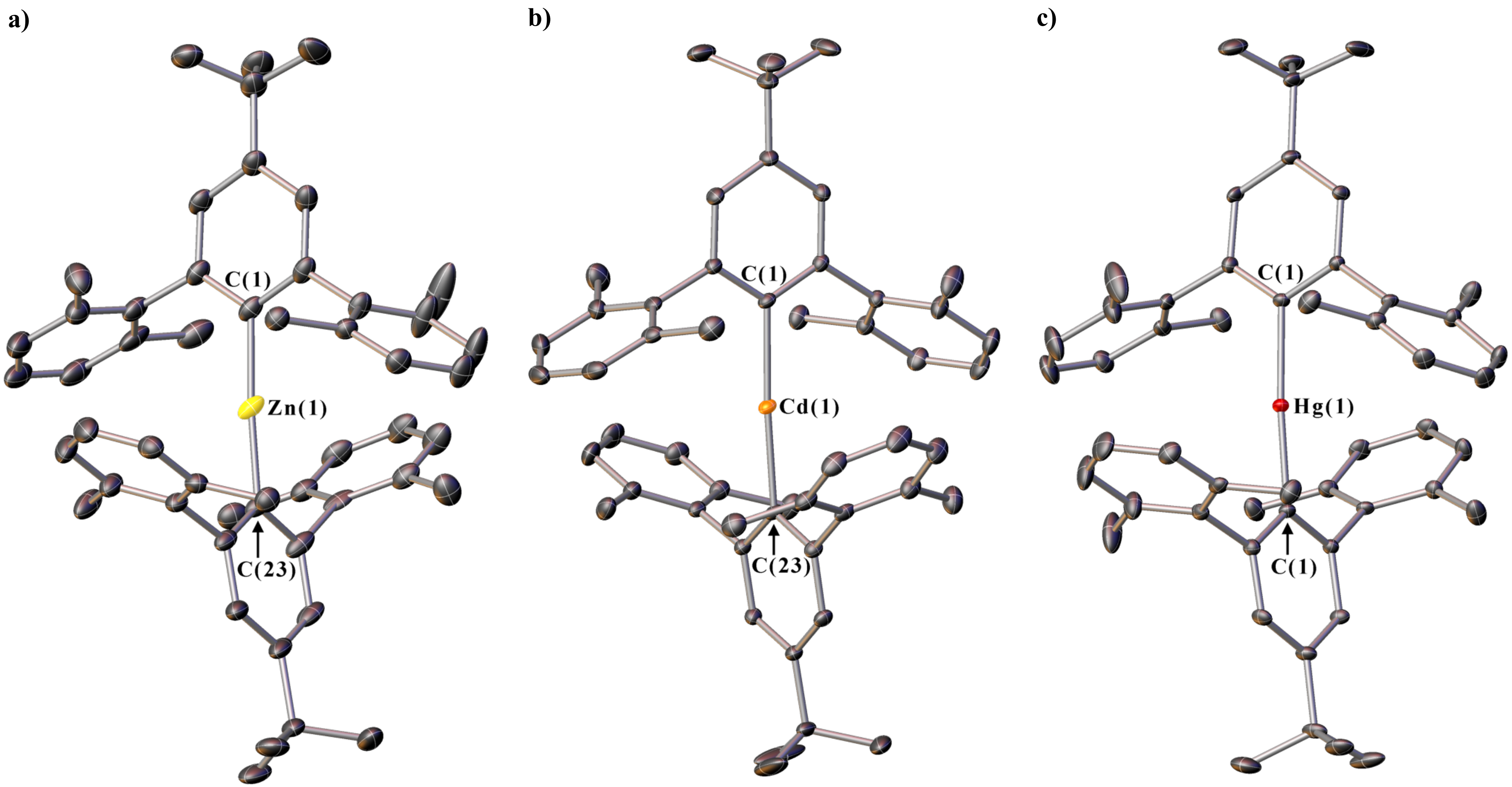

Figure S40: Crystal structures of the *m*-terphenyl Group 12 complexes, (*t*-Bu-Ar[#])$_2$M, for a) M = Zn (**1**), b) M = Cd (**2**) and c) M = Hg (**3**). Ellipsoids set at 30% probability. Hydrogen atoms and residual solvent molecules are omitted for clarity. When necessary, only one molecule from the asymmetric unit is shown. For **3**, only half the molecule is in the asymmetric unit, where C(23) = C(1).

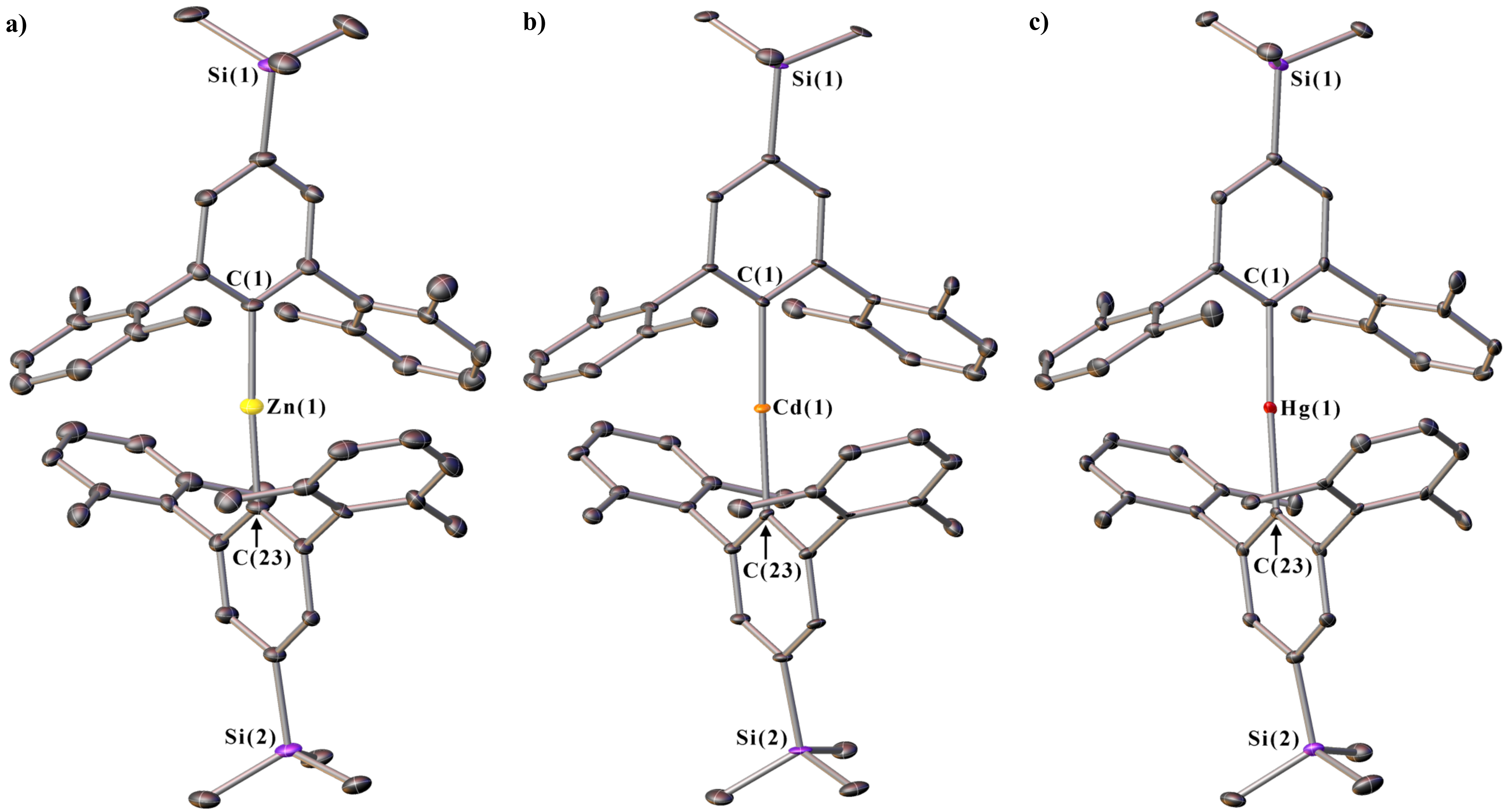

Figure S41: Crystal structures of the *m*-terphenyl Group 12 complexes, $(Me_3Si\text{-}Ar^{\#})_2M$, for a) M = Zn (**4**), b) M = Cd (**5**) and c) M = Hg (**6**). Ellipsoids set at 30% probability. Hydrogen atoms and residual solvent molecules are omitted for clarity. When necessary, only one molecule from the asymmetric unit is shown.

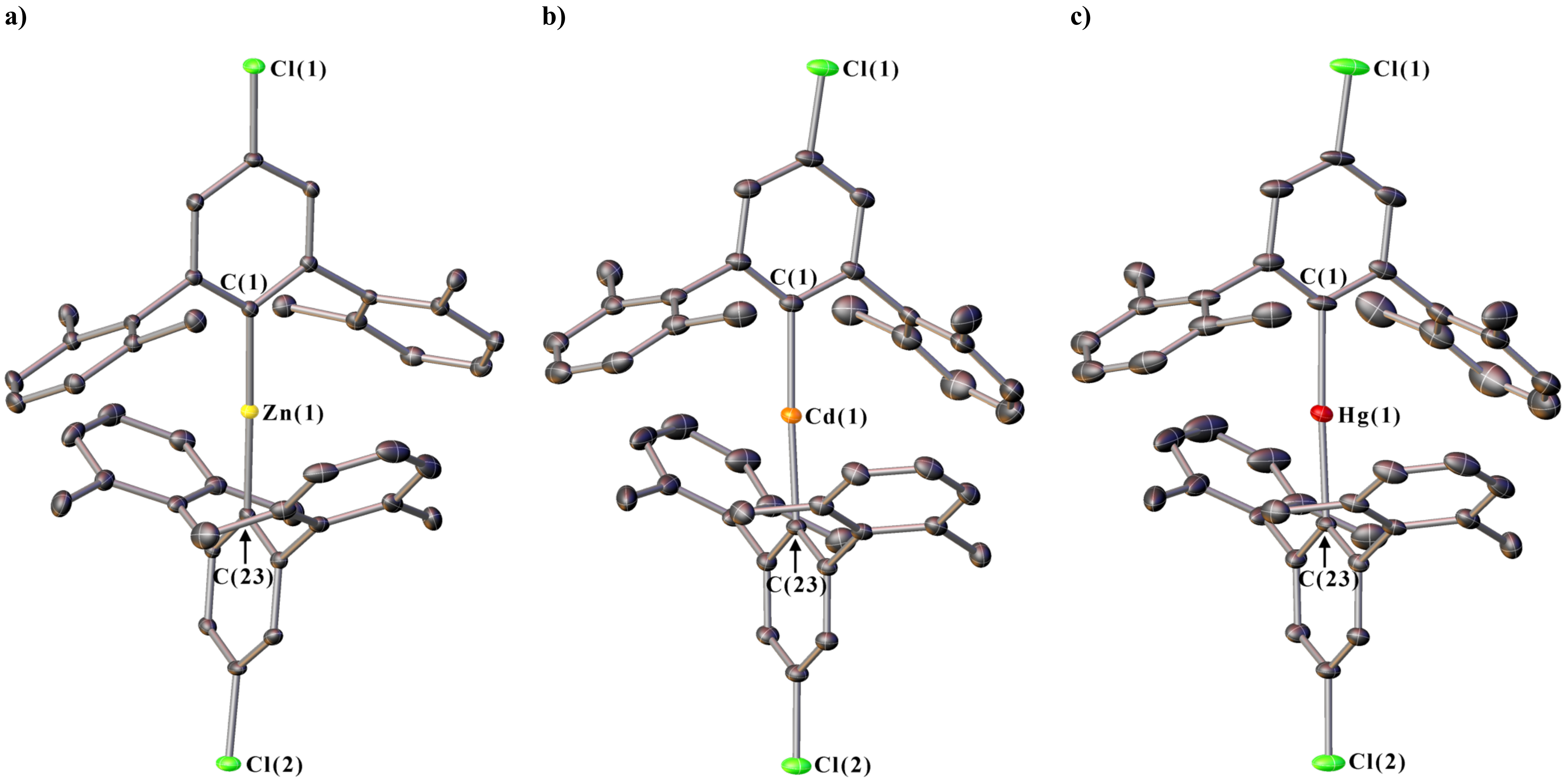

Figure S42: Crystal structures of the *m*-terphenyl Group 12 complexes, $(Cl\text{-}Ar^{\#})_2M$, for a) M = Zn (**7**), b) M = Cd (**8**) and c) M = Hg (**9**). Ellipsoids set at 30% probability. Hydrogen atoms and residual solvent molecules are omitted for clarity. When necessary, only one molecule from the asymmetric unit is shown.

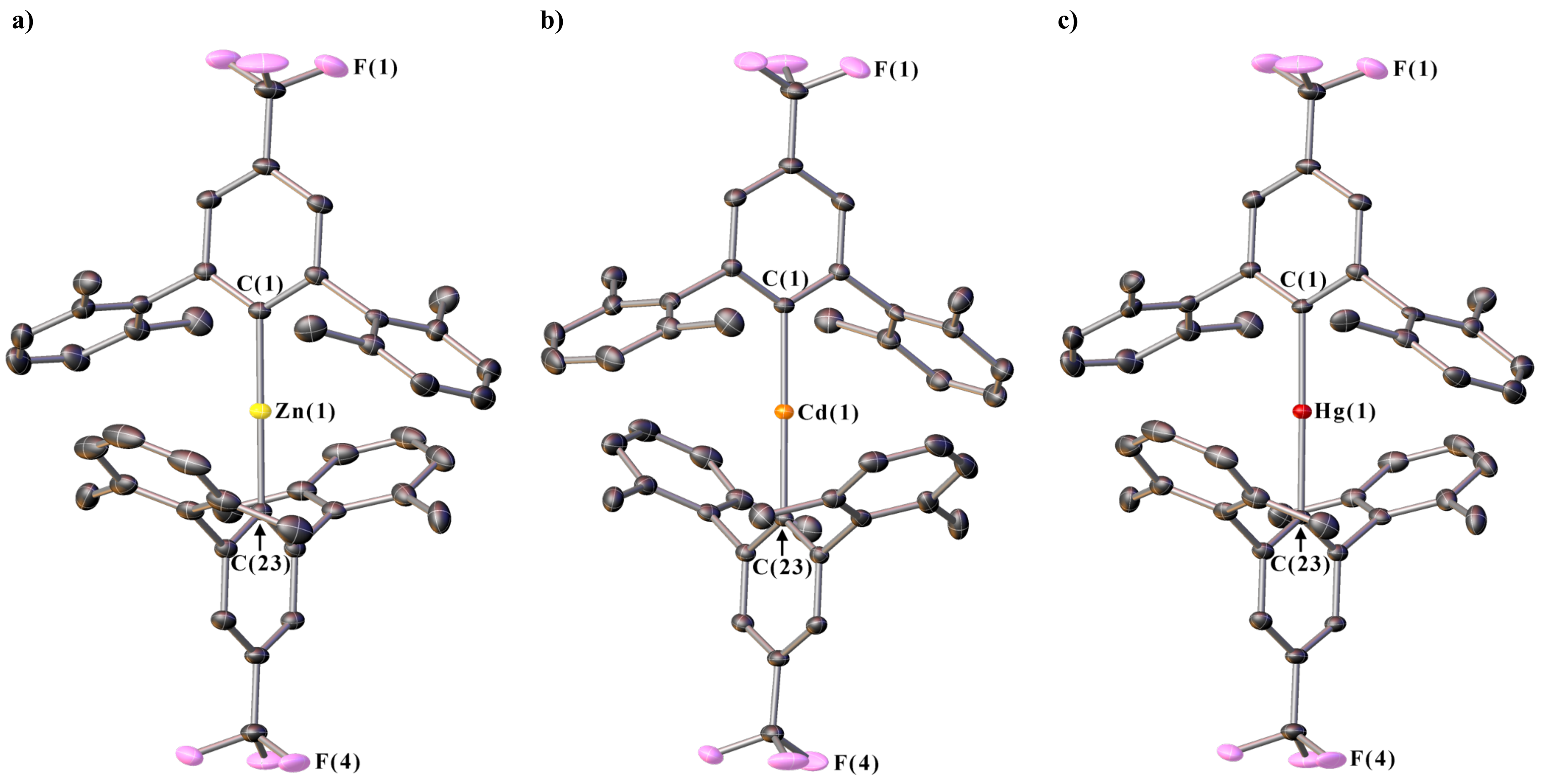

Figure S43: Crystal structures of the *m*-terphenyl Group 12 complexes, $(F_3C\text{-}Ar^{\#})_2M$, for a) M = Zn (**10**), b) M = Cd (**11**) and c) M = Hg (**12**). Ellipsoids set at 30% probability. Hydrogen atoms and residual solvent molecules are omitted for clarity. When necessary, only one molecule from the asymmetric unit is shown.

Table S1: Selected bond lengths (Å) and angles (°) for the zinc aryls (R-Ar[#])$_2$Zn (**1**, **4**, **7**, **10**).

M = Zn	Bond Lengths (Å) and Angles (°)			
	**1** (R = *t*-Bu)	**4**[b] (R = SiMe$_3$)	**7**[c] (R= Cl)	**10** (R = CF$_3$)
Zn(1)–C(1)	1.937(2)	1.953(12)	1.9418(17)<br>[1.9429(17)]	1.9449(13)
Zn(1)–C(23)	1.934(2)	1.951(13)	1.9465(17)<br>[1.9367(17)]	1.9483(13)
C(1)–Zn(1)–C(23)	175.87(10)	176.4(6)	176.10(8)<br>[176.84(9)]	178.87(6)
C(1)-aryl plane ⋯C(23)-aryl plane	83.06(8)	78.0(4)	89.26(7)<br>[83.13(7)]	83.10(5)
C(1)-aryl plane ⋯flanking aryl plane	87.4(5)[a]<br>84.72(17)[a]<br>76.66(10)	82.4(5)<br>73.7(4)	78.66(7)<br>69.52(7)<br>[76.09(7)]<br>[86.30(7)]	83.21(5)<br>80.3(2)[a]<br>84.4(3)[a]
C(23)-aryl plane ⋯flanking aryl plane	75.04(8)<br>76.7(3)<br>84.1(2)	83.5(4)<br>70.5(5)	87.35(7)<br>83.33(7)<br>[78.18(7)]<br>[82.86(7)]	83.97(6)<br>88.67(5)

[a] Complexes **1** and **10** have additional 'C(1)-aryl plane⋯flanking aryl plane' angles due to their disordered flanking rings.
[b] Crystal data for **4** is of low quality due to weak diffraction from a very small crystal. Data is included here for completeness.
[c] For **7**, measurements for the second molecule of the asymmetric unit is in square brackets.

Table S2: Selected bond lengths (Å) and angles (°) for the cadmium aryls (R-Ar[#])$_2$Cd (**2**, **5**, **8**, **11**).

M = Cd	Bond Lengths (Å) and Angles (°)			
	**2** (R = *t*-Bu)	**5** (R = SiMe$_3$)	**8** (R= Cl)	**11** (R = CF$_3$)
Cd(1)–C(1)	2.110(2)	2.111(14)	2.120(2)	2.1159(16)
Cd(1)–C(23)	2.110(2)	2.098(14)	2.116(2)	2.1215(16)
C(1)–Cd(1)–C(23)	176.57(7)	177.5(6)	177.42(10)	179.16(6)
C(1)-aryl plane ⋯C(23)-aryl plane	81.91(7)	79.5(5)	87.23(8)	80.22(6)
C(1)-aryl plane ⋯flanking aryl plane	84.31(8)<br>73.69(8)	83.5(5)<br>72.3(5)	86.62(9)<br>85.90(10)	81.98(7)<br>81.64(12)[a]<br>84.6(3)[a]
C(23)-aryl plane ⋯flanking aryl plane	76.42(7)<br>83.89(8)	83.1(5)<br>75.9(5)	75.64(9)<br>79.38(8)	84.81(7)<br>88.69(6)

[a] Complex **11** has additional 'C(1)-aryl plane⋯flanking aryl plane' angles due to its disordered flanking rings.

Table S3: Selected bond lengths (Å) and angles (°) for the mercury aryls (R-Ar$^{\#}$)$_2$Hg (**3**, **6**, **9**, **12**).

M = Hg	Bond Lengths (Å) and Angles (°)			
	**3**[a] (R = *t*-Bu)	**6** (R = $SiMe_3$)	**9** (R= Cl)	**12** (R = CF_3)
Hg(1)–C(1)	2.070(3)	2.056(10)	2.086(3)	2.089(3)
Hg(1)–C(23)	-	2.063(10)	2.085(3)	2.098(3)
C(1)–Hg(1)–C(23)	175.91(13)	177.1(4)	177.51(14)	179.28(12)
C(1)-aryl plane ···C(23)-aryl plane	78.54(12)	79.8(3)	87.53(13)	81.06(12)
C(1)-aryl plane ···flanking aryl plane	87.32(11)<br>77.03(9)	84.1(4)<br>73.3(3)	86.48(16)<br>85.45(16)	83.17(13)<br>81.3(3)[b]<br>84.3(6)[b]
C(23)-aryl plane ···flanking aryl plane	-<br>-	84.1(3)<br>77.1(4)	76.80(14)<br>81.74(12)	85.62(15)<br>88.17(12)

[a] For **3**, C(23) = C(1) due to only half the molecule in the asymmetric unit.

[b] Complex **12** has additional 'C(1)-aryl plane···flanking aryl plane' angles due to its disordered flanking rings.

Table S4: Crystallographic data for compounds **1–12.**

	**1**	**2**	**3**	**4**	**5**	**6**
Internal code	ZNAJVA	CDAJVA	HGLJTB	ZNAJVB	CDAJVB	HGLJTA
Formula	$C_{58}H_{72}Zn$	$C_{61}H_{79}Cd$	$C_{52}H_{58}Hg$	$C_{50}H_{58}Si_2Zn$	$C_{50}H_{58}Si_2Cd$	$C_{50}H_{58}Si_2Hg$
M_w	834.52	924.64	883.57	780.51	827.54	915.73
T (K)	120(2)	120.00(12)	120(2)	120(2)	120(2)	120(2)
Crystal system	triclinic	triclinic	monoclinic	triclinic	triclinic	triclinic
Space group	P-1	P-1	C2/c	P-1	P-1	P-1
a (Å)	12.1182(4)	12.1241(4)	17.1838(12)	11.658(3)	11.5086(5)	11.5367(6)
b (Å)	15.1384(5)	15.2935(5)	17.9336(10)	13.804(4)	13.9916(7)	13.8974(13)
c (Å)	15.2972(5)	15.4933(5)	14.9725(9)	16.423(4)	16.5734(13)	16.6240(10)
α (°)	113.284(3)	112.638(3)	90	70.32(2)	107.205(6)	107.185(7)
β (°)	101.454(3)	102.448(3)	111.102(7)	85.79(2)	94.247(5)	94.297(5)
γ (°)	103.427(3)	104.576(3)	90	65.06(3)	114.241(5)	114.482(7)
V (Å^3)	2371.15(15)	2404.03(15)	4304.6(5)	2249.2(11)	2265.2(3)	2256.9(3)
Z	2	2	4	2	2	2
D_{calc} (g cm^{-3})	1.169	1.099	1.363	1.152	1.213	1.348
μ (mm^{-1})	0.974	3.913	3.609	1.491	4.589	3.494
F_{000}	900.0	942.0	1800	832.0	868.0	932.0
Crystal size (mm^3)	0.44 × 0.16 × 0.15	0.16 × 0.08 × 0.07	0.23 × 0.10 × 0.06	0.10 x 0.07 x 0.02	0.16 × 0.10 × 0.02	0.19 × 0.14 × 0.03
λ (Å)	1.54184	1.54184	0.71073	1.54184	1.54184	0.71073
2θ range for data collection (°)	6.958 to 147.238	6.744 to 147.308	7.276 to 56.898	7.504 to 149.406	7.424 to 133.154	5.84 to 55.286
Reflections collected	18037	22993	10885	8994	8009	14444
Independent reflections	9293	9432	4561	8994	8009	14444
R_{int}	0.0351	0.0281	0.0305	-	-	-
$GooF$ on F^2	1.035	1.025	1.043	1.019	1.192	1.021
R_1, wR_2 [$I > 2\sigma(I)$]	0.0456, 0.1186	0.0273, 0.0693	0.0256, 0.0528	0.1369, 0.3163	0.1138, 0.2924	0.0608, 0.1436
R_1, wR_2 (all data)	0.0526, 0.1250	0.0293, 0.0708	0.0290, 0.0542	0.2531, 0.3984	0.1280, 0.2991	0.0768, 0.1507
Largest diff. peak/hole (e Å^{-3})	0.38/–0.74	0.44/–0.71	0.92/–1.43	1.56/–1.42	4.18/–2.48	2.90/-2.07

	**7**	**8**	**9**	**10**	**11**	**12**
Internal Code	KEWJME	KEWJMF	KEWJMD	ZNAJVC	CDAJVC	HGAJVC
Formula	$C_{44}H_{40}Cl_2Zn$	$C_{44}H_{40}Cl_2Cd$	$C_{44}H_{40}Cl_2Hg$	$C_{49}F_6H_{47}Zn$	$C_{49}F_6H_{47}Cd$	$C_{46}H_{40}F_6Hg$
M_w	705.03	752.06	840.25	772.15	819.18	907.37
T (K)	120(2)	120(2)	120(2)	120(2)	120(2)	120(2)
Crystal system	monoclinic	monoclinic	monoclinic	monoclinic	monoclinic	monoclinic
Space Group	$P2_1/n$	$P2_1/c$	$P2_1/c$	$P2_1/c$	$P2_1/c$	$P2_1/c$
a (Å)	17.8312(2)	10.4856(3)	10.4595(2)	11.59590(10)	11.5314(2)	11.6032(2)
b (Å)	19.6421(3)	19.5184(5)	19.5671(6)	17.77240(10)	17.9866(2)	17.9142(2)
c (Å)	20.8076(3)	17.9884(5)	18.0162(4)	20.6031(2)	20.5675(3)	20.5586(2)
α (°)	90	90	90	90	90	90
β (°)	100.9640(10)	98.895(2)	99.208(2)	100.6780(10)	100.422(2)	100.9020(10)
γ (°)	90	90	90	90	90	90
V (Å^3)	7154.68(17)	3637.27(17)	3639.72(16)	4172.51(6)	4195.54(11)	4196.23(10)
Z	8	4	4	4	4	4
D_{calc} (g cm^{-3})	1.309	1.373	1.533	1.229	1.297	1.436
μ (mm^{-1})	2.544	6.374	9.150	1.288	4.638	7.049
F_{000}	2944	1544.0	1672.0	1600.0	1884.0	1800
Crystal size (mm^3)	0.09 × 0.05 × 0.04	0.33 × 0.18 × 0.14	0.24 × 0.09 × 0.07	0.59 × 0.43 × 0.37	0.30 × 0.14 × 0.07	0.10 × 0.08 × 0.03
λ (Å)	1.54184	1.54184	1.54184	1.54184	1.54184	1.54184
2θ range for data collection (°)	6.764 to 147.226	6.726 to 149.632	6.716 to 148.878	6.618 to 149.172	8.742 to 149.012	6.596 to 147.4
Reflections collected	47824	15158	26249	135378	62977	22229
Independent reflections	14178	7217	7305	8490	8533	8283
R_{int}	0.0422	0.0282	0.0357	0.0524	0.0312	0.0331
$GooF$ on F^2	1.016	1.035	1.017	1.045	1.019	1.013
R_1, wR_2 [$I > 2\sigma(I)$]	0.0352, 0.0789	0.0326, 0.0844	0.0273, 0.0672	0.0342, 0.0954	0.0238, 0.0596	0.0277, 0.0572
R_1, wR_2 (all data)	0.0517, 0.0859	0.0363, 0.0876	0.0343, 0.0709	0.0352, 0.0966	0.0295, 0.0636	0.0451, 0.0627
Largest diff. peak/hole (e Å^{-3})	0.29/–0.38	1.03/–0.88	0.77/–0.62	0.36/–0.43	0.36/–0.48	0.79/–0.78

S4. Cyclic Voltammetry

CV samples of **3** and **12** were prepared within the glove box under dinitrogen as 1.0 mM solutions in THF containing 0.5 M [*n*-Bu_4N][BF_4] electrolyte. The solutions were sealed inside a single-compartment electrochemical cell that functioned as a three-electrode system comprising of glassy carbon working and counter electrodes, alongside a Hg/Hg_2Cl_2 reference electrode (SCE) that was chemically isolated from the sample solution via a bridge tube containing electrolyte solution and fitted with a porous Vycor frit. CV experiments were carried out in conjunction with Dr E. Stephen Davies at the University of Nottingham using an Autolab PGSTAT320N potentiostat. Redox potentials are referenced to the ferrocenium-ferrocene (Fc^+/Fc) couple by an internal calibration.

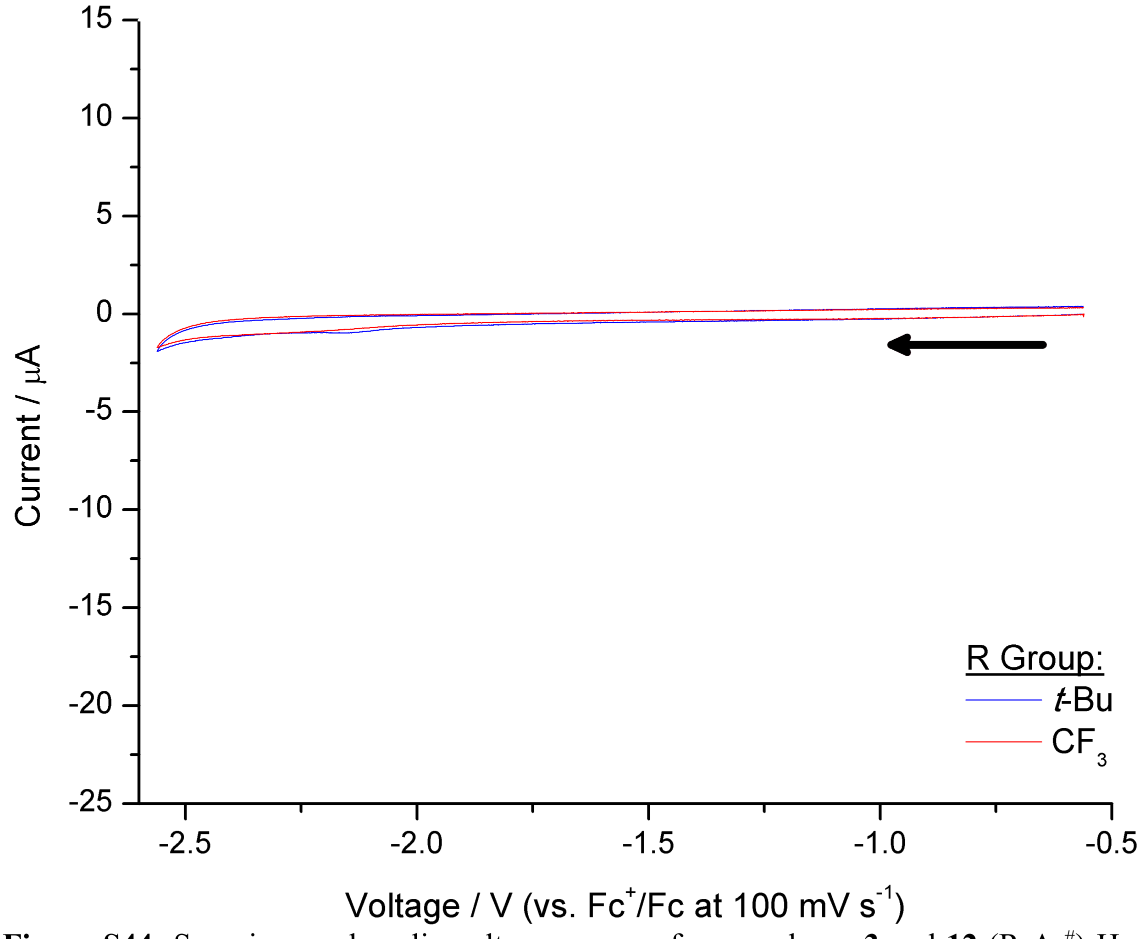

Figure S44: Superimposed cyclic voltammagrams for complexes **3** and **12** (R-$Ar^{\#}$)$_2$Hg (R = *t*-Bu, CF_3). Samples measured in THF (1.0 M) containing [*n*-Bu_4N][BF_4] (0.5 M) at 100 mV/s scan rate.

S5. Computational Work

S5.1. Geometry Optimization Methodology

Geometry optimizations were performed for models of **1–12** and $(H\text{-}Ar^{\#})_2M$ (M = Zn, Cd, Hg) using coordinates derived from their corresponding X-ray crystal structures (reported here or taken from the literature[9]). We computed structures that were both fully optimized, where the positions of all atoms were relaxed, and partially optimized, where only the H atom positions were relaxed (H-atom optimized).

For the fully optimized structures, the models were geometry optimized without restraints using the ORCA program.[10,11] The DFT geometry optimization was performed using the BP86 density functional[12,13] with RI-J acceleration[14,15] and employed all-electron ZORA corrected[16] TZVP basis sets[17–19] for C, H, Zn, and Cd; and all-electron ZORA[16] corrected SARC-TZVP[20] basis sets for Hg. Auxiliary SARC/J basis sets decontracted def2/J up to Kr[21] and SARC auxiliary basis sets beyond Kr were also employed.[20,22–24] Gradient corrections were performed with Grimme's 3rd generation dispersion correction.[25,26] The optimizations were performed with TightOpt, TightSCF, and DFT integration settings of Grid4, FinalGrid5. Frequency analysis revealed no imaginary vibrations for any structures. Geometry optimized coordinates can be found in section S5.4. A summary of key bond lengths and angles for these fully optimized structures is provided in Table S5.

For the H-atom optimized structures, optimizations were carried out using the ORCA program.[10,11] These optimizations were performed at the (non-relativistic) density-functional level using the PBE0[27,28] functional with RI-J acceleration of the Coulomb contribution[14,15] and COSX acceleration of the exchange contribution.[29] The auxiliary basis sets of Weigend[21] were used for all density-fitting (RI) contributions and Grimme's D3 dispersion correction

was included with the Becke-Johnson damping function (D3-BJ).[26,25] The def2-TZVP basis set[19] was used and the calculations were performed on 32 cores using MPI starting from the crystallographic structures. Coordinates for these H-atom optimized structures can be found in section S5.5.

Table S5: C–M–C bond angles (°) and C–M bond lengths (Å) for fully geometry optimized structures of Group 12 diaryl complexes

M	R	C–M–C (°)	C–M (Å)[a]
Zn	*t*-Bu	178.38	1.929
	$SiMe_3$	178.78	1.932
	H	178.49	1.934 [1.933]
	Cl	178.88	1.931
	CF_3	178.18	1.934
Cd	*t*-Bu	179.08	2.116
	$SiMe_3$	179.63	2.119 [2.118]
	H	179.46	2.120
	Cl	179.77	2.117
	CF_3	179.90	2.120
Hg	*t*-Bu	179.77	2.094
	$SiMe_3$	179.74	2.097 [2.096]
	H	179.88	2.098
	Cl	179.84	2.095 [2.094]
	CF_3	179.57	2.097

[a]In most cases, both C–M bond lengths were identical to 4 s.f. in the optimized structures. Where there was a difference, the smaller bond length is given in square brackets.

S5.2. Orbital Energy and QTAIM Calculations

S5.2.1. Methodology

Single point calculations were performed on the fully optimized model geometries of **1–12** and (H-Ar$^{\#}$)$_2$M (M = Zn, Cd, Hg) described above using the ORCA software package.[10,11] Calculations employed the PBE0 density functional[27,28] and all-electron ZORA corrected[16] def2-TZVP basis sets[17–19,30] for all atoms (except Hg), ZORA-SARC-TZVP basis set for Hg atoms,[20] along with SARC/J auxiliary basis sets decontracted def2/J up to Kr[21] and SARC auxiliary basis sets beyond Kr.[20,22–24] Gradient corrections were performed with Grimme's 3rd generation dispersion correction.[25] AIM analysis was performed on (H-Ar$^{\#}$)$_2$M (M = Zn, Cd, Hg) using the MultiWFN 3.6 software package.[31]

S5.2.2. Orbital Energy Plots

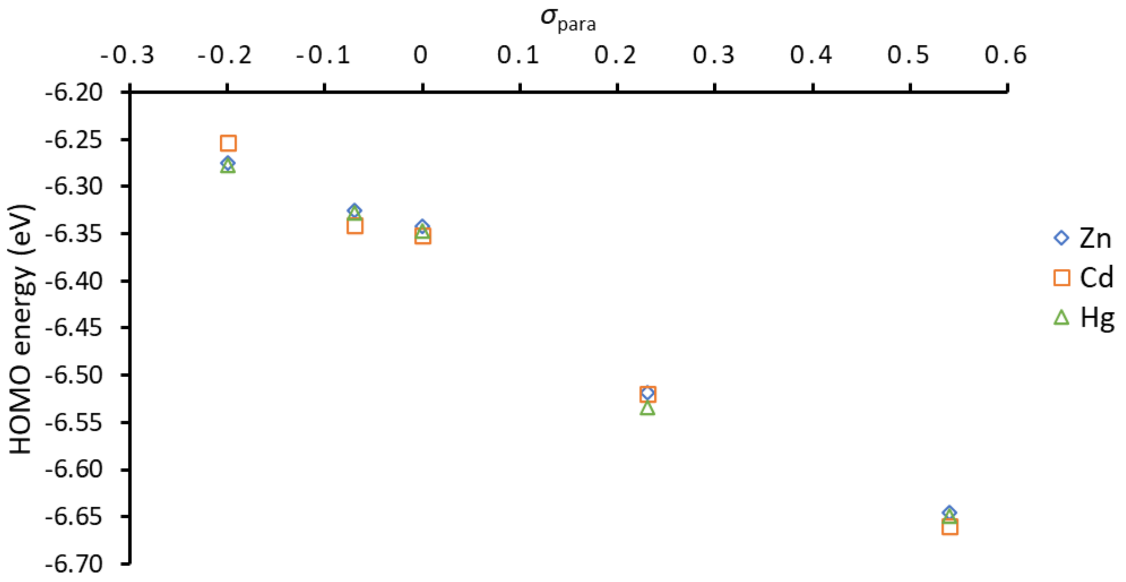

Figure S45: Graph of computed HOMO energies (eV) versus Hammet constant of *para*-substituent (σ_{para}) for Group 12 *m*-terphenyl complexes.

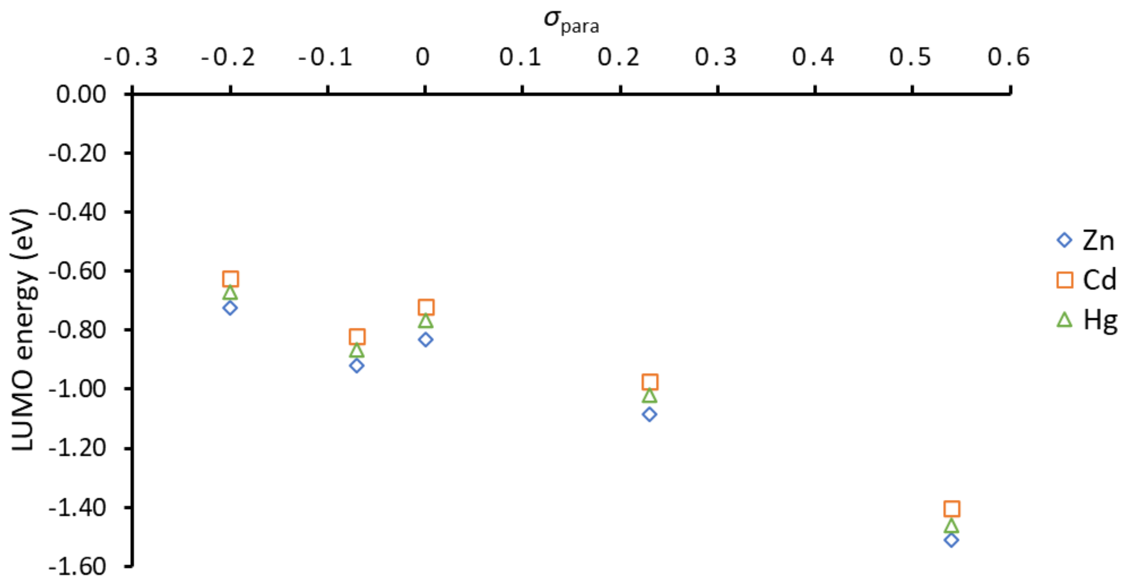

Figure S46: Graph of computed LUMO energies (eV) versus Hammet constant of *para*-substituent (σ_{para}) for Group 12 *m*-terphenyl complexes.

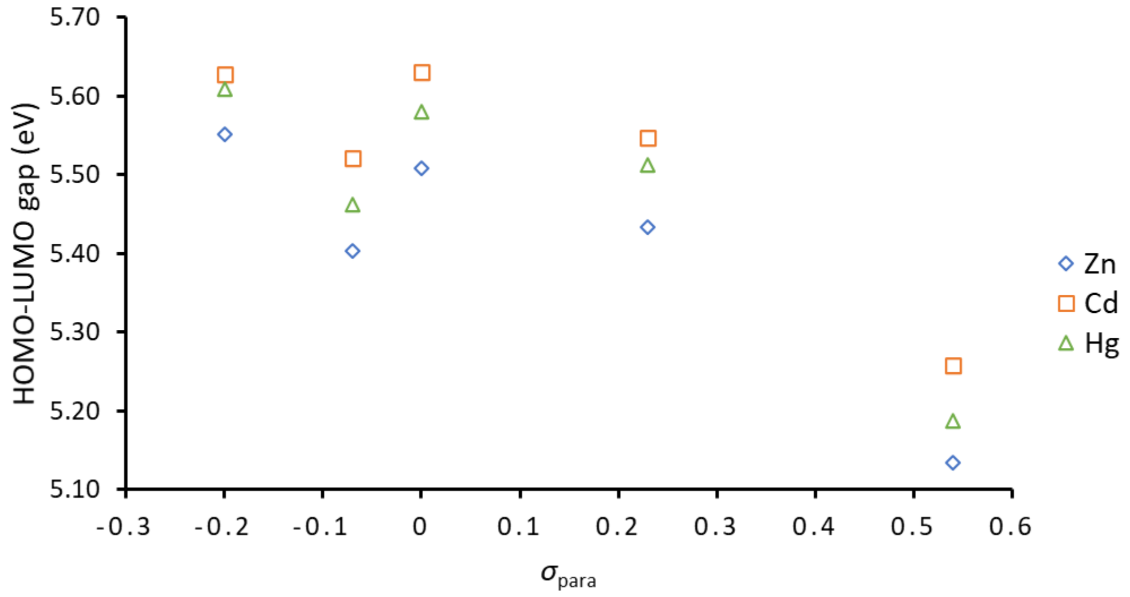

Figure S47: Graph of computed HOMO-LUMO gap (eV) versus Hammet constant of *para*-substituent (σ_{para}) for Group 12 *m*-terphenyl complexes.

S5.2.3. QTAIM Analysis

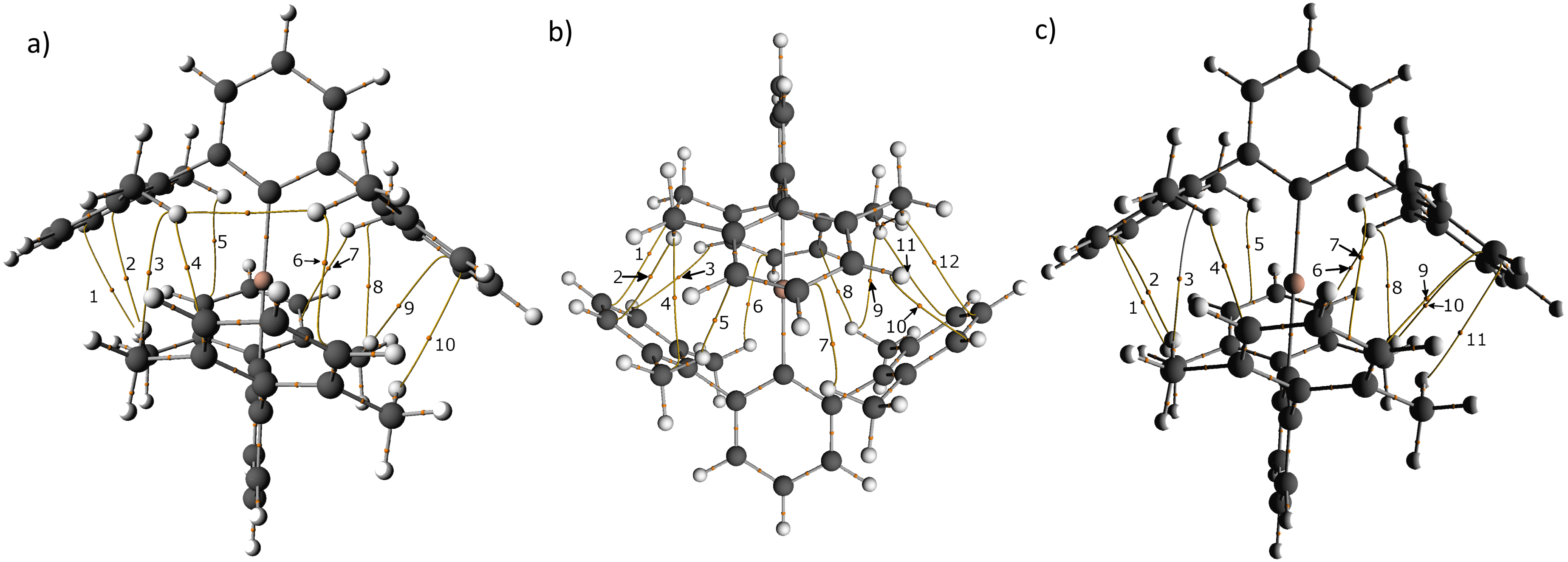

Figure S48: Plots of the optimized geometries of (H-Ar#)$_2$M [a) M = Zn; b) M = Cd; c) M = Hg] with bond paths (yellow lines) and bond critical points (orange dots) superimposed upon the structures. Selected bond critical points corresponding to H···C interactions have been numbered and their properties are reported in Table S6. Plots generated using the MultiWFN 3.6 software package.[31]

Table S6: Properties of the electron density function at selected bond critical points for the complexes $(H\text{-}Ar^{\#})_2M$ (M = Zn, Cd, Hg). For numbering scheme of the bond critical points, see Figure S48. All values given in standard atomic units.

Metal	BCP	ρ_{BCP}	$\nabla^2\rho_{BCP}$	G_{BCP}	V_{BCP}	H_{BCP}	ε
Zn	1	5.83E-03	1.95E-02	3.91E-03	-2.96E-03	9.53E-04	1.77
	2	5.88E-03	1.81E-02	3.72E-03	-2.93E-03	7.93E-04	1.07
	3	5.81E-03	2.29E-02	4.60E-03	-3.47E-03	1.13E-03	0.99
	4	5.57E-03	1.73E-02	3.54E-03	-2.76E-03	7.86E-04	1.40
	5	6.22E-03	1.99E-02	4.05E-03	-3.13E-03	9.21E-04	1.21
	6	6.05E-03	2.01E-02	4.06E-03	-3.09E-03	9.63E-04	1.21
	7	5.74E-03	1.70E-02	3.48E-03	-2.72E-03	7.65E-04	1.13
	8	4.55E-03	1.91E-02	3.73E-03	-2.68E-03	1.04E-03	3.48
	9	5.96E-03	1.75E-02	3.61E-03	-2.83E-03	7.78E-04	1.10
	10	6.21E-03	1.97E-02	4.03E-03	-3.13E-03	9.05E-04	1.13
Cd	1	5.59E-03	1.75E-02	3.52E-03	-2.66E-03	8.62E-04	1.52
	2	5.01E-03	1.46E-02	2.97E-03	-2.29E-03	6.80E-04	0.78
	3	3.05E-03	1.09E-02	2.03E-03	-1.33E-03	6.96E-04	4.84
	4	3.55E-03	1.50E-02	2.85E-03	-1.96E-03	8.91E-04	7.45
	5	5.02E-03	1.47E-02	2.98E-03	-2.29E-03	6.88E-04	0.73
	6	5.27E-03	1.68E-02	3.35E-03	-2.49E-03	8.57E-04	1.66
	7	5.25E-03	1.68E-02	3.34E-03	-2.48E-03	8.62E-04	1.75
	8	5.27E-03	1.53E-02	3.13E-03	-2.43E-03	6.99E-04	0.88
	9	3.85E-03	1.59E-02	3.05E-03	-2.13E-03	9.19E-04	3.40
	10	3.11E-03	1.11E-02	2.07E-03	-1.37E-03	7.02E-04	5.48
	11	4.98E-03	1.46E-02	2.97E-03	-2.29E-03	6.83E-04	0.84
	12	5.32E-03	1.70E-02	3.40E-03	-2.54E-03	8.60E-04	1.64
Hg	1	5.38E-03	1.71E-02	3.41E-03	-2.55E-03	8.59E-04	1.56
	2	5.19E-03	1.52E-02	3.10E-03	-2.40E-03	7.06E-04	0.92
	3	3.86E-03	1.61E-02	3.10E-03	-2.16E-03	9.37E-04	3.54
	4	4.92E-03	1.46E-02	2.95E-03	-2.26E-03	6.92E-04	0.85
	5	5.40E-03	1.72E-02	3.44E-03	-2.57E-03	8.67E-04	1.63
	6	4.98E-03	1.47E-02	2.98E-03	-2.28E-03	6.92E-04	0.88
	7	5.53E-03	1.76E-02	3.52E-03	-2.64E-03	8.72E-04	1.63
	8	3.74E-03	1.57E-02	3.01E-03	-2.09E-03	9.22E-04	2.99
	9	2.74E-03	9.20E-03	1.71E-03	-1.11E-03	5.93E-04	1.19
	10	5.05E-03	1.49E-02	3.02E-03	-2.32E-03	6.99E-04	0.84
	11	5.30E-03	1.70E-02	3.39E-03	-2.52E-03	8.68E-04	1.68

S5.3. NMR Spectroscopic Calculations

S5.3.1. Methodology

The NMR calculations were carried out on both the fully optimized and H-atom optimized structures using the ReSpect program.[32–37] This program allows for a relativistic 4 component SCF treatment at the density functional level, from which NMR parameters may then be calculated via response theory. This relativistic program was utilized to allow calculations on the heavy elements present in these complexes. All calculations in this work use the KT2 density-functional approximation,[38] which was specially designed for the calculation of NMR shielding constants. For details of how these functionals are implemented for use in non-collinear spin-DFT (SDFT) see Komorovsky et al. and Ekström et al.[35,39] Jensen's pcS-*n* basis sets[40] have been employed for all atoms except Zn, Cd, and Hg (n = 1, 2); these basis sets were optimized for the calculation of NMR shielding constants and are utilized in the ReSpect program in an uncontracted form. The dyall-vdz and dyall-vtz basis sets were employed for the Zn, Cd, and Hg atoms.[41,42] The RI-J approximation is used for the computation of electron-repulsion integrals, using the default matching auxiliary basis set in the ReSpect basis library. London orbitals[43–45] (also known as gauge-including atomic orbitals (GIAOs)) were used to determine the magnetic response parameters and the restricted magnetic balance condition[33,34,37,46–49] was employed in the 4 component relativistic calculations. All calculations were performed using 32 threads on a single node with shared memory.

The calculations yield absolute shielding constants. To facilitate comparison with experiment the shielding constants for nuclei in magnetically equivalent environments were first averaged, then to obtain chemical shifts a reference shielding value was determined by constructing correlation plots between the calculated absolute shieldings and the

experimentally obtained shifts for each type of nucleus in each compound. Linear regression was then applied, with the intercept providing the required reference shielding value. For the ^{113}Cd and ^{199}Hg NMR linear regression could not be used since there is only one nucleus present, so in this case calculations were performed on the reference compounds Me_2Cd and Me_2Hg with each functional and basis set to provide a reference absolute shielding.

S5.3.2. Results

Table S7: Calculated paramagnetic and diamagnetic contributions to the shielding constant of H-9, Cd, and Hg nuclei for the *H-atom optimized* structures of all Group 12 complexes. Results shown for calculations run at the dyall-vdz/pcS-1 (all complexes) and dyall-vtz/pcS-2 (all complexes except M = Hg) levels.

			**dyall-vdz/pcS-1**		**dyall-vtz/pcS-2**	
**R group**	**Metal**	**Nucleus**	**Para.**	**Dia.**	**Para.**	**Dia.**
t-Bu	Zn	H-9	36.75	-6.98	40.86	-11.27
SiMe$_3$	Zn	H-9	38.82	-9.24	43.36	-14.01
H	Zn	H-9	35.61	-5.59	40.19	-10.31
Cl	Zn	H-9	34.78	-4.82	37.32	-7.42
CF$_3$	Zn	H-9	36.25	-6.20	40.46	-10.60
t-Bu	Cd	H-9	36.99	-7.08	42.21	-12.43
		Cd	4901.42	-762.80	4914.34	-822.65
SiMe$_3$	Cd	H-9	38.37	-8.68	41.85	-12.37
		Cd	4899.37	-765.03	4913.45	-825.14
H	Cd	H-9	35.20	-5.39	38.47	-8.81
		Cd	4893.12	-747.99	4908.11	-811.58
Cl	Cd	H-9	34.80	-4.94	38.24	-8.58
		Cd	4894.56	-731.18	4906.92	-801.58
CF$_3$	Cd	H-9	37.06	-7.25	41.50	-11.96
		Cd	4898.61	-720.81	4909.68	-786.56
t-Bu	Hg	H-9	37.09	-7.36	-	-
		Hg	10100.92	1602.29	-	-
SiMe$_3$	Hg	H-9	38.17	-8.15	-	-
		Hg	10086.39	1534.27	-	-
H	Hg	H-9	36.29	-6.38	-	-
		Hg	10104.57	1602.71	-	-
Cl	Hg	H-9	34.91	-5.04	-	-
		Hg	10092.21	1579.28	-	-
CF$_3$	Hg	H-9	36.38	-6.33	-	-
		Hg	10093.86	1677.86	-	-

Table S8: Calculated paramagnetic and diamagnetic contributions to the shielding constant of H-9, Cd, and Hg nuclei for the *fully geometry optimized* structures of all Group 12 complexes. Results shown for calculations run at the dyall-vdz/pcS-1 (all complexes) and dyall-vtz/pcS-2 (all complexes except M = Hg) levels.

			dyall-vdz/pcS-1		dyall-vtz/pcS-2	
**R group**	**Metal**	**Nucleus**	**Para.**	**Dia.**	**Para.**	**Dia.**
t-Bu	Zn	H-9	36.81	-6.90	41.02	-11.27
$SiMe_3$	Zn	H-9	37.63	-7.90	42.67	-13.14
H	Zn	H-9	35.52	-5.41	37.88	-7.77
Cl	Zn	H-9	35.06	-5.02	39.79	-10.01
CF_3	Zn	H-9	36.59	-6.75	40.72	-11.29
t-Bu	Cd	H-9	36.54	-6.73	40.39	-10.70
		Cd	4898.92	-731.90	4913.27	-789.94
$SiMe_3$	Cd	H-9	38.31	-8.66	41.79	-12.38
		Cd	4899.62	-752.48	4911.33	-814.39
H	Cd	H-9	34.94	-5.25	39.00	-9.33
		Cd	4894.20	-781.28	4907.67	-834.59
Cl	Cd	H-9	35.04	-5.19	38.50	-8.85
		Cd	4898.07	-746.07	4909.30	-809.79
CF_3	Cd	H-9	37.11	-7.00	41.20	-11.22
		Cd	4895.53	-731.61	4908.54	-790.34
t-Bu	Hg	H-9	36.53	-6.78	-	-
		Hg	10097.03	1646.34	-	-
$SiMe_3$	Hg	H-9	37.43	-7.59	-	-
		Hg	10075.06	1620.66	-	-
H	Hg	H-9	34.89	-4.97	-	-
		Hg	10087.80	1552.50	-	-
Cl	Hg	H-9	35.26	-5.51	-	-
		Hg	10096.35	1598.87	-	-
CF_3	Hg	H-9	36.22	-6.31	-	-
		Hg	10089.52	1675.07	-	-

Table S9: Experimental and calculated NMR shifts of the H-9, Cd, and Hg nuclei for the *H-atom optimized* structures of all Group 12 complexes. Results shown for calculations run at the dyall-vdz/pcS-1 (all complexes) and dyall-vtz/pcS-2 (all complexes except M = Hg) levels.

				dyall-vdz/pcS-1	dyall-vtz/pcS-2
**R group**	**Metal**	**Nucleus**	**Expt. Shift (ppm)**	**Calc. Shift (ppm)**	**Calc. Shift (ppm)**
t-Bu	Zn	H-9	1.83	1.75	1.77
$SiMe_3$	Zn	H-9	1.82	1.85	1.86
H	Zn	H-9	1.79	1.51	1.49
Cl	Zn	H-9	1.61	1.66	1.73
CF_3	Zn	H-9	1.55	1.59	1.63
t-Bu	Cd	H-9	1.84	1.78	1.80
		Cd	-225.89	-336.77	-289.83
$SiMe_3$	Cd	H-9	1.82	1.64	1.70
		Cd	-239.07	-332.49	-286.47
H	Cd	H-9	1.80	1.89	1.90
		Cd	-239.36	-343.29	-294.68
Cl	Cd	H-9	1.63	1.64	1.62
		Cd	-246.03	-361.52	-303.49
CF_3	Cd	H-9	1.56	1.55	1.54
		Cd	-265.21	-375.96	-321.28
t-Bu	Hg	H-9	1.83	1.81	-
		Hg	-642.81	-961.04	-
$SiMe_3$	Hg	H-9	1.81	1.66	-
		Hg	-674.91	-878.49	-
H	Hg	H-9	1.78	1.84	-
		Hg	-679.77	-965.12	-
Cl	Hg	H-9	1.61	1.64	-
		Hg	-695.04	-929.32	-
CF_3	Hg	H-9	1.54	1.59	-
		Hg	-745.00	-1029.56	-

Table S10: Experimental and calculated NMR shifts of the H-9, Cd, and Hg nuclei for the *fully geometry optimized* structures of all Group 12 complexes. Results shown for calculations run at the dyall-vdz/pcS-1 (all complexes) and dyall-vtz/pcS-2 (all complexes except M = Hg) levels.

				dyall-vdz/pcS-1	dyall-vtz/pcS-2
**R group**	**Metal**	**Nucleus**	**Expt. Shift (ppm)**	**Calc. Shift (ppm)**	**Calc. Shift (ppm)**
t-Bu	Zn	H-9	1.83	1.68	1.72
$SiMe_3$	Zn	H-9	1.82	1.77	1.82
H	Zn	H-9	1.79	1.46	1.49
Cl	Zn	H-9	1.61	1.66	1.62
CF_3	Zn	H-9	1.55	1.53	1.44
t-Bu	Cd	H-9	1.84	1.81	1.90
		Cd	-225.89	-365.17	-321.48
$SiMe_3$	Cd	H-9	1.82	1.67	1.69
		Cd	-239.07	-345.30	-295.09
H	Cd	H-9	1.80	1.82	1.84
		Cd	-239.36	-311.07	-271.24
Cl	Cd	H-9	1.63	1.65	1.65
		Cd	-246.03	-350.16	-297.66
CF_3	Cd	H-9	1.56	1.63	1.65
		Cd	-265.21	-362.07	-316.35
t-Bu	Hg	H-9	1.83	1.76	-
		Hg	-642.81	-1001.21	-
$SiMe_3$	Hg	H-9	1.81	1.79	-
		Hg	-674.91	-953.55	-
H	Hg	H-9	1.78	1.87	-
		Hg	-679.77	-898.13	-
Cl	Hg	H-9	1.61	1.55	-
		Hg	-695.04	-953.06	-
CF_3	Hg	H-9	1.54	1.55	-
		Hg	-745.00	-1022.43	-

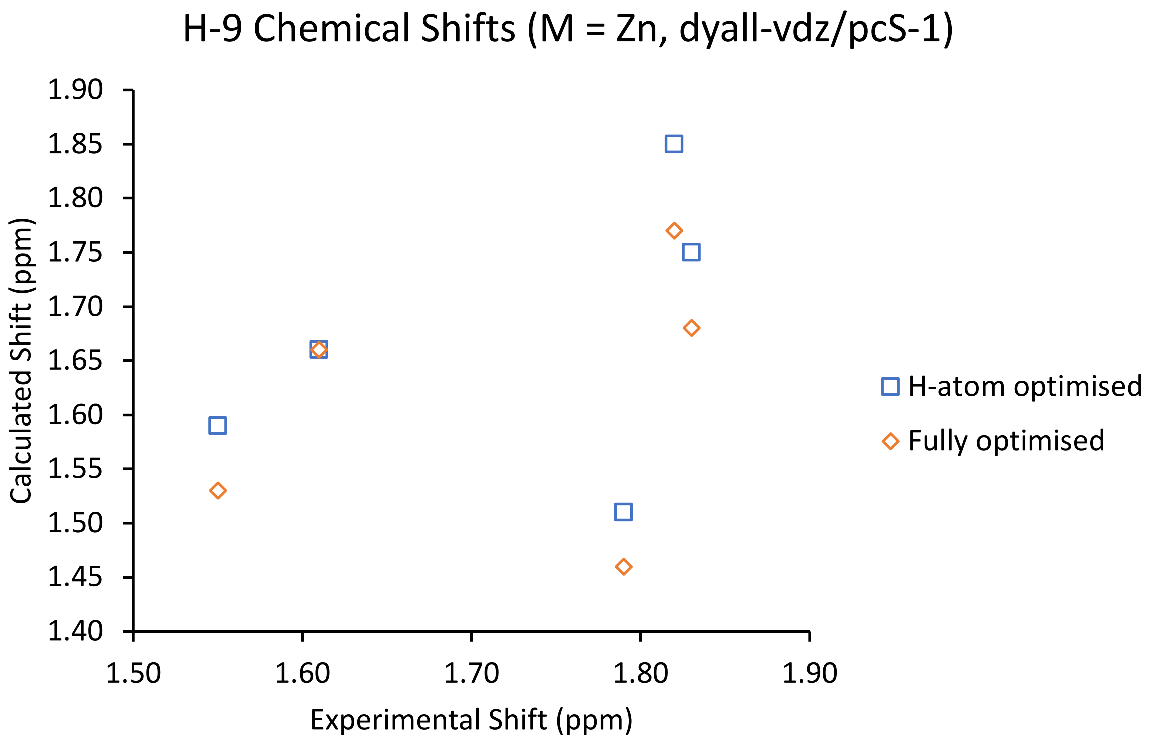

Figure S49: Plot of experimental vs. calculated H-9 ^{1}H NMR shift for (R-Ar$^{\#}$)$_2$Zn complexes (R = *t*-Bu, $SiMe_3$, H, Cl, CF_3) computed at the dyall-vdz/pcS-1 level for both H-atom optimized and fully geometry optimized structures.

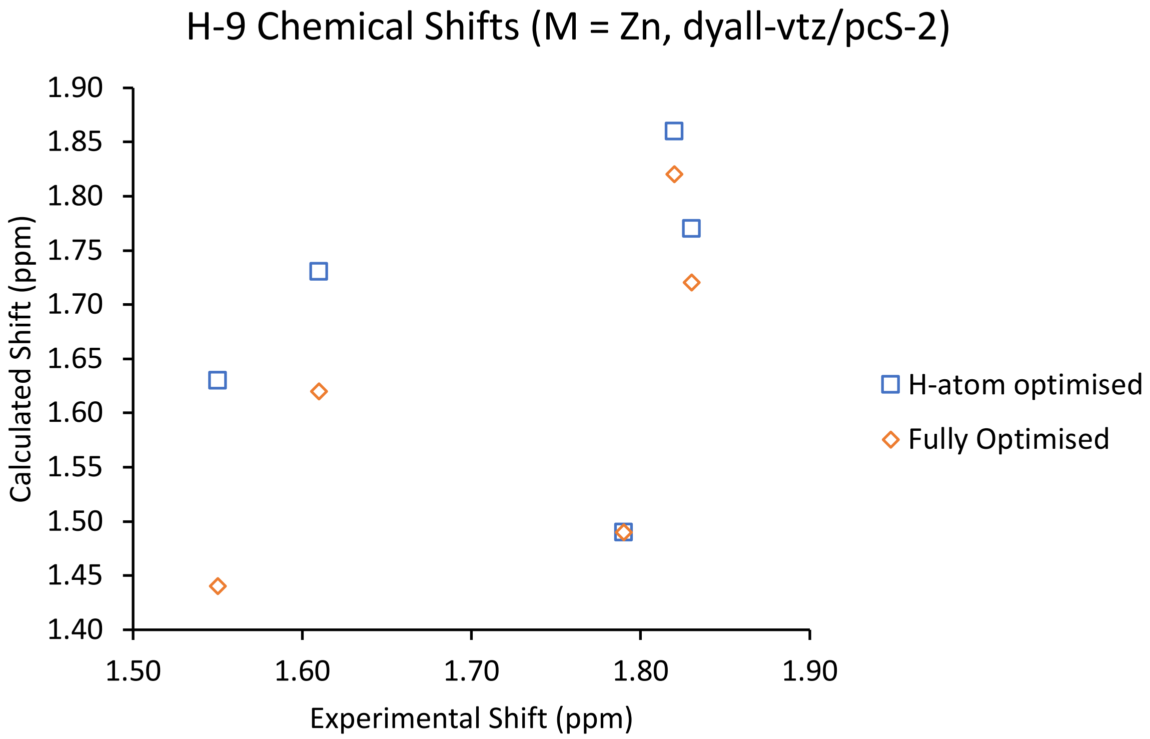

Figure S50: Plot of experimental vs. calculated H-9 ^{1}H NMR shift for (R-Ar$^{\#}$)$_2$Zn complexes (R = *t*-Bu, $SiMe_3$, H, Cl, CF_3) computed at the dyall-vtz/pcS-2 level for both H-atom optimized and fully geometry optimized structures.

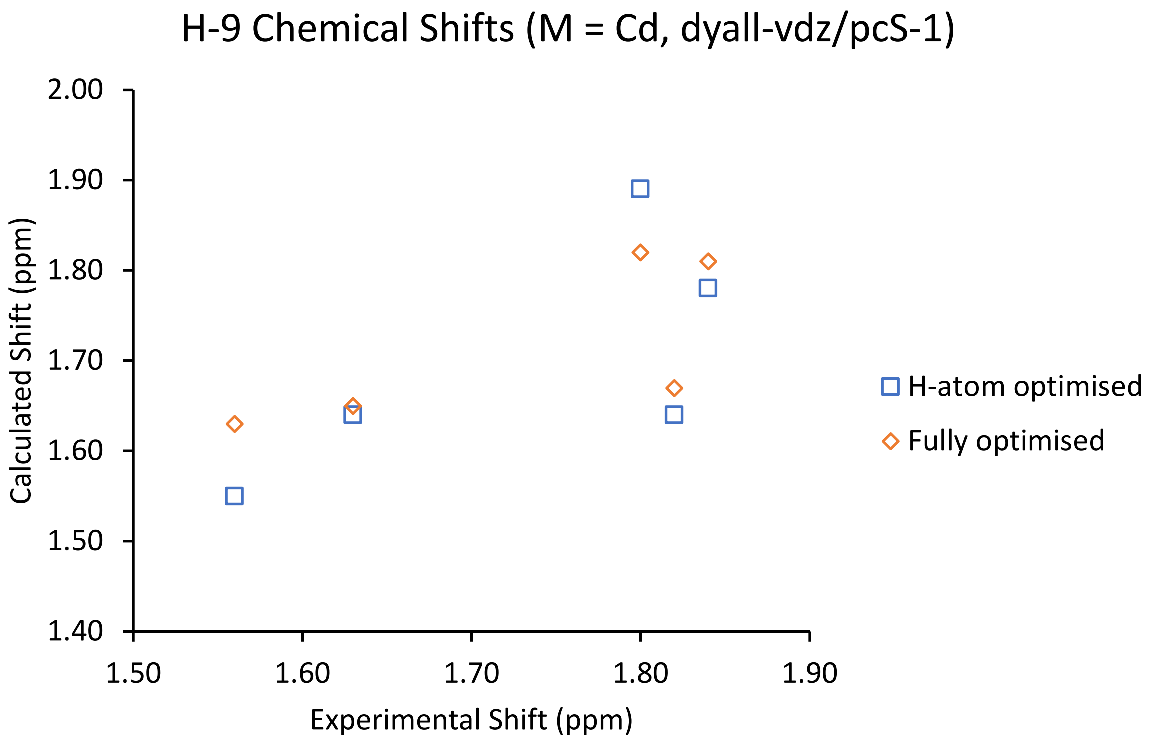

Figure S51: Plot of experimental vs. calculated H-9 ^{1}H NMR shift for (R-Ar$^{\#}$)$_2$Cd complexes (R = *t*-Bu, $SiMe_3$, H, Cl, CF_3) computed at the dyall-vdz/pcS-1 level for both H-atom optimized and fully geometry optimized structures.

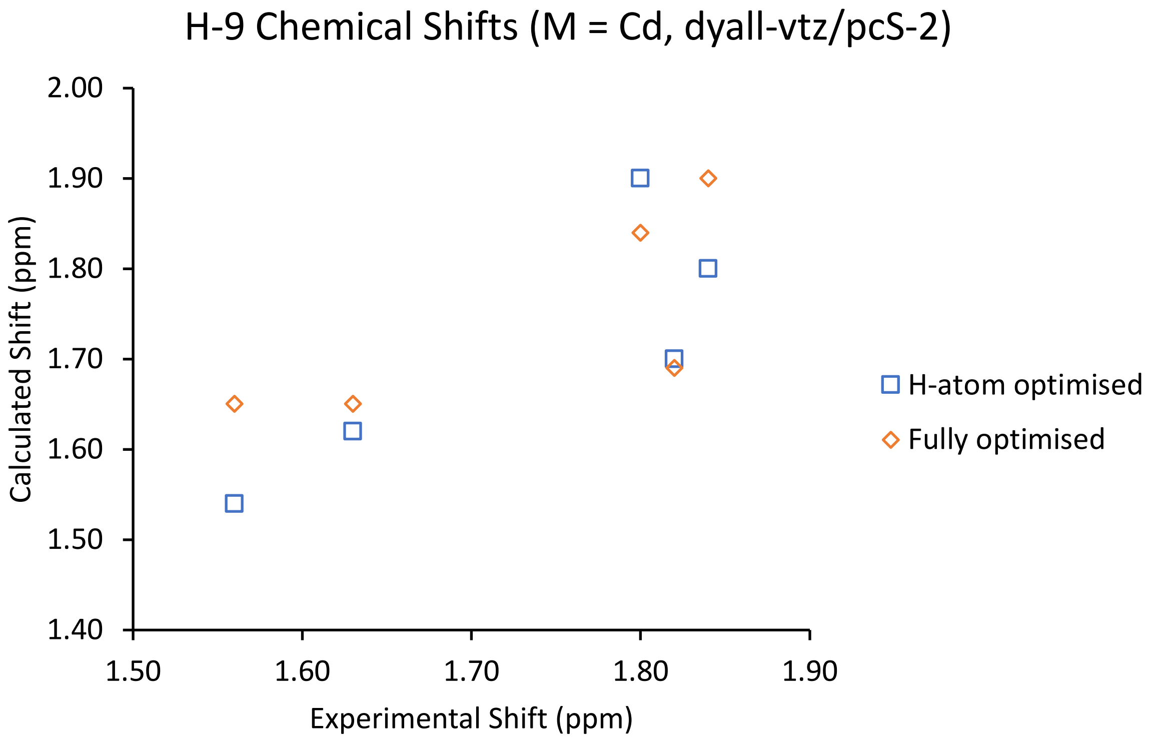

Figure S52: Plot of experimental vs. calculated H-9 ^{1}H NMR shift for (R-Ar$^{\#}$)$_2$Cd complexes (R = *t*-Bu, $SiMe_3$, H, Cl, CF_3) computed at the dyall-vtz/pcS-2 level for both H-atom optimized and fully geometry optimized structures.

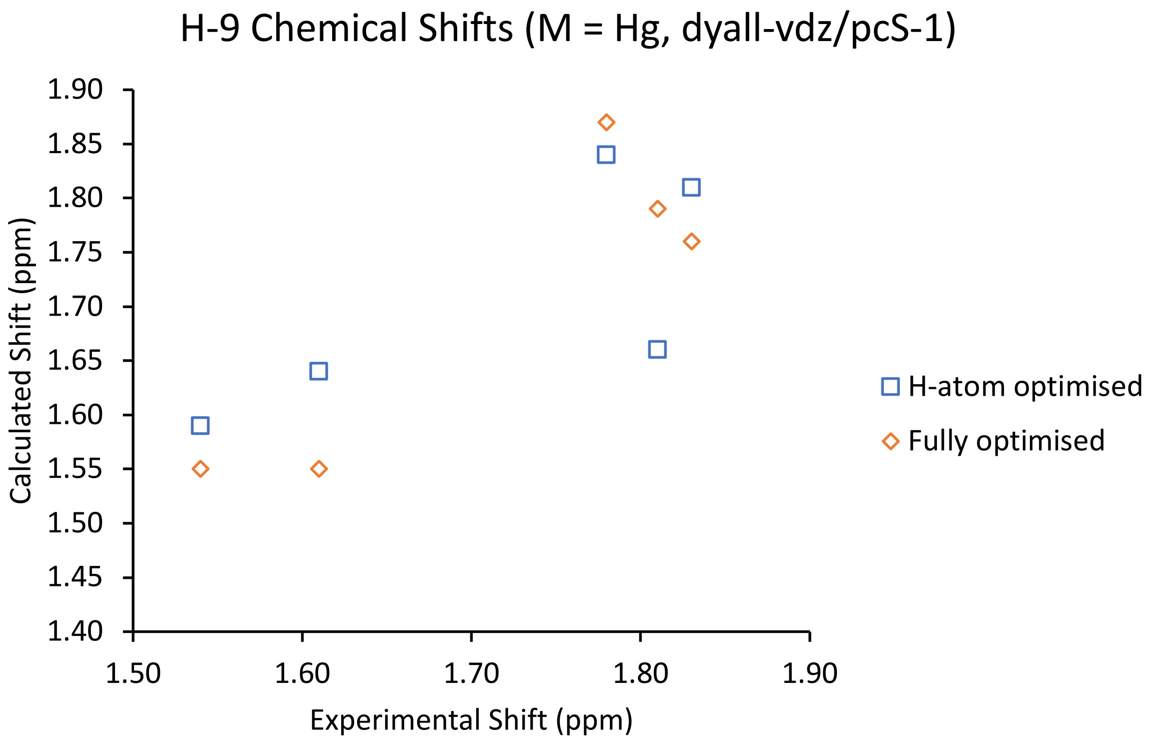

Figure S53: Plot of experimental vs. calculated H-9 ^{1}H NMR shift for (R-Ar$^{\#}$)$_2$Hg complexes (R = *t*-Bu, $SiMe_3$, H, Cl, CF_3) computed at the dyall-vdz/pcS-1 level for both H-atom optimized and fully geometry optimized structures.

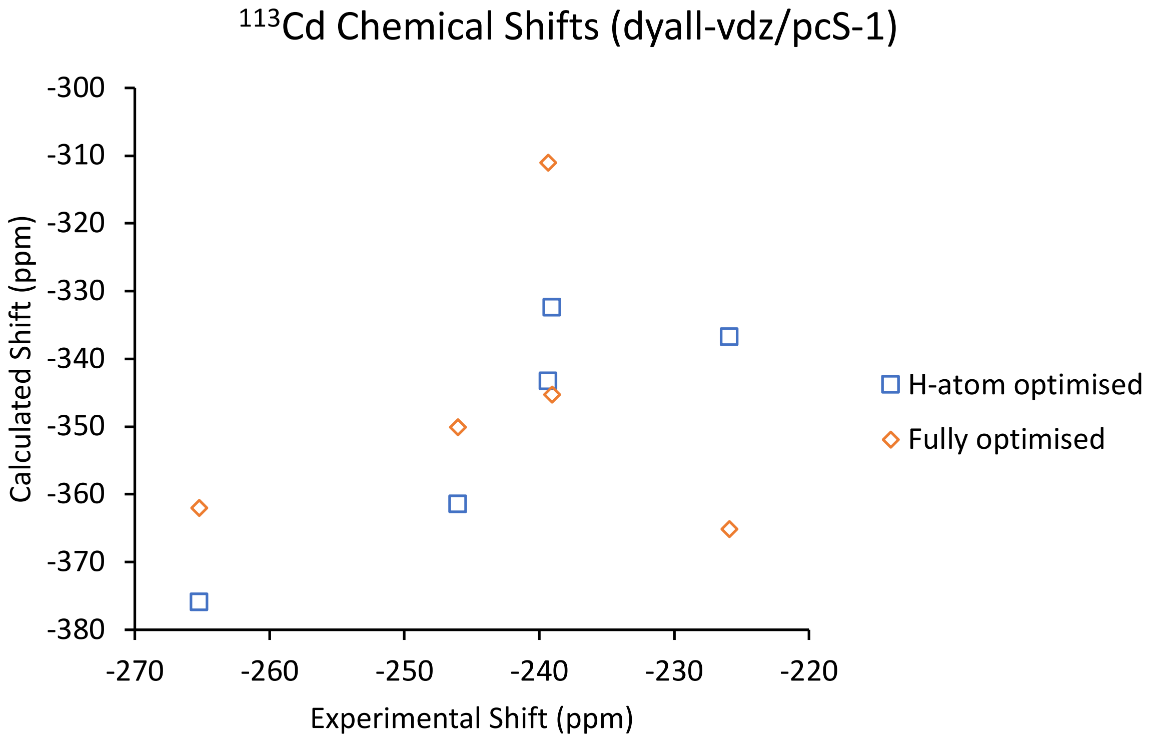

Figure S54: Plot of experimental vs. calculated ^{113}Cd NMR shift for (R-Ar$^{\#}$)$_2$Cd complexes (R = *t*-Bu, $SiMe_3$, H, Cl, CF_3) computed at the dyall-vdz/pcS-1 level for both H-atom optimized and fully geometry optimized structures.

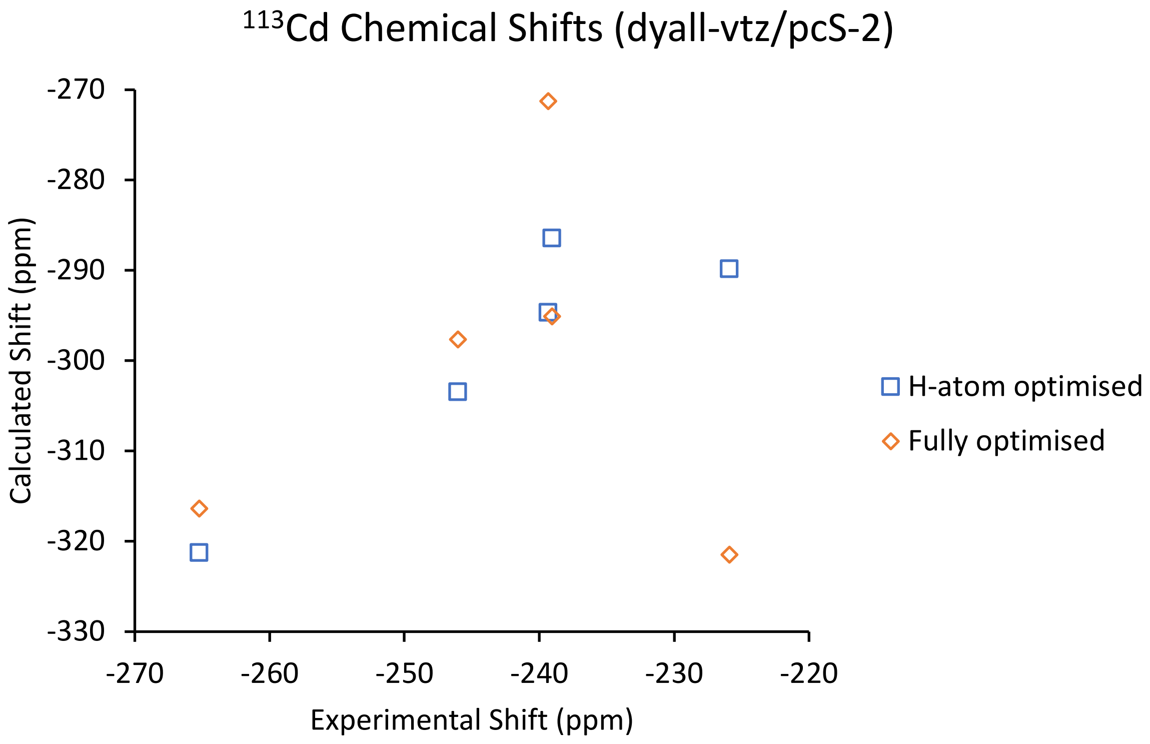

Figure S55: Plot of experimental vs. calculated ^{113}Cd NMR shift for (R-Ar$^{\#}$)$_2$Cd complexes (R = *t*-Bu, $SiMe_3$, H, Cl, CF_3) computed at the dyall-vtz/pcS-2 level for both H-atom optimized and fully geometry optimized structures.

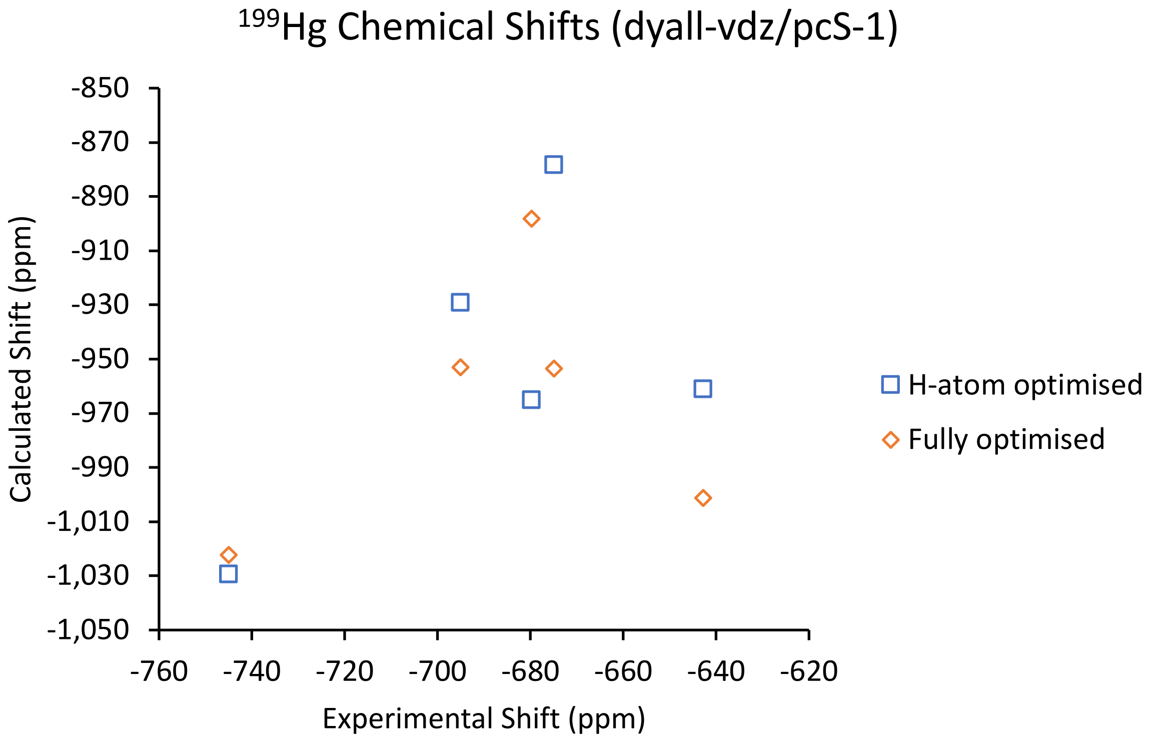

Figure S56: Plot of experimental vs. calculated ^{199}Hg NMR shift for (R-Ar$^{\#}$)$_2$Hg complexes (R = *t*-Bu, $SiMe_3$, H, Cl, CF_3) computed at the dyall-vdz/pcS-1 level for both H-atom optimized and fully geometry optimized structures.

S6. References

(1) Boudjouk, P.; So, J.-H.; Ackermann, M. N.; Hawley, S. E.; Turk, B. E. Solvated and Unsolvated Anhydrous Metal Chlorides from Metal Chloride Hydrates. *Inorganic Syntheses*. January 1992, pp 108–111.

(2) Valentine, A. J.; Geer, A. M.; Taylor, L. J.; Teale, A. M.; Wood, K. E.; Williams, H. E. L.; Lewis, W.; Argent, S. P.; McMaster, J.; Kays, D. L. Structural and Electronic Studies of Substituted *m*-Terphenyl Lithium Complexes. *Dalton Trans.* **2021**, *50* (2), 722–728.

(3) Cosier, J.; Glazer, A. M. A Nitrogen-Gas-Stream Cryostat for General X-Ray Diffraction Studies. *J. Appl. Cryst.* **1986**, *19*, 105–107.

(4) *CrysAlisPRO*; Oxford Diffraction/Agilent Technologies UK Ltd: Yarnton, England.

(5) Spek, A. L. PLATON SQUEEZE: A Tool for the Calculation of the Disordered Solvent Contribution to the Calculated Structure Factors. *Acta Crystallogr. C* **2015**, *71*, 9–18.

(6) Dolomanov, O. V; Bourhis, L. J.; Gildea, R. J.; Howard, J. A. K.; Puschmann, H. *OLEX2*: A Complete Structure Solution, Refinement and Analysis Program. *J. Appl. Cryst.* **2009**, *42*, 339–341.

(7) Sheldrick, G. M. *SHELXT* – Integrated Space-Group and Crystal-Structure Determination. *Acta Crystallogr. Sect. A* **2015**, *71* (1), 3–8.

(8) Sheldrick, G. M. Crystal Structure Refinement with *SHELXL*. *Acta Crystallogr. Sect. C* **2015**, *71* (1), 3–8.

(9) Blundell, T. J.; Hastings, F. R.; Gridley, B. M.; Moxey, G. J.; Lewis, W.; Blake, A. J.; Kays, D. L. Ligand Influences on Homoleptic Group 12 M-Terphenyl Complexes. *Dalton Trans.* **2014**, *43* (38), 14257–14264.

(10) Neese, F. The ORCA Program System. *Wiley Interdiscip. Rev. Comput. Mol. Sci.* **2012**, *2* (1), 73–78.

(11) Neese, F. Software Update: The ORCA Program System, Version 4.0. *Wiley Interdiscip. Rev. Comput. Mol. Sci.* **2018**, *8* (1), e1327.

(12) Becke, A. D. Density-Functional Exchange-Energy Approximation with Correct Asymptotic Behavior. *Phys. Rev. A* **1988**, *38* (6), 3098–3100.

(13) Perdew, J. P. Density-Functional Approximation for the Correlation Energy of the Inhomogeneous Electron Gas. *Phys. Rev. B* **1986**, *33* (12), 8822–8824.

(14) Dunlap, B. I.; Connolly, J. W. D.; Sabin, J. R. On the Applicability of LCAO-Xα Methods to Molecules Containing Transition Metal Atoms: The Nickel Atom and Nickel Hydride. *Int. J. Quantum Chem.* **1977**, *12* (S11), 81–87.

(15) Dunlap, B. I.; Connolly, J. W. D.; Sabin, J. R. On Some Approximations in Applications of Xα Theory. *J. Chem. Phys.* **1979**, *71* (8), 3396–3402.

(16) Van Lenthe, E.; Snijders, J. G.; Baerends, E. J. The Zero-Order Regular Approximation for Relativistic Effects: The Effect of Spin-Orbit Coupling in Closed Shell Molecules. *J. Chem. Phys.* **1996**, *105* (15), 6505–6516.

(17) Weigend, F.; Furche, F.; Ahlrichs, R. Gaussian Basis Sets of Quadruple Zeta Valence Quality for Atoms H-Kr. *J. Chem. Phys.* **2003**, *119* (24), 12753–12762.

(18) Schäfer, A.; Horn, H.; Ahlrichs, R. Fully Optimized Contracted Gaussian Basis Sets for Atoms Li to Kr. *J. Chem. Phys.* **1992**, *97* (4), 2571–2577.

(19) Schäfer, A.; Huber, C.; Ahlrichs, R. Fully Optimized Contracted Gaussian Basis Sets of Triple Zeta Valence Quality for Atoms Li to Kr. *J. Chem. Phys.* **1994**, *100* (8), 5829–5835.

(20) Pantazis, D. A.; Chen, X. Y.; Landis, C. R.; Neese, F. All-Electron Scalar Relativistic

Basis Sets for Third-Row Transition Metal Atoms. *J. Chem. Theory Comput.* **2008**, *4* (6), 908–919.
(21) Weigend, F. Accurate Coulomb-Fitting Basis Sets for H to Rn. *Phys. Chem. Chem. Phys.* **2006**, *8* (9), 1057–1065.
(22) Pantazis, D. A.; Neese, F. All-Electron Scalar Relativistic Basis Sets for the Lanthanides. *J. Chem. Theory Comput.* **2009**, *5* (9), 2229–2238.
(23) Pantazis, D. A.; Neese, F. All-Electron Scalar Relativistic Basis Sets for the Actinides. *J. Chem. Theory Comput.* **2011**, *7* (3), 677–684.
(24) Pantazis, D. A.; Neese, F. All-Electron Scalar Relativistic Basis Sets for the 6p Elements. *Theor. Chem. Acc.* **2012**, *131* (11), 1292.
(25) Grimme, S.; Antony, J.; Ehrlich, S.; Krieg, H. A Consistent and Accurate Ab Initio Parametrization of Density Functional Dispersion Correction (DFT-D) for the 94 Elements H-Pu. *J. Chem. Phys.* **2010**, *132* (15), 154104.
(26) Grimme, S.; Ehrlich, S.; Goerigk, L. Effect of the Damping Function in Dispersion Corrected Density Functional Theory. *J. Comput. Chem.* **2011**, *32* (7), 1456–1465.
(27) Perdew, J. P.; Burke, K.; Ernzerhof, M. Generalized Gradient Approximation Made Simple. *Phys. Rev. Lett.* **1996**, *77* (18), 3865–3868.
(28) Adamo, C.; Barone, V. Toward Reliable Density Functional Methods without Adjustable Parameters: The PBE0 Model. *J. Chem. Phys.* **1999**, *110* (13), 6158–6170.
(29) Neese, F.; Wennmohs, F.; Hansen, A.; Becker, U. Efficient, Approximate and Parallel Hartree–Fock and Hybrid DFT Calculations. A ‘Chain-of-Spheres’ Algorithm for the Hartree–Fock Exchange. *Chem. Phys.* **2009**, *356* (1), 98–109.
(30) Weigend, F.; Ahlrichs, R. Balanced Basis Sets of Split Valence, Triple Zeta Valence and Quadruple Zeta Valence Quality for H to Rn: Design and Assessment of Accuracy. *Phys. Chem. Chem. Phys.* **2005**, *7* (18), 3297–3305.
(31) Lu, T.; Chen, F. Multiwfn: A Multifunctional Wavefunction Analyzer. *J. Comput. Chem.* **2012**, *33* (5), 580–592.
(32) Repisky, M.; Komorovsky, S.; Malkin, V. G.; Malkina, O. L.; Kaupp, M.; Ruud, K.; with contributions from R. Bast; Di Remigio, R.; Ekström, U.; Kadek, M.; Knecht, S.; Konecny, L.; Malkin, E.; Malkin Ondik, I. *ReSpect 5.1.0, Relativistic Spectroscopy DFT Program*; 5.1.0; http://www.respectprogram.org, 2019.
(33) Komorovský, S.; Repiský, M.; Malkina, O. L.; Malkin, V. G.; Malkin Ondík, I.; Kaupp, M. A Fully Relativistic Method for Calculation of Nuclear Magnetic Shielding Tensors with a Restricted Magnetically Balanced Basis in the Framework of the Matrix Dirac–Kohn–Sham Equation. *J. Chem. Phys.* **2008**, *128* (10), 104101.
(34) Komorovský, S.; Repiský, M.; Malkina, O. L.; Malkin, V. G. Fully Relativistic Calculations of NMR Shielding Tensors Using Restricted Magnetically Balanced Basis and Gauge Including Atomic Orbitals. *J. Chem. Phys.* **2010**, *132* (15), 154101.
(35) Komorovsky, S.; Repisky, M.; Malkin, E.; Demissie, T. B.; Ruud, K. Four-Component Relativistic Density-Functional Theory Calculations of Nuclear Spin–Rotation Constants: Relativistic Effects in p-Block Hydrides. *J. Chem. Theory Comput.* **2015**, *11* (8), 3729–3739.
(36) Repisky, M.; Komorovsky, S.; Bast, R.; Ruud, K. Relativistic Calculations of Nuclear Magnetic Resonance Parameters. In *New Developments in NMR No. 6*; Karol Jackowski, M. J., Ed.; The Royal Society of Chemistry, 2016; pp 267–303.
(37) Repiský, M.; Komorovský, S.; Malkina, O. L.; Malkin, V. G. Restricted Magnetically Balanced Basis Applied for Relativistic Calculations of Indirect Nuclear Spin–Spin Coupling Tensors in the Matrix Dirac–Kohn–Sham Framework. *Chem. Phys.* **2009**, *356* (1), 236–242.
(38) Keal, T. W.; Tozer, D. J. The Exchange-Correlation Potential in Kohn–Sham Nuclear

Magnetic Resonance Shielding Calculations. *J. Chem. Phys.* **2003**, *119* (6), 3015–3024.
(39) Ekström, U.; Visscher, L.; Bast, R.; Thorvaldsen, A. J.; Ruud, K. Arbitrary-Order Density Functional Response Theory from Automatic Differentiation. *J. Chem. Theory Comput.* **2010**, *6* (7), 1971–1980.
(40) Jensen, F. Basis Set Convergence of Nuclear Magnetic Shielding Constants Calculated by Density Functional Methods. *J. Chem. Theory Comput.* **2008**, *4* (5), 719–727.
(41) Dyall, K. G. Relativistic Double-Zeta, Triple-Zeta, and Quadruple-Zeta Basis Sets for the 4d Elements Y-Cd. *Theor. Chem. Acc.* **2007**, *117* (4), 483–489.
(42) Dyall, K. G.; Gomes, A. S. P. Revised Relativistic Basis Sets for the 5d Elements Hf-Hg. *Theor. Chem. Acc.* **2009**, *125* (1), 97–100.
(43) London, F. Théorie Quantique Des Courants Interatomiques Dans Les Combinaisons Aromatiques. *J. Phys. Radium* **1937**, *8* (10), 397–409.
(44) Ditchfield, R. Theoretical Studies of Magnetic Shielding in H_2O and $(H_2O)_2$. *J. Chem. Phys.* **1976**, *65* (8), 3123–3133.
(45) Ditchfield, R. Self-Consistent Perturbation Theory of Diamagnetism. *Mol. Phys.* **1974**, *27* (4), 789–807.
(46) Kutzelnigg, W. Relativistic Corrections to Magnetic Properties. *J. Comput. Chem.* **1999**, *20* (12), 1199–1219.
(47) Kutzelnigg, W. Diamagnetism in Relativistic Theory. *Phys. Rev. A* **2003**, *67* (3), 032109.
(48) Cheng, L.; Xiao, Y.; Liu, W. Four-Component Relativistic Theory for Nuclear Magnetic Shielding: Magnetically Balanced Gauge-Including Atomic Orbitals. *J. Chem. Phys.* **2009**, *131* (24), 244113.
(49) Reynolds, R. D.; Shiozaki, T. Fully Relativistic Self-Consistent Field under a Magnetic Field. *Phys. Chem. Chem. Phys.* **2015**, *17* (22), 14280–14283.
